# Dynamic patterning by the *Drosophila* pair-rule network reconciles long-germ and short-germ segmentation

**DOI:** 10.1371/journal.pbio.2002439

**Published:** 2017-09-27

**Authors:** Erik Clark

**Affiliations:** Laboratory for Development and Evolution, Department of Zoology, University of Cambridge, Cambridge, United Kingdom; New York University, United States of America

## Abstract

*Drosophila* segmentation is a well-established paradigm for developmental pattern formation. However, the later stages of segment patterning, regulated by the “pair-rule” genes, are still not well understood at the system level. Building on established genetic interactions, I construct a logical model of the *Drosophila* pair-rule system that takes into account the demonstrated stage-specific architecture of the pair-rule gene network. Simulation of this model can accurately recapitulate the observed spatiotemporal expression of the pair-rule genes, but only when the system is provided with dynamic “gap” inputs. This result suggests that dynamic shifts of pair-rule stripes are essential for segment patterning in the trunk and provides a functional role for observed posterior-to-anterior gap domain shifts that occur during cellularisation. The model also suggests revised patterning mechanisms for the parasegment boundaries and explains the aetiology of the *even-skipped* null mutant phenotype. Strikingly, a slightly modified version of the model is able to pattern segments in either simultaneous or sequential modes, depending only on initial conditions. This suggests that fundamentally similar mechanisms may underlie segmentation in short-germ and long-germ arthropods.

## Author summary

Segmentation in insects involves the division of the body into several repetitive units. In *Drosophila* embryos, all segments are patterned rapidly and simultaneously during early development, in a process known as “long-germ” embryogenesis. In contrast, many insect embryos retain an ancestral or “short-germ” mode of development, in which segments are patterned sequentially, from head to tail, over a period of time. In both types of embryo, the patterning of segment boundaries is regulated by a network of so-called “pair-rule” genes. These networks are thought to be quite divergent due to the different expression patterns observed for the pair-rule genes in each case: regularly spaced arrays of transient stripes in *Drosophila*, and dynamic expression within a posterior “segment addition zone” in short-germ insects. However, even in *Drosophila*, a clear understanding of pair-rule patterning has been lacking. Here, I make a computational model of the *Drosophila* pair-rule network and use simulations to explore how segmentation works. Surprisingly, I find that *Drosophila* segment patterning relies on pair-rule gene expression moving across cells over time. This conclusion differs from older models of pair-rule patterning but is consistent with the subtly dynamic nature of pair-rule stripes in real embryos, previously described in quantitative studies. I conclude that long-germ and short-germ segmentation involve similar expression dynamics at the level of individual cells, even though they look very different at the level of whole tissues. This suggests that the gene networks involved may be much more conserved than previously thought.

## Introduction

Like other arthropods, the fruit fly *Drosophila melanogaster* has a segmented body plan. This segmental pattern is laid down in the embryo during the first 3 hours of development. During this time, the anteroposterior (AP) axis of the blastoderm is progressively patterned down to cellular-level resolution by an elaborate, multi-tiered network of genes and their encoded transcription factors [1,2]. These genes were first identified in a landmark genetic screen [3,4], and their regulatory interactions have subsequently been dissected by 3 decades of genetic experiments. Along the way, this body of research has revealed many fundamental principles of transcriptional regulation [5], and *Drosophila* segmentation remains a central model for developmental systems biology today.

Much of the “heavy lifting” of segment patterning is carried out by the so-called “pair-rule” genes, which make up the penultimate tier of the *Drosophila* segmentation cascade. The pair-rule genes are the first genes to be expressed in spatially periodic patterns in the *Drosophila* embryo and are collectively responsible for patterning the expression of the “segment-polarity” genes, which organise and maintain segmentally reiterated compartment boundaries termed “parasegment boundaries”. Notably, this involves transducing a double segment pattern of early pair-rule gene expression, in which each set of stripes is offset slightly from the others, into a single-segment pattern of segment-polarity gene expression, in which most genes are expressed in discrete, non-overlapping domains [6–8].

There are 7 canonical pair-rule genes: *hairy* [9], *even-skipped* (*eve*) [10], *runt* [11], *fushi tarazu* (*ftz*) [12], *odd-skipped* (*odd*) [13], *paired* (*prd*) [14], and *sloppy-paired* (*slp*) [15]. 5 of these genes (*hairy*, *eve*, *runt*, *ftz*, and *odd*) are known as the “primary” pair-rule genes because they are expressed earlier than the 2 “secondary” pair-rule genes, *prd* and *slp* [16]. (Note that these terms have a somewhat tortuous history, and older literature will classify the genes differently.)

Each of the primary pair-rule genes is initially patterned by spatial inputs from the upstream tier of transcription factors, encoded by the “gap” genes, which are expressed in broad, overlapping AP domains during cellularisation [17]. This patterning occurs in an ad hoc manner, with specific combinations of gap factors regulating the expression of particular pair-rule stripes through discrete “stripe-specific” enhancer elements [18–21], which act additively with one another. For certain pair-rule genes, such as *eve*, this regulation is sufficient to generate an overall pattern of 7 equally spaced stripes along the future trunk of the embryo [16,22–24]. For other pair-rule genes, such as *odd*, the gap-driven pattern is irregular and may have missing stripes [16]. In these cases, the initial patterns are regularised by cross-regulatory “zebra” enhancer elements [25–27], which take periodic inputs from other pair-rule factors and yield periodic outputs. Similar zebra elements are responsible for driving the periodic expression of the secondary pair-rule genes, which turn on after the primary pair-rule patterns have refined [16,28].

At gastrulation, the segment-polarity genes turn on, activated by a broadly expressed transcription factor, Odd-paired (Opa) [29], and spatially regulated by the pair-rule genes [6,7,30,31]. Opa activity also “rewires” the regulatory interactions between the pair-rule genes, causing several of their expression patterns to transition dynamically from double- to single-segment periodicity (i.e., from 7 stripes to 14 stripes) [32]. These pair-rule factors (and/or their paralogs) then play roles in the segment-polarity network, which also contains several components of the Wingless (Wg) and Hedgehog signalling pathways [33–37].

The *Drosophila* gap gene network has been used frequently in recent years as a case study for the application of dynamical systems [38–40] and information theory [41–43] approaches to developmental patterning, but the pair-rule network has received little system-level attention. Indeed, the most recent models of pair-rule patterning [8,44] date from more than 10 years ago. Since these were published, 3 important discoveries have been made about segment patterning, all of which challenge established assumptions about the *Drosophila* segmentation cascade and all of which concern the pair-rule genes in some way. So long as the pair-rule network remains poorly understood, key questions raised by these findings will go unanswered.

The first discovery is from comparative studies in other arthropod embryos. *Drosophila* is a “long-germ” embryo, patterning almost all of its segments simultaneously in the blastoderm prior to germ-band extension [45]. However, the ancestral mode of arthropod development is “short-germ” embryogenesis, in which segmentation is sequential and coordinated with germ-band elongation [46–48]. Orthologs of the pair-rule genes play a key role in segment patterning in all arthropods studied thus far (for example, [49–52]), but in short-germ embryos, their expression has been shown to oscillate in a posterior “segment addition zone” throughout germ-band extension [53–55]. This periodic dynamic expression indicates that in these organisms, they are either components of or entrained by a segmentation “clock” [56]. How the expression of the pair-rule genes in long-germ embryos such as *Drosophila* relates to their expression in short-germ embryos (for example, the model beetle, *Tribolium castaneum*) is unclear. It is thus not understood how long-germ segmentation was derived from short-germ segmentation, an important evolutionary transition that has occurred multiple times independently within the higher insects [57].

The second discovery stems from quantitative studies of *Drosophila* segmentation gene dynamics. These studies have revealed that the domains of gap gene expression in the trunk of the embryo shift anteriorly across the blastoderm over the course of nuclear division cycle 14 (cellularisation) [58–60]. The shifts are mirrored downstream in similarly shifting expression of the pair-rule stripes [59,61], a finding that is at odds with existing models of segment patterning, which assume these stripes to be static domains [6,8,44,62–64]. While we know that these subtle shifts are ultimately driven by feedback interactions within the gap gene network [38,39,65–67], their functional role (if any) remains unclear.

The final key finding relates to the structure of the pair-rule network itself. In a recent paper on the pair-rule network [32], Michael Akam and I showed that many of the regulatory interactions between the pair-rule genes are temporally regulated (by Opa, as described above). We argued that the pair-rule network is best viewed as 2 distinct networks, 1 operating during cellularisation and 1 during gastrulation, each with a specific topology and resultant dynamics. Analysing the “early” (cellularisation-stage) and “late” (gastrulation-stage) pair-rule networks separately should lead to a better understanding of pair-rule patterning and might also reveal why the network shows this bipartite organisation in the first place.

In this paper, I present a new model of the pair-rule system, which incorporates the stage-specific architecture of the pair-rule network. I take the set of identified genetic interactions between the pair-rule genes as a starting assumption, formalise them in a Boolean logical model, and use dynamical simulations to analyse how they collectively lead to complex pattern formation. I find that gap-mediated kinematic shifts are required for correctly phasing the pair-rule stripes, something that proves crucial for downstream segment patterning. I also find that graded Eve activity is not strictly necessary for pair-rule patterning, and I explain the aetiology of the surprisingly severe *eve* null mutant phenotype. Finally, I show that a slightly modified version of the *Drosophila* pair-rule network gains the capacity to pattern in both simultaneous and sequential modes, conceptually reconciling long- and short-germ segmentation.

## Results

### Topology of the pair-rule network

**Fig 1:**
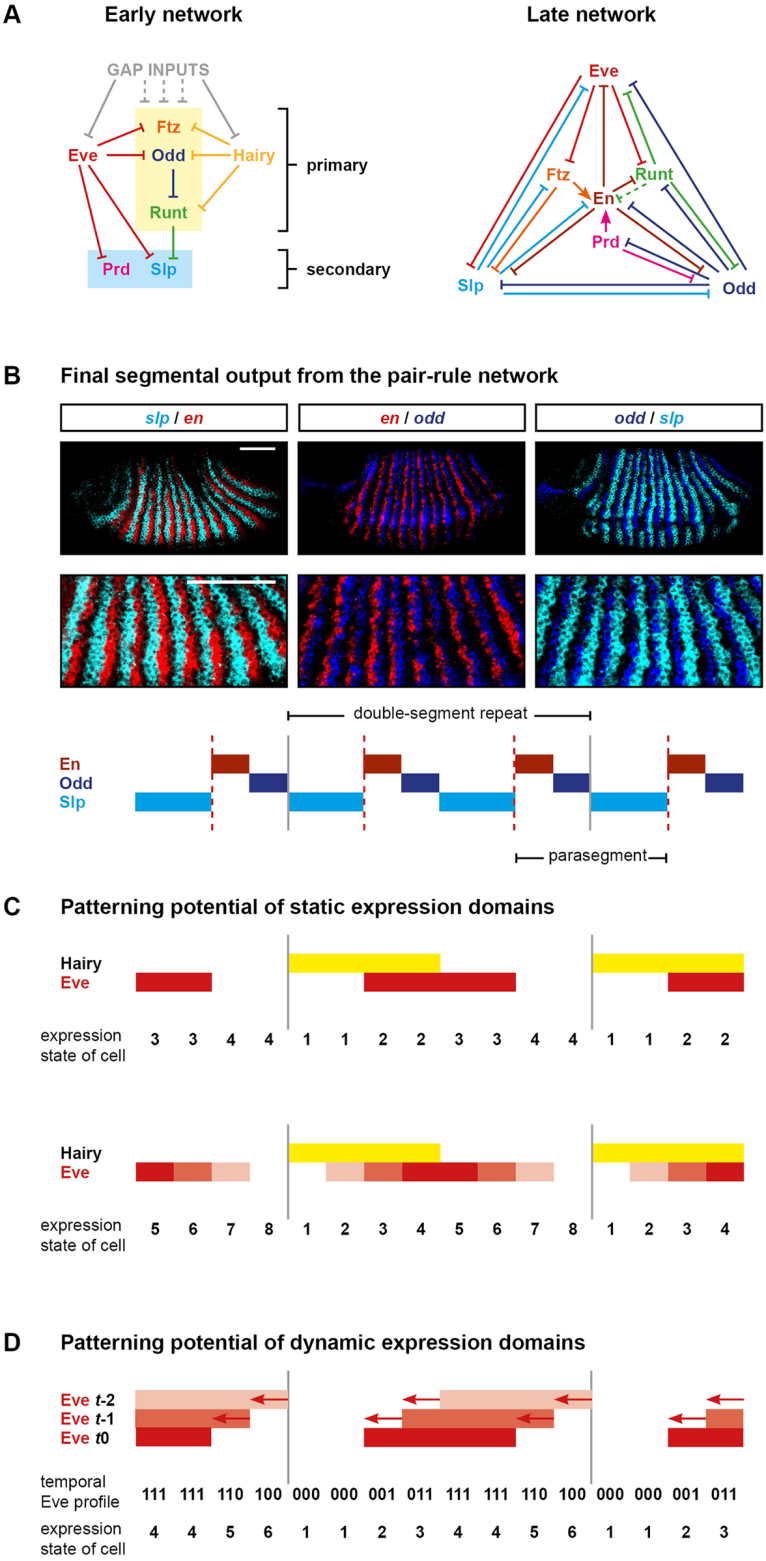
Structure and patterning function of the pair-rule gene regulatory network. (A) Cross-regulatory interactions between pair-rule genes during cellularisation (left) and gastrulation (right). Hammerhead arrows represent repression; pointed arrows represent activation. The zebra elements of *ftz*, *odd*, and *runt* (yellow box) turn on earlier than those of *prd* and *slp* (blue box). *ftz*, *odd*, and *runt* are also regulated by gap inputs through their stripe-specific elements (noted by grey dotted lines), but pair-rule inputs are the dominant influence on their patterns by mid-cellularisation (see S1 Text). The repression of En by Runt in the late network is dotted because, while the odd-numbered *en* stripes are sensitive to Runt, the even-numbered stripes are regulated differently [68]. Note that the regulation of *runt* in the late network reflects the regulatory logic of the “7-stripe” element rather than that of the “6-stripe” element [32,69]. (B) The template for polarised parasegment boundaries is formed by a repeating pattern of En, Odd, and Slp stripes. Top: whole mount double FISH images (anterior left, dorsal top) showing that *en*, *odd*, and *slp* are expressed in abutting, mutually exclusive domains. Middle: enlarged views of the stripes. Bottom: schematic of the overall pattern (anterior left). The grey vertical lines indicate the span of an initial pair-rule repeat relative to the final output pattern. Parasegment boundaries (dotted red lines) will form at the interface between En and Slp domains. Scale bars = 100μm. (C) Schematics indicating the number of distinct states that can be specified by static domains of Hairy and Eve expression. Top: Hairy and Eve are both Boolean variables. There are only 4 possible expression states (1: Hairy on, Eve off; 2: Hairy on, Eve on; 3: Hairy off, Eve on; 4: Hairy off, Eve off). Bottom: Hairy is still Boolean, but Eve is now a multilevel variable. Different shades of red represent different levels of Eve activity: low (lightest), medium, or high (darkest). There are now 8 different possible combined expression states of Hairy and Eve. Grey vertical lines are as in (B). (D) Rich positional information can be conveyed by a dynamic signal. Top: Boolean Eve stripes are depicted travelling from posterior to anterior over time (darker red represents a more recent time point). Middle: The Eve profile over time is recorded in binary digits. Bottom: The Eve signal can be decoded into 6 distinct expression domains, aligned with those in (B). Grey lines are as in (B). **Abbreviations**: *en*, *engrailed*; FISH, fluorescent in situ hybridization; *ftz*, *fushi tarazu*; *odd*, *odd-skipped*; *prd*, *paired*; *slp*, *sloppy-paired*.

Fig 1A summarises the inferred regulatory interactions between the pair-rule genes. Following Clark and Akam (2016) [32], individual interactions are assigned to distinct “early” and “late” networks, which operate during mid-cellularisation or late cellularisation/gastrulation, respectively. Note that a few regulatory interactions (e.g., repression of *ftz* by Eve) are common to both networks, but the majority are restricted to a single phase of patterning.

Gene regulatory network models represent “intellectual syntheses” of diverse experimental data [70]. I arrived at the topologies in Fig 1A by carefully analysing relative expression data in tightly staged wild-type embryos and cross-referencing these observations with the large number of mutant and misexpression experiments recorded in the genetic literature (for example, [8,30,31,64,71–78]). In places where the data were particularly ambiguous, I also re-characterised pair-rule gene expression in select pair-rule mutants in order to pick apart direct versus indirect regulatory interactions. In almost all cases, the interactions in the network diagrams have been previously inferred by multiple sets of researchers; my contribution has been (1) to bring this body of work together into something consistent and relatively complete and (2) to recognise the distinction between the early and late phases of regulation, rather than pooling all interactions into a single network. Most of the evidence and reasoning behind the inferred interactions (and interactions inferred to be absent) in Fig 1A are described in Appendix 1 of Clark and Akam (2016) [32]. Additional evidence in favour of the “early” cross-regulatory interactions between the primary pair-rule genes (boxed yellow area in Fig 1A, left) is presented in S1 Text, based on patterns of pair-rule gene expression in *hairy*, *eve*, and *runt* mutants.

Two things are immediately clear from the network diagrams. First, the direct regulatory interactions between the pair-rule genes are overwhelmingly repressive. This is consistent with a mode of patterning consisting of spatially ubiquitous activation (by maternally provided factors, for example) combined with precisely positioned repression from other segmentation genes [76,79–81]. While certain of the pair-rule factors (e.g., Ftz and Prd) have been shown to quantitatively up-regulate the expression of other pair-rule genes and thus contribute to this background activation in a spatially modulated way [82–84], these effects do not, for the most part, seem to be important for qualitatively determining the spatial pattern of pair-rule gene expression and so have been omitted from the diagram. Most described incidences of one pair-rule gene genetically activating another pair-rule gene are instead indirect (i.e., mediated by the direct repression of another repressor).

Second, the 2 networks have very different structures, presumably reflecting the different patterning function each must perform. During mid-cellularisation, pair-rule gene cross-regulation is responsible for refining many of the pair-rule stripes and standardising their phasing relative to other pair-rule stripes, resulting in a regular repeating pattern of double-segment periodicity. This is carried out by the early network, which is sparse, composed of unidirectional regulatory interactions, and has no feedback loops. Two of the pair-rule genes, *eve* and *hairy*, are patterned by gap factors rather than other pair-rule factors and so represent “input-only” factors to the network [16]. The remaining primary pair-rule genes (*runt*, *ftz*, and *odd*) do receive extensive gap inputs at early stages of cellularisation, but, by mid-cellularisation, their patterns are largely specified by other pair-rule genes (see S1 Text). (Note, however, that some aspects of the *ftz* pattern cannot be explained by pair-rule inputs alone, see Appendix 2 of [32].) The secondary pair-rule genes turn on later (*prd* at mid-cellularisation and *slp* towards the end of cellularisation) and are patterned by primary pair-rule genes. Overall, the early network has a hierarchical structure, in which Eve and Hairy convey positional information derived from the gap factors to the remaining primary pair-rule genes and eventually to the secondary pair-rule genes.

The late network, on the other hand, is extremely dense and consists largely of mutually repressive pairs of interactions. It is responsible for converting a double-segmental pattern of overlapping stripes into a segmental pattern of discrete segment-polarity fates. This is the final step in the *Drosophila* “segmentation cascade” and completes the transition from the analog (graded) positional information carried by the maternal and gap gene products to the essentially digital positional information carried by the segment-polarity genes [64]. The numerous positive (i.e., double-negative) feedback loops within the late network are consistent with it acting like a multi-stable switch, individual segment-polarity fates representing attractor states towards which the system will rapidly converge.

### Pair-rule patterning and positional information

As described above, gap inputs and the early pair-rule network combine to establish a repeating double-segmental pattern of pair-rule gene expression. The positional information within this pattern is then converted into a stable output pattern of segment-polarity states by the late network. Each initial double-segment repeat is about 7–8 nuclei wide, and each specified segment will consist of at least 3 distinct states characterised by the expression of *engrailed* (*en*), *odd*, and *slp*, respectively (Fig 1B). The *en* and *odd* stripes are about 1 nucleus wide, while the *slp* stripes are about 1–2 nuclei wide. Parasegment boundaries form wherever En and Slp domains abut, while Odd provides a buffer zone that preserves the AP polarity of each segment. (This tripartite segment pattern conforms to prescient theoretical predictions made by Hans Meinhardt in the early 1980s [62,85,86].) It is crucial that all 3 domains are specified within each segment—and that they are in the correct order—because patterning defects such as boundary losses, ectopic boundaries, and/or polarity reversals arise when the pattern is perturbed [8,31,33,76,87].

The extremely high resolution of the final segmental output pattern implies that the initial double segment pattern established by the early pair-rule network must contain sufficient positional information to allow almost every nucleus to be distinguished from its immediate neighbours. We are thus left with 2 questions. First, how does the early network establish a situation in which the different nuclei within a double-segment repeat each expresses a unique combination of pair-rule factors? Second, how exactly is this code “read” by the late network? (Or, in other words, which sets of initial conditions will result in a cell following an expression trajectory that ends at, for example, stable *en* expression, rather than stable *odd* or stable *slp*?)

In later sections, I address these questions by simulating and analysing the networks shown in Fig 1A. However, before getting into specifics of how particular genes are regulated and expressed, it is worth considering a more fundamental question: where is the positional information coming from in the first place? The topology of the early network (Fig 1A, left) implies that, to a first approximation, all the positional information in the final pattern must trace back to the expression patterns of just 2 factors, Eve and Hairy. Boolean (ON/OFF) combinations of Eve and Hairy would only be sufficient to specify 4 different domains within each double-segment repeat (Fig 1C, top), whereas the real output pattern consists of at least 6 distinct domains (i.e., En, Odd, Slp, En, Odd, Slp). How is it possible that just 2 independent spatial signals are able to give rise to such a high-resolution final output?

One potential answer is that stripes of Eve and/or Hairy might carry quantitative information that permits them to convey more than 2 “states” within the positional code. Since the early 1990s, this idea has been applied to the graded margins of the early Eve stripes [30,75,88]. These stripes have been proposed to act as local morphogen gradients, repressing different target genes at different concentration thresholds and thus differentially positioning their respective expression boundaries. Current models of pair-rule patterning rely on the assumption that there are 4 functionally distinct levels of Eve activity across an Eve stripe (from the centre to the edge: HIGH, MEDIUM, LOW, and OFF) [8,44]. These different levels would provide cellular-level resolution within each double-segment repeat and, combined with information from the Hairy stripe, allow each nucleus to be uniquely specified (Fig 1C, bottom).

While a given concentration of Eve protein may well repress its various targets with different efficacies, it is unlikely that segment patterning relies significantly upon this mechanism, for 3 main reasons. First, for the model to be viable, the Eve stripes would need to provide an accurate and precise set of positional signals within each double-parasegment repeat, i.e., the Eve stripes would have to be extremely regular and all share the same shape and amplitude. However, more posterior Eve stripes show significantly lower expression levels than more anterior Eve stripes throughout most of cellularisation [59]. Furthermore, pair-rule transcripts are apically localised, and therefore pair-rule gene expression becomes effectively cell autonomous soon after membrane invagination begins [89,90]. This means that, unlike for the gap genes (whose transcripts remain free to diffuse between neighbouring nuclei), for *eve*, there is little or no spatial averaging to buffer the high intrinsic noise of transcription [91,92]. This reduces the precision of the Eve signal and thus its capacity to reliably convey analog information.

Second, the morphogen model also requires the readout of the Eve signal to be very sensitive; i.e., *eve* target genes would have to reliably discriminate between different Eve expression levels and pattern their expression boundaries accordingly. However, it is not clear that this actually occurs within the embryo—for example, the model proposes that graded Eve stripes result in offset boundaries of the Eve targets *odd* and *ftz*, but recent observations indicate that these offsets are in fact produced by other mechanisms [32].

Third, the morphogen model does not explain the full severity of the *eve* null mutant phenotype, in which aberrant expression patterns are seen even in regions that would be outside the Eve stripes in wild-type embryos. Neither does the morphogen model explain the patterning robustness of *eve* heterozygotes, in which halving Eve expression levels fails to perturb the overall pattern of segment-polarity domains.

How, then, might the spatial resolution of the segment pattern be explained if not by an Eve morphogen gradient? Traditional models of *Drosophila* segmentation are essentially static: each tier of segmentation gene expression provides a single set of spatial signals, which is transduced into a new set of spatial signals by the tier below. This simplifies the real situation in the embryo, in which both gap and pair-rule expression domains shift subtly from posterior to anterior over time [59,93]. Explicitly considering these temporal aspects of segmentation gene expression suggests an alternative segment patterning mechanism: using the temporal dynamics of a relatively coarse pair-rule signal to provide high-resolution spatial information across each pattern repeat.

A signal that varies over time can be used to convey an arbitrary quantity of information, even if each reading of that signal provides very little (think of Morse code or binary storage). The *eve* and *hairy* stripes continue to be regulated by gap inputs throughout most of cellularisation and therefore shift across nuclei in concert with the gap domains. This means that, rather than each nucleus having to deduce its position from a single level of, e.g., Eve protein (as in the morphogen model), the nucleus actually experiences a temporal sequence of Eve protein levels. Strikingly, an overall shift of just 2 nuclei would be theoretically sufficient for a Boolean Eve stripe to, on its own, specify the positions of all 6 segment-polarity domains within a double-segment repeat (Fig 1D).

This kind of mechanism would, however, rely on the downstream targets of Eve and Hairy being able to decode a temporal sequence of Eve/Hairy expression and convert it into an appropriate segment-polarity fate. In the following sections, I carry out simulations and analysis of the network shown in Fig 1A and, based on the results, argue that the cross-regulatory interactions between the pair-rule genes function to achieve exactly this task.

### A simple model of the pair-rule system

In order to investigate how pair-rule patterning works, I used the networks shown in Fig 1A to create a toy model of the pair-rule system and then simulated pair-rule gene expression across an idealised 1-dimensional tissue. In this section, I briefly describe the structure and assumptions of the modelling approach; a full description of the model plus details of all simulations are given in S2 Text. (Source code for running the simulations is available in S1 and S2 Files, while pair-rule networks in SBML-qual format are available in S3 and S4 Files).

The genes whose regulation I model explicitly are the 7 pair-rule genes, plus *en*, whose product plays a key role in regulating late pair-rule gene expression. I have also included 4 inputs that are extrinsic to the system: 2 temporal signals, Caudal (Cad) [94] and Opa, and 2 signals to represent the positional information provided by the gap system, “G1” and “G2”. Cad represses the secondary pair-rule genes during early stages of patterning [87], while Opa turns on midway through patterning and triggers the switch from the early network to the late network [32]. G1 is responsible for patterning the *hairy* pair-rule stripes while G2 is responsible for patterning the *eve* pair-rule stripes. G1 and G2 do not represent specific gap factors but are instead an abstraction of the spatial inputs (i.e., stripe boundary locations) provided by the gap system as a whole.

Each gene in the system is represented by a Boolean variable, and its control logic is formalised using logical rules (essentially equivalent to the “logical equations” used in Sanchez and Thieffry [2003] [44] or the “vector equations” used in Peter et al. [2012] [70]). For example, if Opa is OFF (early network), *odd* is expressed only if both Hairy and Eve are also OFF, while if Opa is ON (late network), *odd* expression relies on all of Runt, En, and Slp being OFF (compare Fig 1A). In most cases (apart from, e.g., activation of *en* by Ftz or Prd), gene activation is assumed to be driven by some ubiquitous background factor(s) and is not explicitly included in the model.

The network simulation proceeds by discrete time steps, with expression output at time point *t* + 1 calculated from the state of the system at time point *t*. Because of the speed and dynamicity of segment patterning, time delays associated with protein synthesis and protein decay imply that protein and transcript expression domains for a given gene will often be non-congruent within the *Drosophila* embryo. This is likely to be significant for patterning, and I therefore approximate this effect by adding simple time delay rules into the simulation. Each gene has associated “synthesis delay” and “decay delay” parameter values *s* and *d*, both of which are integers representing a certain number of time steps. (Once a gene turns on, transcript will be present immediately, but protein will only appear *s* time steps later. Similarly, once a gene turns off, transcript will disappear immediately, but protein will only disappear after another *d* timesteps have elapsed.) For parsimony (and consistent with real expression kinetics [82]) all pair-rule genes and *en* are assigned the same delay value, which applies to both *s* and *d*. The specific value of this delay is fairly arbitrary, because the ratio between different delays is what affects how the system behaves, but I have chosen this value to be 6. Given that the half-life of *ftz* RNA during cellularisation is 7 minutes [79], this means that each time step in the simulation can be thought of as representing on the order of 1 minute of real developmental time. The time delays of the other components in the network (Cad, Opa, G1, and G2) are assigned appropriate values relative to this timescale, so that their simulated behaviour roughly approximates their spatiotemporal expression in a real embryo.

The simulation is set up to occur across a row of 20 “cells”, an idealised representation of the AP axis. This row of cells is not meant to correspond to a specific region of the *Drosophila* embryo but rather to be generally representative of patterning within the main trunk (i.e., pair-rule stripes 3–6), in which pair-rule genes are not additionally affected by cephalic or terminal factors. Each cell within this “tissue” is simulated independently, starting from a specific set of initial conditions. (As mentioned above, pair-rule transcripts are apically localised, and therefore the cross-regulation between the pair-rule genes is likely to be effectively cell autonomous from roughly mid-cellularisation onwards.)

The starting conditions for each cell usually involve specifying the appropriate expression of G1 and G2, setting Cad to ON, and setting all other genes to OFF. G1 and G2 are initialised with patterns that are offset by 2 cells and repeat every 8 cells, meaning that the *hairy* and *eve* stripes specified by these inputs will partially overlap and exhibit a double-segment periodicity, as in real embryos. Gap inputs into *runt*, *ftz*, and *odd* are omitted, meaning that their early expression is organised entirely by the spatial inputs from Hairy and Eve. As the simulation proceeds, Cad protein will disappear, allowing *prd* to turn on [87], followed by *slp*. (Note that we do not currently know how exactly the timing of *slp* expression is controlled, so in order to reproduce the timing observed in the embryo, *slp* expression in the simulation requires Prd expression to already be present.) Shortly afterwards, Opa protein will turn on, switching the control logic of pair-rule gene expression to the late network and causing pair-rule gene expression to eventually reach a final, stable state. After the switch to the late network, the gap factors and Hairy cease to regulate the pair-rule genes and then fade away, as in real embryos.

Note that the model just described, which is Boolean, deterministic, and uses discrete time steps, is not designed to capture the full complexity of the embryo (in which gene expression is, of course, quantitative, stochastic, and continuous). Rather, it represents a tool to expose the key mechanisms of patterning—and to delineate how much of what is observed in the embryo follows simply from the qualitative structure of the regulatory network. It also provides an important sanity check of the inferences that led to that structure being proposed in the first place.

### Posterior-to-anterior expression shifts are crucial for pair-rule patterning

**Fig 2:**
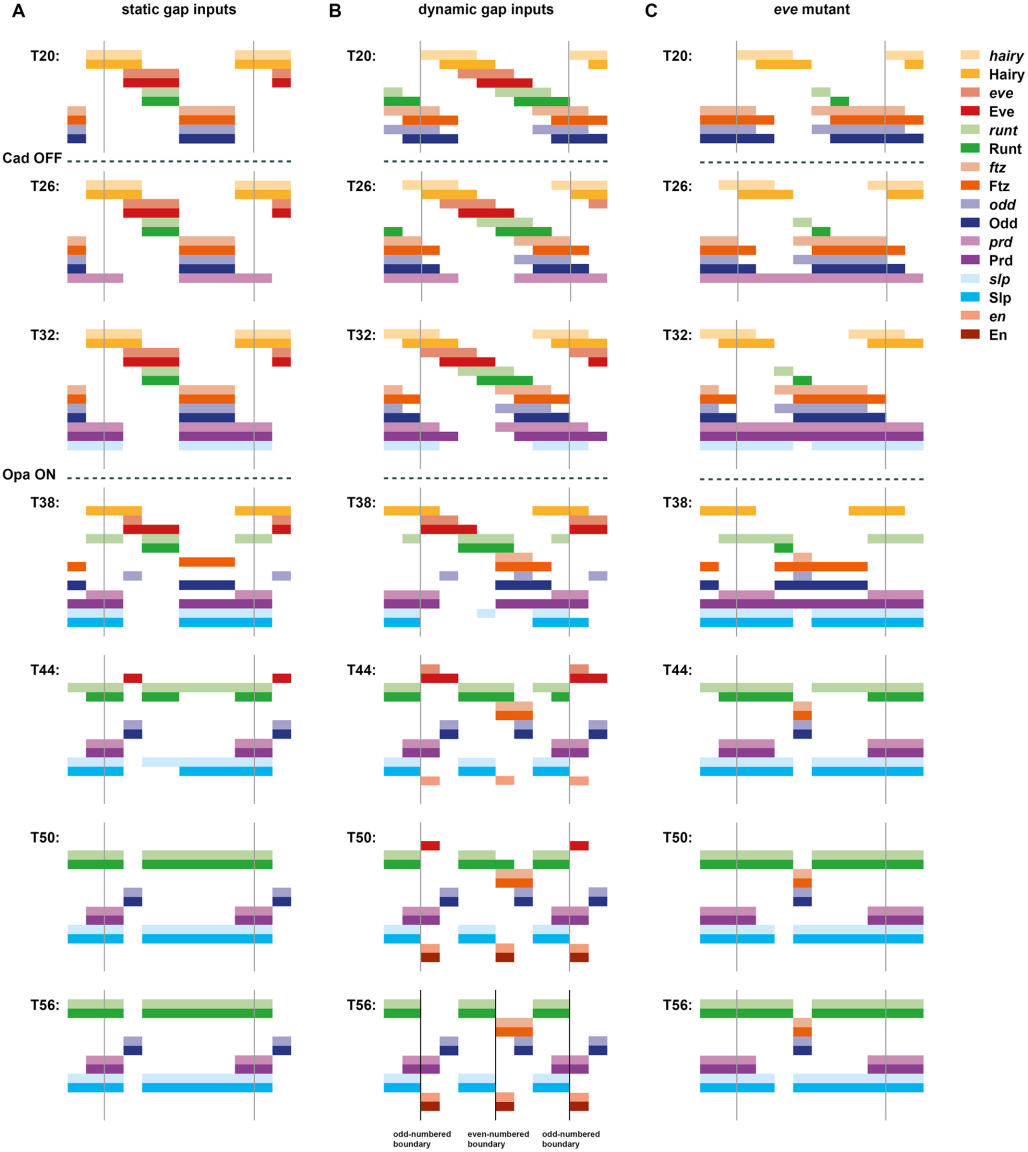
Simulation output from a Boolean model of the pair-rule network. Pair-rule gene expression patterns generated by simulating a Boolean model of the pair-rule network, assuming static “gap” inputs (A), dynamic “gap” inputs (B), or dynamic “gap” inputs and no Eve expression (C). Original simulations are shown in S9–S11 Movies. In each panel, the horizontal axis represents the anteroposterior (AP) axis (anterior left), while the vertical axis represents the different gene products that might be expressed in a given “cell” (column). Pale colours represent active transcription; dark colours represent active protein (see colour key at top right). Grey vertical lines indicate the span of an idealised double-segment repeat of 8 “cells”. The same 7 time points (T20–T56) are shown for each simulation. Cad turns off between the first (T20) and second (T26) panels (dashed lines), derepressing *prd* and *slp*. Opa turns on between the third (T32) and fourth (T38) panels (dashed lines), causing a switch from the early to the late network logic. Parasegment boundaries (black vertical lines at T56, located between abutting domains of Slp and En) are only produced by the simulation with dynamic gap inputs (B). **Abbreviations**: Cad, Caudal; Opa, Odd-paired; *prd*, *paired*; *slp*, *sloppy-paired*.

Using the model described above, I first simulated a scenario in which gap gene inputs and hence the pair-rule stripes of Hairy and Eve are completely static. The results are shown in S9 Movie and are summarised in Fig 2A. Under these conditions, the positional information provided by Hairy and Eve is essentially equivalent to the situation diagrammed in Fig 1C (bottom) and thus has no possibility of generating the correct segmental output. Unsurprisingly, the simulation does a bad job of recapitulating the patterns of pair-rule gene expression seen in real embryos (see below). In particular, at the end of the simulation, there is no *en* expression anywhere at all, and neither *odd* nor *slp* is expressed in a segmental pattern.

I then simulated a scenario in which the gap gene inputs and hence the Hairy and Eve stripes shift anteriorly over time. Given that the shift rate in real embryos is of the same order as the synthesis and decay rates of the segmentation gene products, I set the rate of these shifts to be such that the time taken for an expression domain to shift anteriorly by 1 cell is equal to the synthesis/decay delay parameter value of the pair-rule genes, i.e., 6 time steps (see S2 Text).

**Fig 3:**
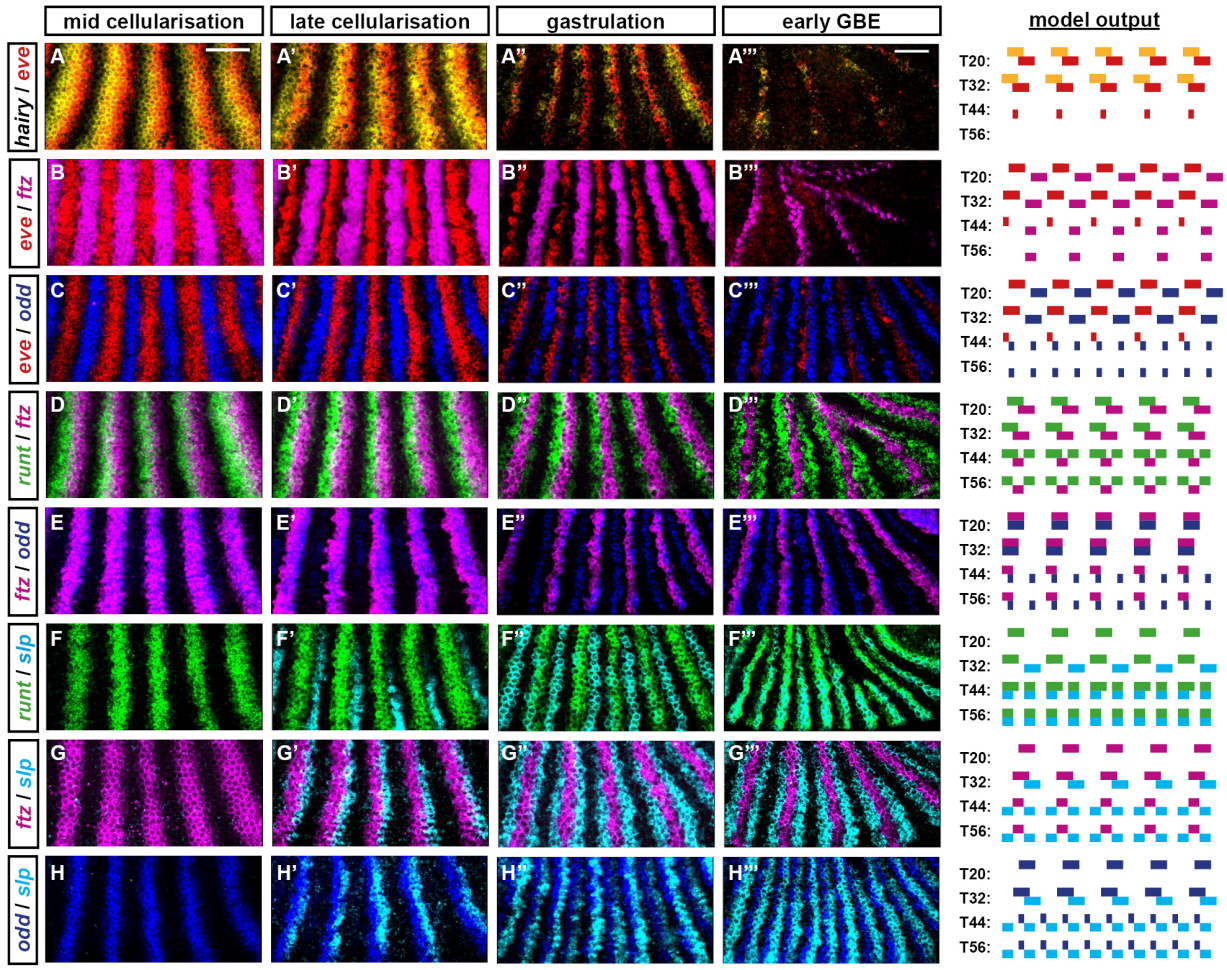
The “dynamic” simulation accurately recapitulates the spatiotemporal expression of the pair-rule genes. Left: false-coloured double FISH images for selected combinations of pair-rule gene transcripts at 4 different stages. Each panel shows a lateral view of stripes 2–6 (anterior left, dorsal top). Scale bars = 50 μm (rightmost panels use a slightly lower magnification due to tissue rearrangements). Additional expression combinations are shown in S1 Fig, while uncropped views of the embryos are shown in S2 Fig. Right: simulated transcriptional output for these pairs of genes at 4 different time points (see Fig 2B). The earliest time point of the simulated expression (T20) is representative of the leftmost panels of real expression (mid-cellularisation), and so on. The simulation output is generally very similar to the real expression patterns, with 2 main differences related to the discrete nature of the simulations. (1) Real gene expression domains fade over time rather than turning off instantaneously (e.g., compare *hairy* in A′–A″ to the simulated *hairy* expression at T32/T44/T56). (2) Qualitative expression pattern changes may occur gradually between late cellularisation and early gastrulation (e.g., *eve* expression in B–B″/C–C″ or *odd* expression in C–C″/E–E″) rather than instantaneously, as between T32 and T44. (A) *hairy* and *eve* pair-rule stripes partially overlap during cellularisation. At gastrulation, *hairy* expression fades away, while the *eve* stripes narrow from the posterior and then also fade. (B) *eve* and *ftz* pair-rule stripes are at first expressed in complementary patterns. Starting from late cellularisation, they both narrow from the posterior (*eve* more than *ftz*). *eve* expression later fades away, while *ftz* persists. (C) *eve* and *odd* pair-rule stripes are at first expressed in complementary patterns before both narrowing. *odd* secondary stripes emerge at the posterior of the narrowing *eve* domains, which then fade away, leaving segmental stripes of *odd*. (D) *runt* and *ftz* pair-rule stripes partially overlap throughout cellularisation. At gastrulation, *runt* secondary stripes emerge to the posterior of the narrowing *ftz* stripes. Later, the *runt* primary stripes refine from the posterior, and the overlaps with *ftz* are lost. (E) The *ftz* and *odd* stripes are fairly congruent during cellularisation. At gastrulation, both narrow from the posterior, and the *odd* secondary stripes intercalate between them. Over the course of patterning, their anterior boundaries also become offset from one another. (F) The *slp* primary stripes emerge later than the *runt* primary stripes and are offset slightly from their posterior boundaries. (The simulated *slp* domains are wider than the real *slp* domains.) At gastrulation, secondary stripes of both genes emerge between the primary stripes (the widths of the simulated *slp* stripes are now appropriate). The expression patterns become largely congruent, except at the posteriors of the *runt* primary stripes. These differences resolve later, when the *runt* primary stripes narrow. (G) The *slp* primary stripes emerge later than the *ftz* primary stripes and partially overlap with them. At gastrulation, the secondary *slp* stripes emerge just anterior to the *ftz* domains, which narrow from the posterior, losing the overlaps with the *slp* primary stripes. (H) As for (G), the *slp* primary stripes partially overlap the *odd* primary stripes, and these overlaps are later lost by the *odd* stripes narrowing from the posterior. The secondary stripes of *odd* and *slp* intercalate between the primary stripes and abut one another. **Abbreviations**: *eve*, *even-skipped*; FISH, fluorescent in situ hybridization; *ftz*, *fushi tarazu*; *odd*, *odd-skipped*; *slp*, *sloppy-paired*.

The simulation output for this scenario is shown in S10 Movie and summarised in Fig 2B. Even though the pair-rule network is unchanged and the Hairy and Eve stripes retain the same pattern and relative phasing as for the static simulation, the model now performs completely differently. Qualitative aspects of actual pair-rule gene expression (i.e., whether the expression domains of each pair of genes are congruent, overlapping, abutting, or separate, and the way this changes over time) are recapitulated remarkably well. For all pair-rule genes except *prd*, the match between the model output and the real spatiotemporal dynamics of gene expression is about as close as could be achieved by a simple, Boolean model—a few examples are highlighted in Fig 3, and the full set of comparisons is shown in S1 Fig. For *prd*, the real spatiotemporal expression profile is only partially recovered: the early pair-rule stripes are positioned correctly but do not refine correctly at later stages—they narrow rather than split, meaning that alternate segmental stripes are missing from the final pattern (S3 Fig). However, the *prd* domains missing from the simulation are not actually required for segment boundary patterning in real embryos (they are not reflected in the larval cuticles of *prd* mutants, although they do have minor effects on *wg* expression [6,31,95]). Accordingly, the simulation still generates the correct final segmental output: a repeating pattern of En, Odd, Slp x2, En, Odd, Slp x2.

These results tell us a number of things. First, it is not strictly necessary to invoke morphogen gradients in order to account for *Drosophila* pair-rule patterning. Second, posterior-to-anterior shifts of the Hairy and/or Eve stripes appear to be crucial for properly patterning the other pair-rule genes, and analysing the different behaviour of the static and shifting simulations should reveal exactly why. Third, the model as formulated is too simple to explain important aspects of the *prd* expression profile. Additional complexities that influence *prd* expression in real embryos could include (1) additional spatial or temporal regulatory inputs missing from the model, (2) quantitative information from existing spatial or temporal inputs that is not captured by the use of Boolean variables, or (3) differential synthesis/degradation rates of particular segmentation gene products not accounted for by the equal time delays assumed by the model. At least the first option seems to apply, as I have recently discovered that the Sox transcription factor Dichaete [96,97] also affects *prd* regulation [87].

### How and why do the shifts affect patterning?

#### Dynamic patterning of the early pair-rule stripes

**Fig 4:**
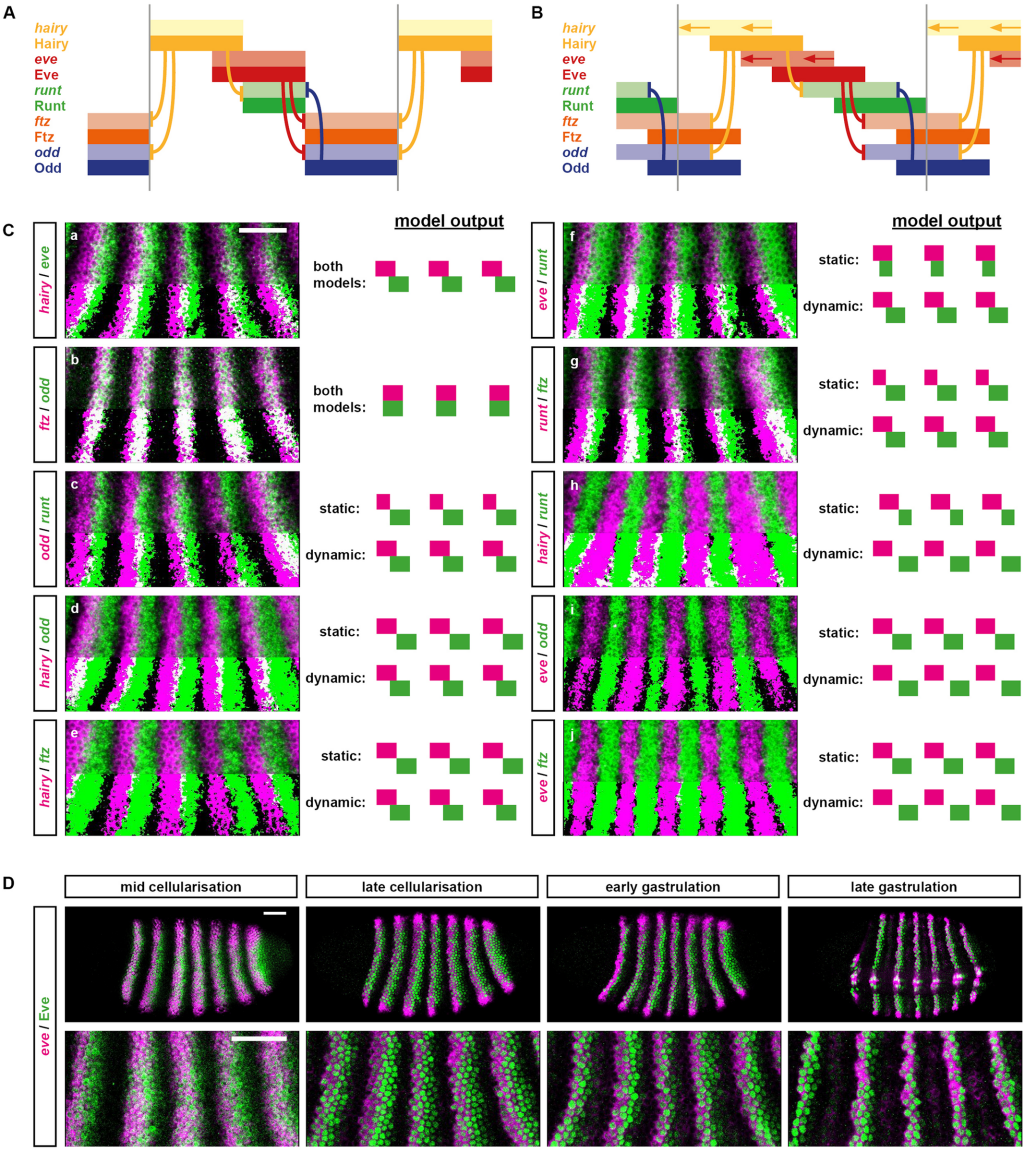
Dynamic patterning of the primary pair-rule genes. (A) Regulatory schematic showing the predicted phasing of the primary pair-rule stripes during cellularisation, assuming static, partially overlapping domains of Hairy and Eve. Pale colours represent transcript domains; intense colours represent protein domains; hammerhead arrows represent repressive interactions; grey vertical lines indicate the span of a double-segment pattern repeat. Cross-regulatory interactions are from the early network (compare Fig 1A, left). For simulation output, see S1 Movie. (B) Regulatory schematic showing the predicted phasing of the pair-rule stripes during cellularisation, assuming dynamic, partially overlapping domains of Hairy and Eve. *hairy* and *eve* domains shift anteriorly over time, resulting in offsets between transcript and protein domains. Colours, etc., as for (A). For simulation output, see S2 Movie. (C) Comparisons between real and predicted phasings of the primary pair-rule stripes. Double FISH images show lateral views of stripes 2–6 (anterior left, dorsal top) in mid-cellularisation stage embryos. In the bottom half of each image, the 2 channels have been thresholded, making regions of overlap easier to see. Scale = 50 μm. Diagrams to the right of each image show the stripe phasings predicted by static (top) or shifting (bottom) gap inputs, respectively (compare A and B). For panels (A) and (B), the 2 models predict the same relative pattern. In all other panels, the models predict different relative patterns. (D) Simultaneous visualisation of *eve* transcript (magenta) and Eve protein (green) in embryos at 4 different stages. Rightmost panels show a ventrolateral view; all other panels show lateral views. Upper panels show whole embryo views; lower panels show enlarged views of stripes 2–6. In stripes 3 onwards, the protein domains lag behind the transcript domains until late gastrulation, indicating that the anterior boundaries of the *eve* stripes shift anteriorly until early gastrulation. In stripe 2, the anterior boundary stabilises significantly earlier. Scale = 50 μm. **Abbreviations**: *eve*, *even-skipped*; FISH, fluorescent in situ hybridization.

First, let us consider the earliest phase of the simulations (time points 0–24, corresponding to mid-cellularisation in a real embryo), in which double-segmental stripes of the primary pair-rule genes *runt*, *ftz*, and *odd* are patterned by the early pair-rule network. Each of these genes is repressed by 2 other pair-rule factors: *ftz* and *odd* by Hairy and Eve, and *runt* by Hairy and Odd (Fig 1A, left). In the static simulation, this system rapidly reaches steady state (Fig 4A): *ftz* and *odd* are expressed in cells that express neither Eve nor Hairy, while *runt* is restricted to cells that don’t express Hairy but do express Eve (and hence don’t express Odd). This scenario is significantly different from the expression patterns of these genes in a real embryo, in which there are more overlaps between the various stripes (Fig 4C). For example, *runt* expression overlaps with *ftz* (Fig 4Cg) and *odd* (Fig 4Cc) as well as *eve*, while *ftz* and *odd* overlap slightly with *hairy* (Fig 4Cd and 4Ce).

These overlaps represent unstable combinations of gene expression (Hairy represses *ftz* and *odd* while Odd represses *runt*), but they are nevertheless present throughout the “early” phase of the second simulation, which incorporates stripe shifts. This is because the dynamic inputs from Hairy and Eve mean that primary pair-rule gene expression is not permitted to reach stable state (Fig 4B), and therefore a fraction of cells within the tissue will be in these transient states at any given point in time. Note that although the gene expression within each cell is constantly changing over the course of the simulation, the overall pattern of stripes remains constant once established, moving across the tissue like an elaborate Mexican wave.

Specifically, the additional overlaps seen in the second simulation (Fig 4B) arise because the combination of shifting dynamics with time delays for protein synthesis/decay means that domains of active transcription do not necessarily reflect domains of protein activity, and vice versa. If a pair-rule stripe is shifting anteriorly, the protein distribution of that stripe should lag behind the transcript distribution, a prediction I have verified by comparing *eve* transcript and Eve protein expression patterns within individual embryos (Fig 4D). After a gene turns on at the anterior edge of a stripe, there will be a delay before the protein appears and represses its targets, resulting in small transcriptional overlaps between repressors and their targets (i.e., anterior *hairy*/posterior *ftz*, anterior *hairy*/posterior *odd*, and anterior *odd*/posterior *runt*). Similarly, after a gene turns off at the posterior edge of a stripe, there will be a delay before the protein disappears and its targets are derepressed, resulting in small transcriptional gaps between repressors and their targets (i.e., posterior *eve*/anterior *odd*, posterior *hairy*/anterior *runt*, and posterior *eve*/anterior *ftz*). The exact size of the gaps and overlaps depends on the relationship between the speed of the expression shifts and the length of the time delays involved in protein synthesis and decay: faster relative shift rates will result in larger gaps and overlaps, while slower relative shift rates will result in a pattern closer to the static scenario (S6 Fig; S1–S5 Movies).

#### Dynamic patterning of the odd-numbered parasegment boundaries

**Fig 5:**
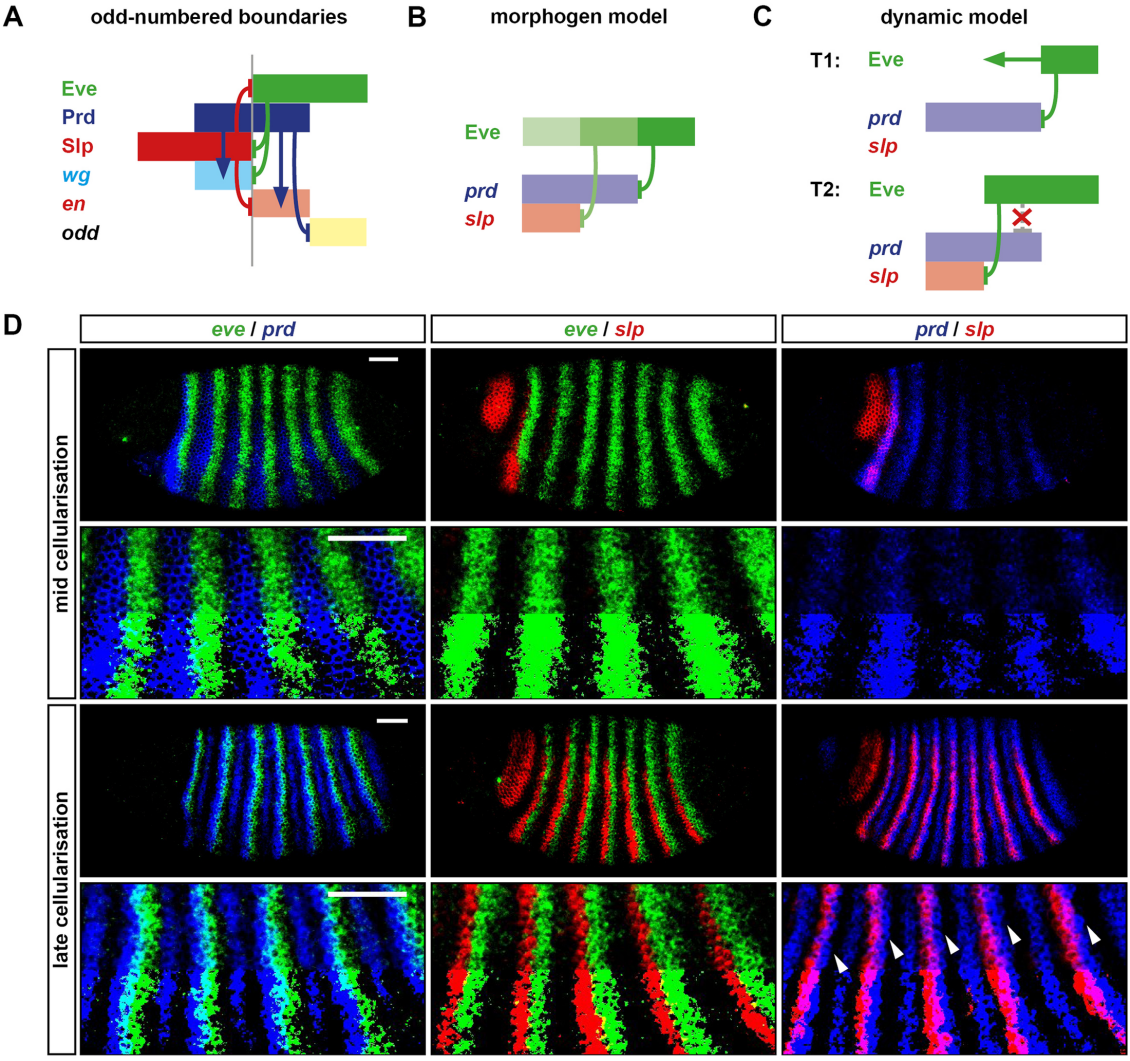
Dynamic patterning of the odd-numbered parasegment boundaries. (A) Regulatory schematic of gene expression at the odd-numbered parasegment boundaries. Domains of Slp, Prd, and Eve expression (dark colours) pattern segment-polarity stripes of *wg*, *en*, and *odd* (pale colours). Anterior left. Hammerhead arrows represent repressive interactions; pointed arrows represent activatory interactions; grey vertical line represents a prospective parasegment boundary. See S4 Fig for relevant in situ data. (B) Static, “morphogen gradient” model for the patterning of the *prd* and *slp* posterior borders by Eve. The anterior margin of the Eve stripe is graded, with higher levels of Eve protein (darker green) present more posteriorly. High Eve (dark green) is required to repress *prd*, but only medium Eve (medium green) is required to repress *slp*. Based on Fujioka et al. (1995) [30]. (C) Dynamic model for the patterning of the *prd* and *slp* posterior borders by Eve. *prd* is activated earlier (T1, mid-cellularisation) than *slp* (T2, late cellularisation). In between these time points, the anterior border of the Eve domain shifts anteriorly. The posterior border of the *prd* domain is patterned by Eve at T1, but the posterior border of the *slp* domain is patterned by Eve at T2, resulting in a more anterior location. *prd* is no longer repressed by Eve at T2, resulting in stable, overlapping expression of Eve and *prd*. (D) Double FISH images showing the relative phasing of *eve* (green), *prd* (blue), and *slp* (red) expression domains at mid-cellularisation and late cellularisation. Enlarged views of stripes 2–6 are shown below the whole embryo lateral views (anterior left, dorsal top). In the bottom half of each image, the 2 channels have been thresholded, making regions of overlap easier to see. At mid-cellularisation, *slp* is not expressed and the posterior borders of the *prd* stripes abut the anterior borders of the *eve* stripes. At late cellularisation, the posterior borders of the *prd* stripes overlap the anterior borders of *eve* stripes (note the regions that appear cyan), the posterior borders of the *slp* stripes sharply abut the anterior borders of the *eve* stripes, and the posterior borders of the *slp* stripes are offset anteriorly from the posterior borders of the *prd* stripes (arrowheads). These expression patterns are more consistent with the dynamic model (C) than the static morphogen model (B). Scale bars = 50 μm. **Abbreviations**: *en*, *engrailed*; *eve*, *even-skipped*; FISH, fluorescent in situ hybridization; *odd*, *odd-skipped*; *prd*, *paired*; *slp*, *sloppy-paired*; wg, wingless.

The next phase of the simulations (time points 25–36, corresponding to late cellularisation in a real embryo) is when the secondary pair-rule genes turn on in double-segment patterns and prepattern the odd-numbered (Prd-dependent) parasegment boundaries. These boundaries rely on setting up offset posterior boundaries of *prd* and *slp* expression: *en* is activated by Prd but repressed by Slp, thus resulting in narrow stripes of *en* expression posteriorly abutting each of the *slp* pair-rule stripes (Fig 5A; S4 Fig).

The posterior boundaries of *prd* and *slp* are patterned by Eve, which represses both genes during cellularisation (Fig 1A, left). While *slp* continues to be repressed by Eve throughout later development [88], *prd* becomes insensitive to Eve towards the end of cellularisation [30,32] for reasons that are not entirely clear but may relate to the loss of Dichaete expression from the blastoderm [87]. The current model for how the offsets are set up is that the anterior edge of each Eve stripe acts as a morphogen gradient, which represses *slp* at a lower Eve concentration than is required to repress *prd*, thus differentially positioning their expression boundaries (Fig 5B) [30]. The reason that patterning fails in the first simulation (Fig 2A) is that the static, Boolean stripes of Eve position the boundaries of both *prd* and *slp* at the same place, resulting in no offsets and hence no *en* expression. In the second simulation (Fig 2B), however, the *prd*/*slp* offsets do emerge, even though Eve expression remains Boolean. This is because *slp* expression turns on significantly later than *prd* expression (about 10 minutes later in real embryos [59]), by which time, the Eve boundary has shifted to a more anterior position than where it was when it patterned the *prd* stripes. Thus, whereas under the morphogen model, the distance between the *prd* and *slp* boundaries is measured using Eve concentration, an alternative model is that this distance is instead measured using time (Fig 5C).

These 2 mechanisms are not mutually exclusive, and it may be that they both contribute to patterning in the embryo. However, in their basic forms, they make different predictions about the relative expression dynamics of the *prd* and Eve domains. Under the static morphogen model, the 2 sets of domains should show significant overlaps throughout patterning (Fig 5B). Under the dynamic model, these overlaps should be absent when the *prd* stripes are first patterned and emerge later, once *prd* becomes resistant to Eve (Fig 5C). Real pair-rule gene expression in the embryo is more consistent with the dynamic model: the posterior boundaries of the *prd* stripes abut the anterior boundaries of the *eve* stripes when they first emerge, and it is only later, when *slp* starts to be expressed, that overlaps between *prd* and *eve* become obvious (Fig 5D).

#### Dynamic patterning of the even-numbered parasegment boundaries

**Fig 6:**
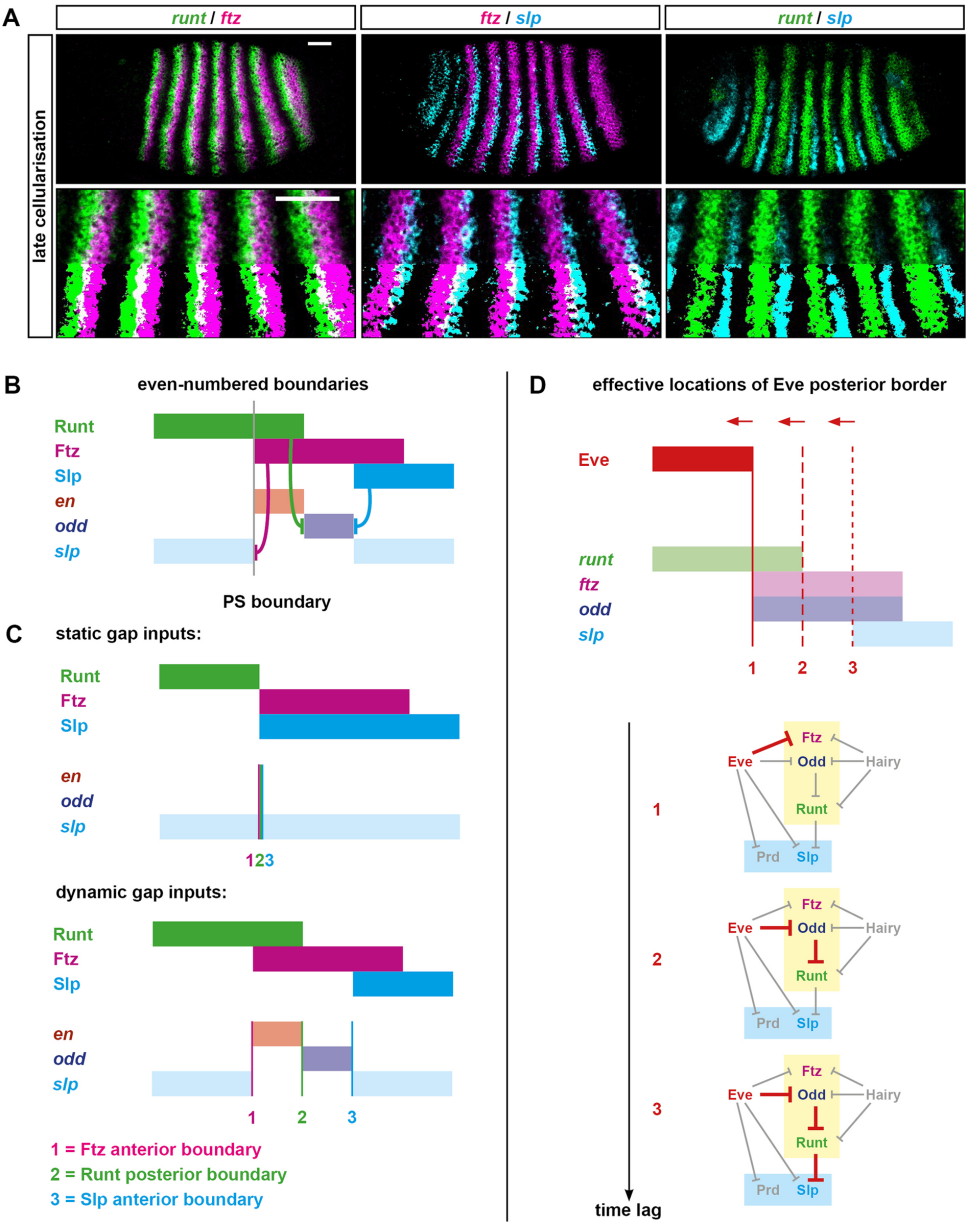
Dynamic patterning of the even-numbered parasegment boundaries. (A) Double FISH images showing the relative expression patterns of *runt*, *ftz*, and *slp* at late cellularisation. Enlarged views of stripes 2–6 are shown below whole embryo lateral views (anterior left, dorsal top). In the bottom half of each enlarged image, the 2 channels have been thresholded, making regions of overlap easier to see. Scale bars = 50 μm. (B) Regulatory schematic showing the patterning of the even-numbered parasegment boundaries. At gastrulation, Runt, Ftz, and Slp are expressed in partially overlapping domains similar to their transcript expression at late cellularisation (see A). These overlapping domains provide a template for the segment-polarity stripes of *en*, *odd*, and *slp*: the anterior borders of the Ftz stripes define the posterior borders of the *slp* secondary stripes, the posterior borders of the Runt stripes define the anterior borders of the *odd* primary stripes, and the Slp anterior borders define the posterior borders of the *odd* primary stripes. The even-numbered *en* stripes are activated by Ftz but repressed by Odd and Slp and so are restricted to the region of overlap between Runt and Ftz, in which both *odd* and *slp* are repressed [32]. Anterior left. Hammerhead arrows represent repressive interactions; grey vertical line represents a prospective parasegment boundary. (C) Schematic explaining why the even-numbered parasegment boundaries require dynamic gap inputs in order to be patterned. Given static inputs (top panel, compare Fig 2A), the Ftz anterior boundary (1, pink vertical line), the Runt posterior boundary (2, green vertical line), and the Slp anterior boundary (3, blue vertical line) all coincide, resulting only in broad *slp* expression. Given dynamic inputs (bottom panel, compare Fig 2B), the 3 boundaries are each located at different anteroposterior (AP) positions (as in B), resulting in the segment-polarity pattern: *slp*, *en*, *odd*, *slp*. (D) Schematic explaining the origin of the offset boundaries of *ftz*, *runt*, and *slp*. Top: diagram of the relative expression of Eve, *runt*, *ftz*, *odd*, and *slp* at late cellularisation (compare A, and see T32 in Fig 2B). The solid red vertical line indicates the current position of the Eve posterior border, which coincides with the *ftz* anterior border (1). Dotted red vertical lines indicate previous positions of the dynamic Eve posterior border, coinciding with the *runt* posterior border (2) or the *slp* anterior border (3). Bottom: the regulatory chains responsible for patterning each of the 3 expression boundaries are highlighted in red on the early pair-rule network. All 3 boundaries trace back to Eve, but more posterior boundaries correspond to longer regulatory chains and so incur a longer time lag to resolve, given a change in Eve expression. The 3 different genes (*ftz*, *runt*, and *slp*) are effectively patterned by increasingly earlier incarnations of the Eve stripes, and therefore the existence of spatial offsets between boundaries 1, 2, and 3 relies on the Eve posterior border shifting anteriorly over time. **Abbreviations**: *en*, *engrailed*; FISH, fluorescent in situ hybridization; *ftz*, *fushi tarazu*; *odd*, *odd-skipped*; *slp*, *sloppy-paired*.

The final phase of the simulations (time points 37–60, corresponding to gastrulation in real embryos) is when the system switches over to the late network, and the even-numbered (Ftz-dependent) parasegment boundaries are patterned. These boundaries are patterned by the pair-rule stripes of Runt, Ftz, and Slp, which together pattern segmental stripes of *en*, *odd*, and *slp* (Fig 6A and 6B) [32]. Correctly patterning these latter domains therefore relies on the early network being able to set up the correct relative pattern of *runt*, *ftz*, and *slp* expression. Crucially, this pattern includes narrow overlaps between *runt* and *ftz* (which will later give rise to *en* expression) and narrow gaps between *runt* and *slp* (which will later allow *odd* expression to be maintained). This means that the 3 relevant sets of expression boundaries (*ftz* anterior boundaries, *runt* posterior boundaries, and *slp* anterior boundaries) each need to fall in different positions.

The patterning of each of these sets of boundaries eventually traces back to the posteriors of the Eve stripes (see Fig 4B). *ftz* expression is directly patterned by Eve; *runt* is patterned by Odd, which is itself patterned by Eve; and *slp* is patterned by Runt, which, as just mentioned, is indirectly downstream of Eve. In the first simulation (Fig 6C, top), these Eve boundaries are static, and thus the boundaries of Runt, Ftz, and Slp end up coinciding exactly. This explains why the *en* stripes fail to emerge and *odd* expression is lost: the expression states that give rise to them are missing from the positional code.

However, the correct boundary positions do emerge from the second simulation (Fig 6C, bottom), even though the network is unchanged. Again, this result is caused by the dynamic nature of the Eve stripes. Each of the 3 boundaries is patterned by a regulatory chain of a different length (1, 2, or 3 interactions, respectively) and so will take different lengths of time to adjust to a given change in Eve expression (Fig 6D). This means that when the Eve stripes shift anteriorly over time, the 3 sets of boundaries each lag behind the Eve domains by different distances, thereby providing the necessary positional framework for the final segment pattern.

### Patterning dynamics explain the severity of the *eve* mutant phenotype

Above, I described how the final segmental output consists of the pattern [En, Odd, Slp, En, Odd, Slp] across each double-parasegment repeat. I then used a dynamical model of the pair-rule system to show how the Eve stripes are directly or indirectly responsible for patterning most of the expression boundaries in this pattern, including both sets of parasegment boundaries. In this section, I use the same model to simulate and dissect the *eve* mutant phenotype, which has proved hard to account for using traditional patterning models.

**Fig 7:**
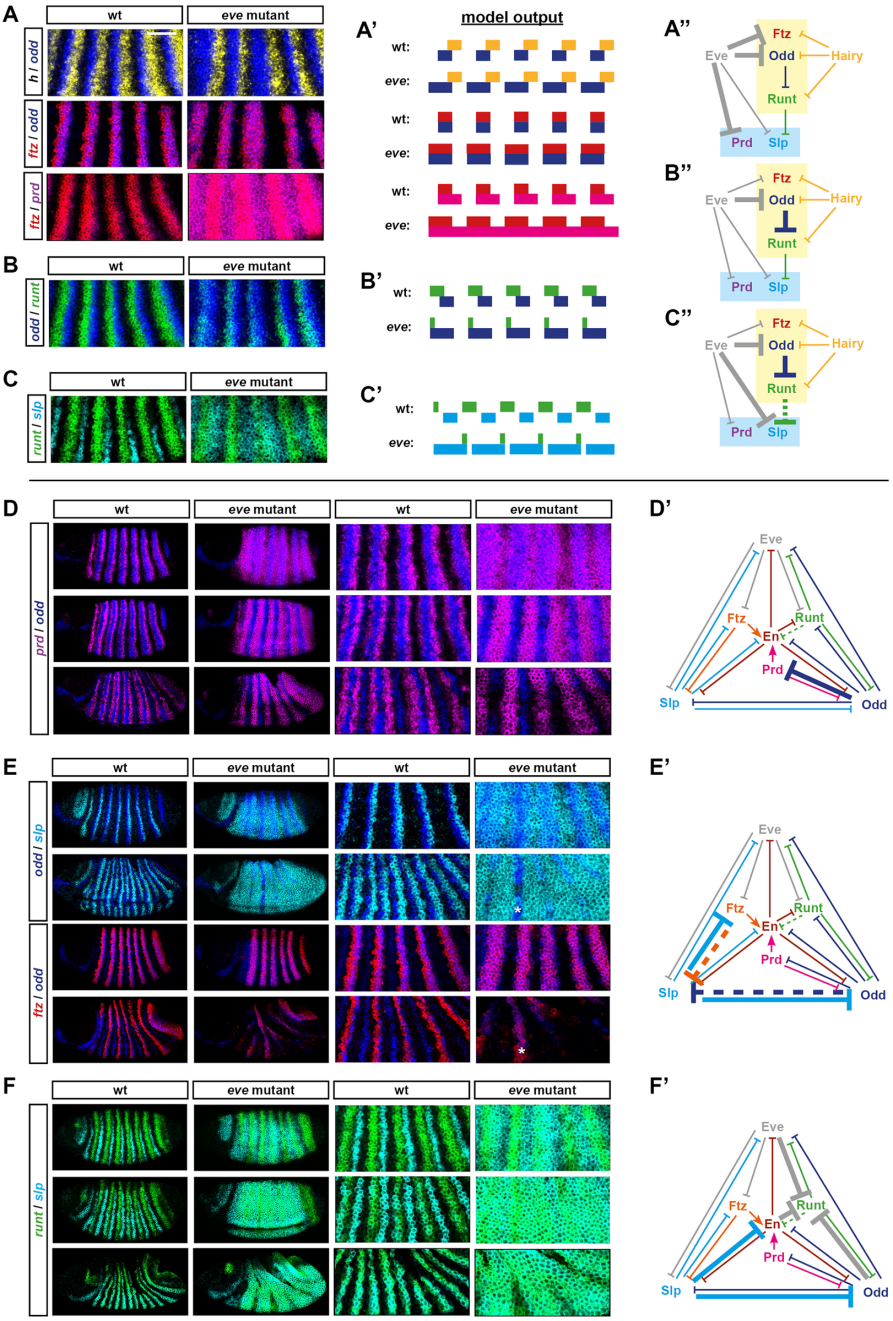
Aetiology of the *eve* mutant phenotype. (A–C) “Early” effects. (A–C) Double FISH images of pair-rule gene expression in cellularisation stage wild-type and *eve* mutant embryos. Enlarged views of stripes 2–6 are shown (anterior left, dorsal top). Scale = 50 μm. For whole embryo views and single channel views, see S5 Fig. (A′–C′) Predicted transcriptional output of these genes from “wild-type” and “*eve* mutant” simulations (compare T32 in Fig 2B and 2C). (A″–C″) Regulatory interactions relevant to the aberrant expression patterns in *eve* mutants are highlighted on the early pair-rule network (bold arrows). Eve and its regulatory effects, which are absent from the mutant embryos, are shown in grey. (A) Eve normally represses *ftz*, *odd*, and *prd*. In *eve* mutant embryos, all 3 genes are ectopically expressed: the *ftz* and *odd* stripes expand anteriorly, and *prd* is expressed ubiquitously rather than in stripes. These expression changes are recapitulated by the simulation. (B) Eve normally indirectly regulates *runt* expression by repressing its repressor, Odd. In *eve* mutant embryos, *odd* expression expands anteriorly (see A), resulting in a down-regulation of the *runt* stripes, except at their anterior margins (S5 Fig). This effect is recapitulated in a discrete manner by the simulation. (C) Eve normally regulates *slp* in 2 ways: (1) by repressing it directly, and (2) by repressing it indirectly via indirectly maintaining the expression of its repressor, Runt (see B), via direct repression of *odd* (see A). In *eve* mutant embryos, *slp* is expressed fairly ubiquitously rather than in narrow stripes. This expansion is evident in the simulated expression, but see legend of S5 Fig for discussion of differences between the real and simulated patterns. (D–F) “Late” effects. Double FISH images of pair-rule gene expression in wild-type and *eve* mutant embryos over the course of gastrulation. For each set of images, each row compares a wild-type and a mutant embryo of roughly equal age (age increases from top to bottom). Both whole embryo views (anterior left, dorsal top) and enlarged views of stripes 2–6 are shown. For single channel views, see S5 Fig. (D′–F′) Regulatory interactions that explain the observed pattern maturation are highlighted on the late network (bold arrows). (D) Odd represses *prd* in the late network, and so *prd* expression is lost from cells in which *odd* and *prd* expression initially overlap. In wild-type embryos, the *odd* primary stripes overlap the centres of the *prd* pair-rule stripes, which therefore split in two. In *eve* mutant embryos, broad *odd* stripes are overlain on initially aperiodic *prd* expression, which therefore resolves into a pair-rule pattern. (E) There is mutual repression between Slp and Ftz/Odd in the late network (E′). (Note that the repression from Slp appears to be stronger than the reciprocal repression from Ftz and Odd.) In wild-type embryos, Slp causes the primary stripes of both *odd* and *ftz* to narrow from the posterior (where they overlap the *slp* primary stripes). In *eve* mutant embryos, Slp is broadly expressed, causing general repression of *odd* and *ftz*. Note that expression of both *odd* and *ftz* persists in stripe 3 (asterisks), in which there is a corresponding gap in the *slp* expression domain. (F) In the late network, *slp* and *runt* are regulated similarly, and Slp represses all of the repressors of *runt* (i.e., *eve*, *odd*, and *en*). Consequently, *runt* and *slp* take on almost identical expression patterns. In wild-type embryos, the 2 genes become expressed in coincident segmental stripes. In *eve* mutant embryos, early broad expression of *slp* allows *runt* to also become ubiquitously expressed. Note that the *slp* domain later resolves into a pair-rule pattern (perhaps due to repression from residual Ftz protein). **Abbreviations**: *en*, *engrailed*; *eve*, *even-skipped*; FISH, fluorescent in situ hybridization; *ftz*, *fushi tarazu*; *odd*, *odd-skipped*; *prd*, *paired*; *slp*, *sloppy-paired*.

Although *eve* was originally identified as a pair-rule gene on the basis of a pair-rule cuticle phenotype [3], it turned out that this particular mutant allele was an *eve* hypomorph, while *eve* null mutants yield an aperiodic denticle lawn phenotype instead [98]. Both odd-numbered and even-numbered *en* stripes are absent from *eve* null mutant embryos [71], indicating severe mispatterning of upstream pair-rule gene expression. To investigate the aetiology of these effects, I characterised pair-rule gene expression patterns in precisely staged *eve* mutant embryos using double fluorescent in situ hybridisation (FISH) (Fig 7) and then cross-referenced these observations with the patterning output of an “in silico” *eve* mutant (Fig 2C; S11 Movie), simulated by starting with the dynamic model and then setting *eve* transcription to remain off (see S2 Text).

The experimental results are in accordance with earlier, more fragmentary characterisations of *eve* mutants [30,64,73,74,99–101] and reveal a number of significant changes to pair-rule gene expression patterns. The *odd* and *ftz* primary stripes are broader than usual (Fig 7A), and the early expression of *prd* and *slp* is largely aperiodic rather than pair rule (Fig 7A and 7C). Then, at gastrulation, segmental patterns of pair-rule gene expression fail to emerge; instead, *prd* resolves into broad pair-rule stripes (Fig 7D), *runt* and *slp* become expressed fairly ubiquitously (Fig 7F), and *odd* and *ftz* expression largely disappears (Fig 7E). These changes are also seen in the *eve* mutant simulation (Fig 2C) and, as described below, follow logically from the structure of the pair-rule network.

Because Eve expression plays a relatively minor role in late patterning, most of the expression changes just described result from the loss of Eve activity during cellularisation (time points 0–36 in Fig 2C and S11 Movie). First, *odd*, *ftz*, and *prd*, all of which are direct targets of Eve repression (Fig 7A′), are expressed ectopically: the *odd* and *ftz* primary stripes expand anteriorly (judged relative to *hairy*, Fig 7A, top/middle rows), while the *prd* interstripes (i.e., the gaps between the early broad stripes) are derepressed (Fig 7A, bottom row). The ectopic Odd expression has a knock-on effect on *runt* expression, which becomes down-regulated (Fig 7B and 7B′). (Note that while *runt* expression is almost entirely lost at this stage of the simulation, in real embryos *runt* remains fairly widely expressed, although at significantly lower levels—see S5 Fig. This difference may be partially due to expression from the *runt* stripe-specific elements, which are not included in the model.) The loss of Runt activity then contributes to the misexpression of the *slp* primary stripes, which turn on at the end of cellularisation and would normally be patterned by both Eve and Runt (Fig 7C′). Given the absence of Eve activity and the loss/weakening of Runt activity, *slp* becomes expressed almost ubiquitously within the trunk of the mutant embryos (Fig 7C).

Finally, the downstream effects of these aberrant patterns play out over the course of gastrulation, after the switch to the late network (time points 37–60 in Fig 2C / S11 Movie). Odd represses *prd*, causing the aperiodic domain of *prd* to resolve into a pair-rule pattern (Fig 7D and 7D′). Odd and Ftz also repress *slp*, causing some small gaps to appear in the *slp* pattern (Fig 7E and 7F). However, *odd* and *ftz* are themselves repressed by Slp very strongly (Fig 7E′), and the ectopic Slp expression in the embryo causes their expression to be almost completely lost (Fig 7E). In, contrast, new *runt* expression emerges throughout most of the trunk, due to the absence of its repressors, Eve and Odd (Fig 7F and 7F′).

As a consequence of all this mispatterning, *en* expression is completely repressed, and parasegment boundaries never form. The odd-numbered *en* stripes are specifically blocked by the ectopic Slp expression that replaces the Eve stripes. Above, we saw that these *en* domains are specified by the short regulatory chain [Eve––| Slp––| En], thus explaining why they have been observed to reappear in *eve*, *slp* double mutants [8,101]. On the other hand, the even-numbered *en* stripes are redundantly repressed in *eve* mutants, by both ectopic Odd and ectopic Slp, as a result of the regulatory chains [Eve––| Odd––| En] and [Eve––| Odd––| Runt––| Slp––| En], respectively. The model therefore explains why these stripes reappear in *eve*, *odd* double mutants [102] but not in *eve*, *slp* double mutants [8].

### The *Drosophila* pair-rule network is compatible with both simultaneous and sequential segmentation

By simulating the *Drosophila* pair-rule network, I have shown that dynamic spatial inputs from just 2 factors, Hairy and Eve, are sufficient to organise the expression of the system as a whole. In the *Drosophila* blastoderm, these inputs are driven by the dynamic output of the posterior gap system. However, the elaborate control of pair-rule gene expression by gap factors appears to be a relatively recent novelty in arthropod segment patterning, originating during the evolutionary transition from short-germ to long-germ embryogenesis [103,104]. It is currently not clear how much of the cross-regulation between the pair-rule genes seen in *Drosophila* is a new adaptation to long-germ development and how much is retained from a short-germ ancestral state.

**Fig 8:**
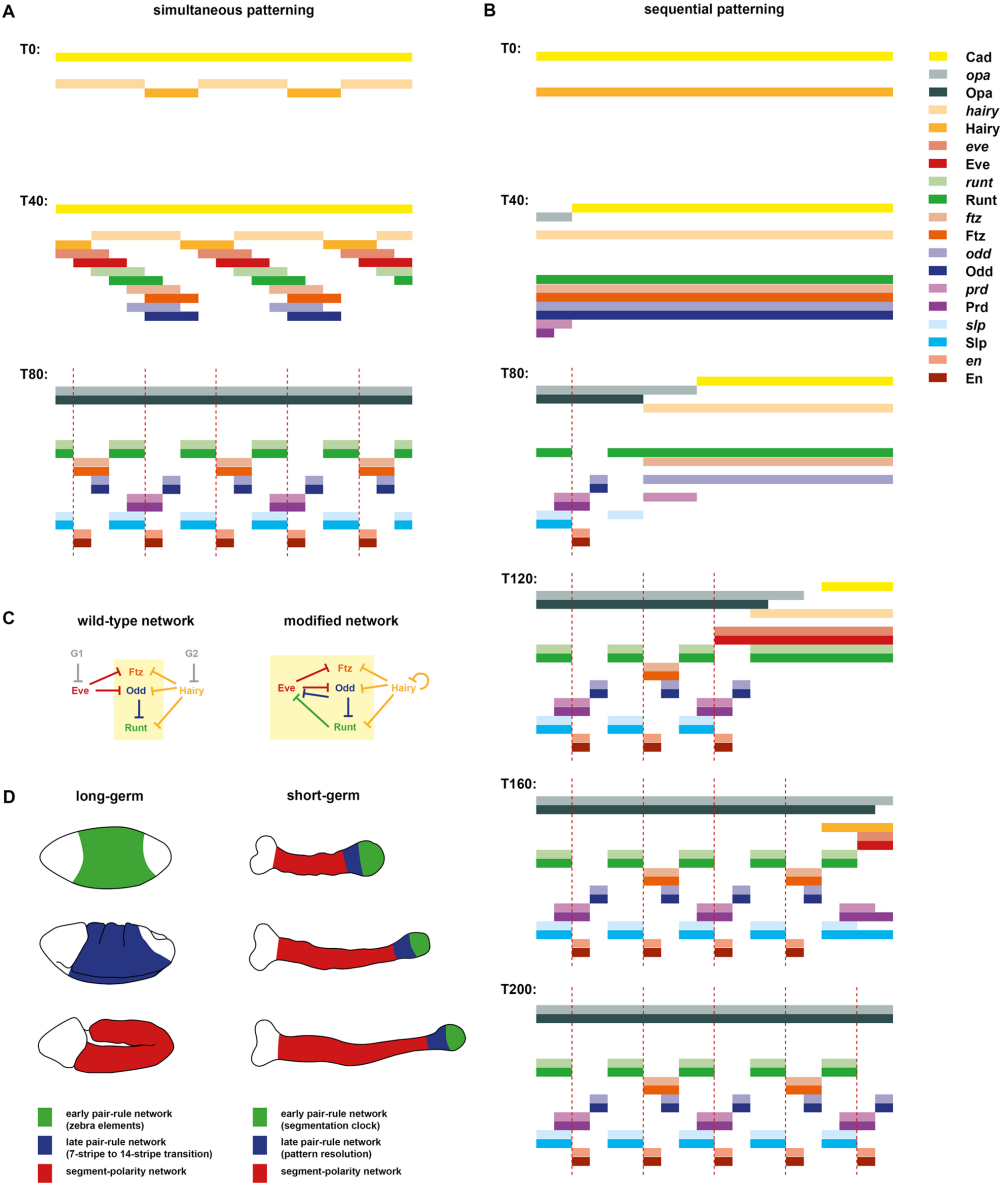
A slightly modified pair-rule network can pattern segments in both simultaneous and sequential modes. (A,B) Simulation output for the modified network. Each panel shows the system state at a specific time point between T0 and T200 (see S12 and S13 Movies for complete output). In each panel, the horizontal axis represents a region of the anteroposterior (AP) axis (anterior left) and the vertical axis represents the different gene products that might be expressed in a given “cell” (individual columns C1 to C20). Pale colours represent active transcription; dark colours represent protein activity (see colour key at top right). Dotted red lines indicate parasegment boundaries. (A) The system is initialised with uniform expression of Cad and a periodic phase gradient of Hairy expression (protein or transcript “age” increases from posterior to anterior, and the pattern repeats every 8 cells—see S2 Text for details). Given these starting conditions, the dynamical behaviour of the system is almost identical to the unmodified network (compare Fig 2B or S10 Movie). Note, however, that *hairy* transcript is always out of phase with Hairy protein. (B) The system is initialised with uniform expression of Hairy but a decay gradient of Cad (protein disappears from anterior to posterior over time). Given these altered starting conditions, the system behaves differently and patterning takes longer, but the same stable segmental pattern eventually emerges. Note that in this simulation, the primary pair-rule genes continuously oscillate within cells that do not express Opa, and segmental stripes emerge progressively from anterior to posterior as Opa turns on along the AP axis. (C) Comparison of the original (left) and modified (right) early networks. For simplicity, the secondary pair-rule genes are not shown. See text for details. (D) Proposed regulatory homology between phases of segmentation gene expression in long-germ embryos (left) and short-germ embryos (right). In *Drosophila*, the quintessential long-germ insect, there are 3 main phases of segment patterning during embryogenesis. During cellularisation (top), most pair-rule genes are regulated via zebra elements and become expressed in periodic patterns throughout the trunk of the embryo (green region). During gastrulation (middle), segmental patterns of pair-rule genes and segment-polarity genes emerge throughout the trunk (blue region) fairly simultaneously. Finally, during germ band extension (bottom), the segment-polarity network maintains the segment pattern via intercellular signalling throughout the trunk (red region). In short-germ embryos, for example, those of the red flour beetle *Tribolium castaneum*, segment patterning occurs continuously throughout germ band extension. Three germ bands of increasing age are depicted: the trunk elongates from the posterior as embryogenesis proceeds. Throughout the whole process, primary pair-rule genes exhibit oscillatory expression in the posterior segment addition zone (green regions). Pair-rule stripes then resolve (and may undergo frequency doubling) in the anterior segment addition zone (blue region). Finally, segment-polarity genes are expressed in segmental stripes starting just anterior to the segment addition zone (red regions). This phase of expression is thought to be regulated by a conserved signalling network [105,106]. **Abbreviations**: Cad, Caudal; Opa, Odd-paired.

To explore this question, I determined how many changes to the “wild-type” *Drosophila* simulation it would take in order to produce something resembling a sequential, “clock-and-wave-front” mode of segmentation. I managed to achieve this transition by way of a few plausible alterations, leaving the bulk of the network untouched (Fig 8C).

The only explicit regulatory changes that I made to the simulation were to the control logic of *eve*, *hairy*, and *opa*. First, I removed the regulation of *eve* by gap inputs (G2 in the model) and replaced it with early repression from Runt and Odd, both of which repress the *eve* late element in wild-type embryos (Fig 1A, right). Second, I removed the regulation of *hairy* by gap inputs (G1 in the model) and replaced it with autorepression, which is a common phenomenon for *her*/*hes* family genes [107,108]. Third, I tied the onset of *opa* expression to the decay of Cad by making Cad repress *opa*. These changes allowed the gap inputs G1 and G2 to be removed from the model and simplified the temporal control of the patterning process, putting everything downstream of Cad and Hairy.

I took this modified version of the *Drosophila* network and explored how it behaved at the tissue level, given various sets of initial conditions. I found that if I set up a repeating initial phase gradient of Hairy expression (i.e., for each cell along a double-segment repeat, Hairy expression begins at a slightly different point in its feedback cycle) and left initial Cad expression unchanged, the simulation output was essentially identical to before (Fig 8A and S12 Movie). This is because the waves of oscillating Hairy expression that sweep through the tissue while the early network is active are sufficient to correctly organise the pair-rule stripes of all the remaining primary pair-rule genes (including Eve), and therefore downstream patterning proceeds unperturbed.

However, I also discovered that if I removed all spatial patterning of Hairy from the initial conditions and instead established an anterior-to-posterior decay gradient of Cad (mimicking the retracting Cad domain seen in short-germ insects [87,109]), the simulation still produced the correct segmental output except that, now, this pattern emerged sequentially instead of simultaneously (Fig 8B and S13 Movie). As Cad decays and disappears from progressively more posterior cells over time, Opa turns on in an anterior-to-posterior wave, switching cells from the early network over to the late network. Cells in the posterior portion of the tissue synchronously passage through a repeating sequence of pair-rule gene expression (Hairy → Eve → Runt → Ftz/Odd → Hairy) until this point and subsequently differentiate into particular segment-polarity fates.

The modified network (Fig 8C, right) is thus compatible both with *Drosophila*-like pair-rule gene expression and with “clock-and-wave-front” pair-rule gene expression similar to that seen in, e.g., *Tribolium*. This finding suggests (1) that many of the regulatory interactions within the *Drosophila* pair-rule network might be conserved in short-germ arthropod species and (2) that long-germ and short-germ segmentation involve essentially similar patterning mechanisms and dynamics. Specifically, segment-polarity fate appears to be determined in both cases by the intersection of 2 sets of temporal signals, those carried by the pair-rule genes and those carried by broadly expressed extrinsic timing factors. In long-germ embryos, a periodic pattern of segment-polarity fates is achieved by directly imparting spatial information to the pair-rule genes using the gap system and stripe-specific elements. In short-germ embryos, however, retracting wave fronts of the timing factors provide this spatial information, and the gap genes play other roles.

## Discussion

### A revised view of the *Drosophila* pair-rule network reconciles long-germ and short-germ segmentation

In this manuscript, I have analysed the structure and dynamics of the *Drosophila* pair-rule network using a combination of simulated and experimental data to reveal how segment patterning is achieved. I have discovered a functional role for dynamic gap inputs in correctly phasing the pair-rule stripes and propose revised mechanisms for the patterning of the odd-numbered and even-numbered parasegment boundaries. In contrast to previous models based around the principle of static morphogen gradients, these mechanisms involve a coordinated interplay between intrinsic network dynamics and extrinsic spatiotemporal signals and do not necessarily require graded pair-rule activity.

These findings contribute to the evolving view of the role of Even-skipped, perhaps the best known of the pair-rule factors. Eve has long been known to be required for the expression of both sets of *en* stripes and hence both sets of parasegment boundaries [71,98]. Originally, Eve was thought to achieve this directly by activating *en*. Later, it was recognised that Eve does not regulate *en* directly but instead represses several other pair-rule factors that themselves repress *en* [75,76]; however, quantitative information inherent within the Eve stripes was still believed to establish the template for the *en* stripes [8,30]. This conclusion is challenged by the new model presented here, which suggests that static domains of Eve expression would cause a similar degree of pattern loss to that seen in *eve* mutant embryos. Instead, I propose that Eve conveys positional information largely via its expression dynamics, which are decoded downstream by the rest of the pair-rule gene network. (Interestingly, while “French flag” type morphogen gradient mechanisms have been proposed to underpin many developmental patterning systems, several modern studies have found that the reality often involves complex dynamics [58,110–113].)

The findings also clarify the relationship between long-germ and short-germ segmentation. I have shown that pair-rule patterning is fundamentally a temporal process and that it is therefore straightforward to convert the *Drosophila* pair-rule network into a clock-and-wave-front system. Under this scenario, the dynamic “early” network regulates oscillatory expression in the posterior of the tissue, while the switch-like “late” network stabilises these outputs into segmental patterns in the anterior. Given that long-germ insects such as *Drosophila* are derived from short-germ ancestors, it seems unlikely that this fluidity between simultaneous and sequential patterning modes is a coincidence. I therefore propose that output from the *Drosophila* early pair-rule network is developmentally homologous to the oscillatory pair-rule gene expression seen in the segment addition zone of short-germ insects such as *Tribolium*, while the output from the late pair-rule network is developmentally homologous to the stripe refinement and frequency doubling that occurs just anterior to the segment addition zone (Fig 8D). This hypothesis is strongly supported by a recent comparative study carried out by Andrew Peel and myself, which found that different phases of pair-rule gene expression in *Tribolium* exhibit the same correlations with *cad* and *opa* expression as seen in *Drosophila* [87]. Strikingly, the hypothesis implies that the “zebra” elements of *runt* and *odd* (which drive pair-rule patterns in the *Drosophila* blastoderm) represent the relictual components of some ancestral segmentation clock that drove oscillations of these genes with double-segment periodicity [50].

### How did long-germ segmentation evolve?

The evolutionary transition from short-germ segmentation to long-germ segmentation is thought to have been driven largely by selection for faster development. A segmentation clock can only tick so fast, and therefore represents a tight production bottleneck when generating segments: a longer body requires a longer time (S7A Fig). (Selection for quicker development may also underlie the evolutionary success of “pair-rule” patterning, seen in centipedes [114] as well as insects [115,116], which effectively halves the number of clock cycles required to produce a body of a given length.) In contrast, segmentation can be accomplished remarkably quickly when pair-rule gene expression is initialised with a periodic spatial pattern, as in *Drosophila*.

Mechanistically, the transition to long-germ segmentation seems to have involved 2 main changes: (1) a heterochronic shift in the deployment of the pair-rule network from posterior segment addition zone to blastoderm and (2) the elaboration of the posterior gap network and associated stripe-specific elements. These latter processes are likely to have occurred progressively along the AP axis, with intermediate forms exhibiting a composite mode of development: simultaneous for more anterior segments, sequential for more posterior segments [103]. The process also seems to have been facilitated by the re-use of anterior stripe-specific elements to also drive more posterior pair-rule stripes [23,117,118]. However, given the complexity of *Drosophila* pair-rule patterning, it has not been clear how this process could have evolved gradually and seamlessly, all the while maintaining a perfect segmental output pattern.

The findings in this paper significantly mitigate this problem. In my model of the *Drosophila* pair-rule network, 2 of the primary pair-rule genes are patterned by gap inputs while 3 are patterned by cross-regulation. However, the system works equally well if only 1 of these genes is explicitly patterned and the rest are cross-regulated (S12 Movie) or if all 5 are patterned by gap inputs and there is no cross-regulation (S8 Movie). Therefore, direct stripe patterning through stripe-specific elements and indirect stripe patterning through zebra elements can stand in for one another without affecting events downstream. (Similar conclusions, including a potential role for stripe shifts, were reached by a recent in silico evolution study focused on the *Drosophila* pair-rule genes [119].) Consequently, there is no need for multiple stripe-specific elements to have evolved simultaneously during the transition to long-germ segmentation: new stripe-specific elements could have evolved one by one, each time slotting into or replacing parts of the existing patterning machinery. Retention of an intact segmentation clock could also have buttressed gap-driven segment patterning during the transition, and such a scenario may apply today to insects such as *Nasonia* and *Bombyx*, which exhibit some evidence of both patterning modes [120–122]. The corollary of such flexibility between gap-driven and cross-regulated pair-rule gene expression is, however, that the dynamics of the gap network need to be highly constrained and should resemble those of the original segmentation clock. This appears to be the case [67].

### A trade-off between patterning speed and patterning robustness

Above, I stated that stripe-specific elements and cross-regulation are theoretically interchangeable for positioning pair-rule stripes. In the *Drosophila* blastoderm, the regulatory situation appears to be partially redundant: at least 3 of the primary pair-rule genes possess stripe-specific elements in any given pair-rule repeat, but only 2 of these genes lack zebra elements [16]. Why then have multiple pair-rule genes evolved stripe-specific elements, when patterning just a single gene in this way is theoretically sufficient for making segments? Additionally, why do genes such as *runt* retain a zebra element when they already have a full set of stripe-specific elements?

The first question has 2 likely answers. Using gap inputs to directly pattern multiple genes speeds up the first emergence of a correctly phased pair-rule repeat and hence reduces the total time required for segmentation as well as the minimum magnitude of the stripe shifts (S7B Fig; S6–S8 Movies). (The extreme scenario is that gap shifts are dispensed with entirely, as seems to have occurred in the anterior of the *Drosophila* trunk, based on comparisons with the scuttle fly *Megaselia abdita* [123,124].) The acquisition of extra stripe-specific elements could therefore have been driven by selection for marginal decreases to the length of development. Alternatively, these elements could have been selected for in order to mitigate irregularities in the patterns of upstream pair-rule genes. For example, *runt*, *ftz*, and *odd* all possess a stripe-specific element for stripe 3 [16], corresponding to a region of the blastoderm in which their repressor, Hairy, is inappropriately expressed early on [59].

While the stripe-specific elements of *runt*, *ftz*, and *odd* may therefore increase the speed of patterning, their output patterns are not as regular as those of *hairy* and *eve*, probably answering the second question. Irregular gap-driven pair-rule stripes, if not later refined by cross-regulation, would result in aberrant segment-polarity patterns. For example, cross-regulation of the *runt* pair-rule stripes appears to be absent in *Dichaete* mutant embryos, and this leads to severe and variable segmentation defects [87].

However, even if these *runt* stripes were as regular as those of *eve*, cross-regulation might still be required. The *eve* stripes are patterned with a precision of about 1% AP length, i.e., differing in position from embryo to embryo by roughly 1 nucleus [41]. This positional noise is equivalent to the spatial resolution of the final segment-polarity pattern, in which most stripes are only 1 cell wide (Fig 1B). Therefore, if the *eve* and *runt* stripes both showed positional variation of this magnitude, and this noise was uncorrelated, segmentation defects would likely occur. The benefit of the dynamic patterning carried out by the pair-rule network is that the influence of Eve activity on gene expression is not restricted to the cells currently within each Eve stripe but also extends to cells posterior to these stripes, like wakes stretching out behind moving ships. This means that entire pair-rule repeats become coordinated with the Eve stripes, regardless of where exactly these stripes are located in the blastoderm. Thus, spending extra time to cross-regulate is likely to minimise the consequences of noise in the original spatial signals, a trade-off that is characteristic of many developmental patterning systems [125].

### Assumptions, unexplained phenomena, and model limitations

I have presented theoretical evidence that posterior-to-anterior shifts of pair-rule stripes, driven by dynamic gap inputs, play a functional role in segment patterning. The plausibility of my model therefore rests on these shifts existing and being of an appropriate speed and magnitude. The basic model (only *hairy* and *eve* patterned by gap inputs) requires a minimum shift of 3 nuclei, while versions including greater control of pair-rule gene expression by gap inputs reduce this minimum to 1 or 2 nuclei (S6–S8 Movies). As yet, these shifts have only been measured in real embryos by comparing population estimates of absolute stripe position (in percent egg length) in fixed material of different ages [59,61]. Interpreting these measurements is not straightforward, but adjustments for nuclear migration suggest shifts of at least 2–3 nuclei for *eve* stripes 3–7, consistent with the requirements of the model. Recent developments in live imaging [126–128] mean that it should now be possible to test these estimates directly in individual embryos.

While the new model clarifies many aspects of segment patterning, it also highlights certain regulatory phenomena that are currently unexplained and require further study. For example, it is not clear how the timing of *slp* transcription is controlled, how exactly *prd* is spatially regulated, or which factors are responsible for activating the pair-rule genes.

Finally, the model is obviously simplistic and represents only the first step towards a modern, system-level understanding of the pair-rule network. For example, while the model demonstrates that morphogen-like effects are not necessary to account for pair-rule patterning, it does not rule them out. (Indeed, anterior-to-posterior expression shifts might well contribute to concentration-dependent spatial patterning of target genes, by helping shape the contours of the pair-rule stripes at the protein level.) Careful experimental characterisation of the quantitative relationship between the protein concentrations of input factors and the transcription rate of output factors could form the basis for future, more sophisticated models of *Drosophila* pair-rule patterning, which explore the roles of quantitative and stochastic effects. Future models of short-germ segmentation, on the other hand, could account for morphogenetic processes such as cell division and rearrangement [129–131] and/or incorporate known intercellular communication processes that occur both upstream (e.g., Notch-Delta signalling [132–134]) and downstream (e.g., Toll receptor codes [135,136]) of the pair-rule genes.

### Concluding remarks

In the introduction to this paper, I highlighted 3 recent findings related to pair-rule patterning: (1) that pair-rule orthologs exhibit oscillating expression in the segment addition zones of short-germ arthropods, (2) that pair-rule gene expression in long-germ insects is patterned by dynamic gap gene expression, and (3) that the pair-rule network in *Drosophila* undergoes an extensive topology change at gastrulation. I then presented evidence that dynamic Eve expression is integral to segment patterning and also that the *Drosophila* pair-rule network is broadly compatible with both simultaneous and sequential modes of segmentation.

I therefore propose the following evolutionary hypothesis, which ties together and potentially explains the 3 original findings. (1) Long-germ and short-germ segmentation are not dichotomous modes of patterning but rather alternative behaviours of a largely conserved pair-rule network. (2) In long-germ insects, ad hoc patterning of stripes by gap inputs stands in for the expression of particular pair-rule gene “clock enhancers”, meaning that gap gene networks are under strong selection to preserve dynamic behaviour. (3) In *Drosophila*, the “early” pair-rule network is derived from the “clock” part of an ancestral short-germ segmentation mechanism, while the “late” network is derived from the stabilising genetic interactions that would have originally established parasegment boundaries anterior to the segment addition zone.

These predictions can be tested in the future by comparative studies in emerging arthropod model species.

## Materials and methods

Wild-type in situ images are from a previously published data set deposited in the Dryad repository: http://dx.doi.org/10.5061/dryad.cg35k [137]. The *eve* mutation used was *eve*^*3*^ (gift of Bénédicte Sanson) and was balanced over *CyO hb*::*lacZ* (Boomington stock no. 6650) in order to easily distinguish homozygous mutant embryos. Whole mount double FISH, microscopy, and image analysis were carried out as described previously [32]. The *eve*/Eve doubles in Fig 4D were generated by incubating embryos with 1:1,000 rabbit anti-Eve [138] and 1:500 anti-rabbit 488 following in situ hybridisation to *eve*. Images were analysed using Fiji [139,140]: intensity profiles were produced using the “multi plot” function, while thresholded images were produced using the “make binary” tool. Simulations were coded in Python (www.python.org) using the libraries NumPy [141] and Matplotlib [142]. SBML-qual files were generated using the GINsim software (http://ginsim.org) [143]. All models and simulations are described in detail in S2 Text.

## Supporting information

S1 Fig Extended comparison between real and simulated pair-rule gene expression patterns. Expanded version of Fig 3 from the main text, showing all pairwise combinations between *hairy*, *eve*, *runt*, *ftz*, *odd*, and *slp*. (**A**) *hairy* and *eve* pair-rule stripes partially overlap during cellularisation. At gastrulation, *hairy* expression fades away, while the *eve* stripes narrow from the posterior and then also fade. (**B**) *eve* and *runt* pair-rule stripes partially overlap during cellularisation. The *eve* stripes become increasingly narrow at gastrulation, and the *runt* secondary stripes emerge anterior to the *eve* stripes. By early GBE, *eve* expression has faded away and *runt* expression has resolved into a regular, segmental pattern. The refinement of the *eve* stripes occurs more gradually in real embryos than in the simulation. Arrowheads in B”‘ indicate new *runt* expression related to the developing nervous system. (**C**) *eve* and *ftz* pair-rule stripes are at first expressed in complementary patterns. Starting from late cellularisation, they both narrow from the posterior (*eve* more than *ftz*). *eve* expression later fades away, while *ftz* persists. (**D**) *eve* and *odd* pair-rule stripes are at first expressed in complementary patterns, before both narrowing. *odd* secondary stripes emerge at the posterior of the narrowing *eve* domains, which then fade away, leaving segmental stripes of *odd*. (**E**) *hairy* and *runt* pair-rule stripes are expressed in complementary patterns during cellularisation. *hairy* expression then fades away, while *runt* transitions to segmental stripes. (**F**) *hairy* and *ftz* pair-rule stripes slightly overlap during cellularisation. *hairy* expression then fades away, while the *ftz* stripes narrow. (**G**) *hairy* and *odd* pair-rule stripes slightly overlap during cellularisation. *hairy* expression then fades away, while *odd* transitions to narrow segmental stripes. (**H**) *runt* and *ftz* pair-rule stripes partially overlap throughout cellularisation. At gastrulation, *runt* secondary stripes emerge to the posterior of the narrowing *ftz* stripes. Later, the *runt* primary stripes refine from the posterior, and the overlaps with *ftz* are lost. (**I**) *runt* and *odd* pair-rule stripes slightly overlap during cellularisation. These overlaps resolve at late cellularisation (slightly earlier than in the simulation). At gastrulation, new *odd* expression emerges just anterior to the *runt* primary stripes, while new *runt* expression emerges just posterior to the refining *odd* primary stripes. By early GBE there is a regular segmental pattern of abutting *odd* and *runt* stripes, separated by gaps. Arrowheads in I”‘ indicate new *runt* expression related to the developing nervous system. (**J**) The *ftz* and *odd* stripes are fairly congruent during cellularisation. At gastrulation, both narrow from the posterior, and the *odd* secondary stripes intercalate between them. Over the course of patterning, their anterior boundaries also become offset from one another. (**K**) The *slp* primary stripes emerge later than the *hairy* stripes, but share an anterior border with them. (The *slp* stripes in the simulation are too broad at this stage—they should be narrower than the *hairy* stripes.) *hairy* expression then fades away, while *slp* transitions to a segmental pattern. (**L**) The *slp* primary stripes emerge later than the *eve* stripes, and abut their anterior borders. (In the simulation, these *slp* stripes extend too far posteriorly, and overlap with *eve*. The reason is that the *eve* anterior borders would have stabilised by this point in real embryos, but they are still shifting in the simulation.) At gastrulation, the *eve* stripes narrow from the posterior and then fade, while slp transitions to segmental stripes. (**M**) The *slp* primary stripes emerge later than the *runt* primary stripes, and are offset slightly from their posterior boundaries. (The simulated *slp* domains are wider than the real *slp* domains.) At gastrulation, secondary stripes of both genes emerge between the primary stripes (the widths of the simulated *slp* stripes are now appropriate). The expression patterns become largely congruent, except at the posteriors of the *runt* primary stripes. These differences resolve later, when the *runt* primary stripes narrow. (**N**) The *slp* primary stripes emerge later than the *ftz* primary stripes, and partially overlap with them. At gastrulation, the secondary *slp* stripes emerge just anterior to the *ftz* domains, which narrow from the posterior, losing the overlaps with the *slp* primary stripes. (**O**) As for (N), the *slp* primary stripes partially overlap the *odd* primary stripes, and these overlaps are later lost by the *odd* stripes narrowing from the posterior. The secondary stripes of *odd* and *slp* intercalate between the primary stripes, and abut one other.
(DOCX)

S2 Fig Uncropped views of the embryos shown in S1 Fig. Each panel shows an uncropped version of the corresponding double fluorescent in situ image in S1 Fig. All panels show a whole embryo lateral view, anterior left, dorsal top. Embryos within each column are of approximately equal age. Scale = 100 μm.
(DOCX)

S3 Fig Some aspects of *prd* expression are not recovered by the simulation. This figure compares double FISH data of pair-rule gene expression with simulated transcript expression, as in Fig 3 and S2 Fig. (A) *prd* transcripts (magenta) are shown relative to *eve* (green), *odd* (blue) or *slp* (cyan) transcripts, at four different embryo ages (left) or simulation timepoints (right). Note that the figure presents a different set of developmental ages / simulated timepoints from those in Fig 3 and Supplementary Figure 3, in order to show cellularisation in greater temporal detail. At “early cellularisation”, the primary pair-rule genes are expressed but *prd* and *slp* are not; at “mid cellularisation”, *prd* is expressed but *slp* is not; at “late cellularisation”, *slp* is additionally expressed. While the simulated expression of *prd* is broadly appropriate early on (e.g. compare *prd* and *odd* expression at mid-cellularisation / T26), several aspects of *prd* expression are not recapitulated by the model. First, the changing phasing of *prd* posterior borders and *eve* anterior borders between mid-cellularisation and late cellularisation (see also Fig 5). Second, the splitting of the *prd* stripes at late cellularisation, slightly prior to the appearance of the secondary stripes of *odd* and *slp*. Third, the patterning of the *prd* “A” stripes (i.e. the narrow stripes formed from anterior portions of the early broad stripes, asterisks in the in situ images) and the consequent emergence of single-segment periodicity. (B) Uncropped views of the embryos shown in (A). Scale bars 100 μm.
(DOCX)

S4 Fig Gene expression at the odd-numbered parasegment boundaries. (A) Double FISH images of segmentation gene expression in gastrulation stage embryos (lateral view, anterior left, dorsal top). Each row shows a different pairwise comparison of the transcripts of the genes *eve* (magenta), *prd* (blue), *slp* (green), *en* (red), *odd* (yellow), and *wg* (cyan). From left to right, the panels in each row show: 1) a whole embryo view; 2) an enlarged view of pair-rule repeats 2–6; 3) the individual channel for the first gene listed in the row label; 4) the individual channel for the second gene listed in the row label. Arrowheads mark the locations of prospective odd-numbered parasegment boundaries. Scale bars 100 μm. (B) Summary schematic showing the relative expression of the Eve, Prd, and Slp protein domains, and the *en*, *wg*, and *odd* transcript domains, at the odd-numbered parasegment boundaries. (C) The same schematic as in B, with the addition of the regulatory interactions between Eve, Prd, and Slp and their target genes. Aside from the colours, which are changed to match the in situ images, this schematic is identical to Fig 5A.
(DOCX)

S5 Fig Expanded view of the *eve* mutant phenotype. This figure is an expansion of Fig 7 in the main text, showing additional whole embryo and single channel views. Enlarged views show stripes 2–6. Asterisks in (E) indicate the stripe 3 region. Scale = 50 μm. Note the quantitative traces below the images in (C). These plots show the AP intensity profiles of *runt* (green) and *slp* (blue) along a narrow ventral strip of the trunk of the two embryos pictured. In the wild-type embryo, the *runt* stripes are all (except stripe 7) roughly symmetrical and strongly expressed. In the *eve* mutant embryo, *runt* stripes 1–6 (which overlap with *odd* expression, see B), have much lower intensity than *runt* stripe 7 (which doesn’t overlap with *odd* expression, see B) and exhibit a sawtooth pattern, in which expression intensity decreases from anterior to posterior. The *slp* expression in the *eve* mutant embryo, while broad, does display a pair-rule modulation, which is in opposite phase to the downregulated *runt* stripes. Therefore, the same two regulatory interactions (repression of *runt* by Odd, and of *slp* by Runt) are evident in both the in situ data and the simulated data (Fig 7A’), but lead to slightly different expression patterns in each case, one quantitative and one qualitative.
(DOCX)

S6 Fig Stripe phasing depends on the speed of the gap shifts. Simulation output showing the expression patterns generated by the early pair-rule network, assuming various anterior-to-posterior shifts speeds of the gap inputs (shown in black). See supplementary movies for full simulation output (S1 Movie = 0x; S2 Movie = 1x; S3 Movie = 0.5x; S4 Movie = 2x; S5 Movie = 3x). Gap domain shift speeds are relative to the time delay for pair-rule protein synthesis / decay: a speed of 1x leads to a one nucleus offset between the anterior borders of transcript and protein domains, a speed of 2x leads to a two nucleus offset, and so on. See S2 Text for further details about the simulations.
(DOCX)

S7 Fig Selection for patterning speed could lead to increasingly elaborate regulation of pair-rule genes by the gap system. (A,B) Minimum duration required for generating a correct segment pattern (En, Odd, Slp repeats), under various simulation scenarios. Minimum simulation duration (Y-axis) is given as a multiple of the synthesis / decay delay parameter value of the pair-rule gene products (corresponding to 6 timesteps for all simulations shown in this manuscript). See S2 Text for further details on the various simulations. (A) Minimum time required to pattern a given number of segments by either simultaneous or sequential patterning, using the same network model as S12 and S13 Movies. Sequential patterning requires 12x the delay for the initial two segments, plus 8x the delay for every two additional segments. Simultaneous patterning requires a constant minimum time of 10x the delay, no matter the number of segments. (B) Minimum time (in multiples of the synthesis / decay delay parameter value) required for simultaneous patterning, for systems with increasingly elaborate gap patterning. If only *hairy* receives gap inputs (1), a minimum of time 10x the delay is required (network model as in simulation 12). If both *hairy* and *eve* receive gap inputs (2), the minimum is 7x the delay (network model as in simulation 10). If *runt* or *ftz*/*odd* additionally receive gap inputs (3), the minimum is 6x the delay (early network model as in simulations 6 or 7, respectively, plus late network model as for simulation 10). Finally, if all the primary pair-rule genes receive gap inputs (4), the minimum is 5x the delay (early network model as in simulation 8, plus late network model as for simulation 10). Note that the stripes of *ftz* and *odd* are considered a single pattern, because of their identical regulation in the simulations.
(DOCX)

S1 Text Regulatory interactions between the primary pair-rule genes during cellularisation. (PDF)

S2 Text Details of models and simulations. (PDF)

S1 File Functions needed for running network simulations. (PY)

S2 File Template script for network simulations. Corresponds to the network in Simulation 10 and Fig 2B.
(PY) Click here for additional data file.

S3 File Wild-type pair-rule network in SBML-qual format. Corresponds to the network in Simulation 10 and Fig 2B.
(SBML)

S4 File Modified pair-rule network in SBML-qual format. Corresponds to the network in Simulations 12 and 13, and Fig 8.
(SBML)

S1 Movie Simulation 1. Simulated expression of the primary pair-rule genes, assuming static gap inputs.
(MP4)

S2 Movie Simulation 2. Simulated expression of the primary pair-rule genes, assuming dynamic gap inputs.
(MP4)

S3 Movie Simulation 3. Simulated expression of the primary pair-rule genes, assuming slow gap shifts.
(MP4)

S4 Movie Simulation 4. Simulated expression of the primary pair-rule genes, assuming fast gap shifts.
(MP4)

S5 Movie Simulation 5. Simulated expression of the primary pair-rule genes, assuming very fast gap shifts.
(MP4)

S6 Movie Simulation 6. Pattern establishment when *hairy*, *eve*, and *runt* take gap inputs.
(MP4)

S7 Movie Simulation 7. Pattern establishment when *hairy*, *eve*, and *ftz*/*odd* take gap inputs.
(MP4)

S8 Movie Simulation 8. Pattern establishment when all primary pair-rule genes take gap inputs.
(MP4)

S9 Movie Simulation 9. Simulated behaviour of the whole pair-rule system, assuming static gap inputs.
(MP4)

S10 Movie Simulation 10. Simulated behaviour of the whole pair-rule system, assuming dynamic gap inputs.
(MP4)

S11 Movie Simulation 11. Pair-rule gene expression in a simulated *eve* mutant.
(MP4)

S12 Movie Simulation 12. Simultaneous patterning by the modified pair-rule network.
(MP4)

S13 Movie Simulation 13. Sequential patterning by the modified pair-rule network.
(MP4)

## Abbreviations

AP: anteroposterior
Cad: Caudal
*en*: *engrailed*
*eve*: *even-skipped*
FISH: fluorescent in situ hybridisation
*ftz*: *fushi tarazu*
*odd*: *odd-skipped*
Opa: Odd-paired
*prd*: *paired*
SBML: Systems Biology Markup Language
*slp*: *sloppy-paired*
*wg*: *wingless*
