# Supplementary material for: Dynamic patterning by the Drosophila pair-rule network reconciles long-germ and short-germ segmentation
Source: PLoS Biol. 2017 Sep 27;15(9):e2002439. doi: 10.1371/journal.pbio.2002439 (PMC5633203; doi:10.1371/journal.pbio.2002439)
Supplement: S1 Fig — Expanded version of Fig 3 from the main text, showing all pairwise combinations between hairy, eve, runt, ftz, odd, and slp. (A) hairy and eve pair-rule stripes partially overlap during cellularisation. At gastrulation, hairy expression fades away, while the eve stripes narrow from the posterior and then also fade. (B) eve and runt pair-rule stripes partially overlap during cellularisation. The eve stripes become increasingly narrow at gastrulation, and the runt secondary stripes emerge anterior to the eve stripes. By early GBE, eve expression has faded away and runt expression has resolved into a regular, segmental pattern. The refinement of the eve stripes occurs more gradually in real embryos than in the simulation. Arrowheads in B”‘ indicate new runt expression related to the developing nervous system. (C) eve and ftz pair-rule stripes are at first expressed in complementary patterns. Starting from late cellularisation, they both narrow from the posterior (eve more than ftz). eve expression later fades away, while ftz persists. (D) eve and odd pair-rule stripes are at first expressed in complementary patterns, before both narrowing. odd secondary stripes emerge at the posterior of the narrowing eve domains, which then fade away, leaving segmental stripes of odd. (E) hairy and runt pair-rule stripes are expressed in complementary patterns during cellularisation. hairy expression then fades away, while runt transitions to segmental stripes. (F) hairy and ftz pair-rule stripes slightly overlap during cellularisation. hairy expression then fades away, while the ftz stripes narrow. (G) hairy and odd pair-rule stripes slightly overlap during cellularisation. hairy expression then fades away, while odd transitions to narrow segmental stripes. (H) runt and ftz pair-rule stripes partially overlap throughout cellularisation. At gastrulation, runt secondary stripes emerge to the posterior of the narrowing ftz stripes. Later, the runt primary stripes refine from the posteri [file pbio.2002439.s001.docx]

**
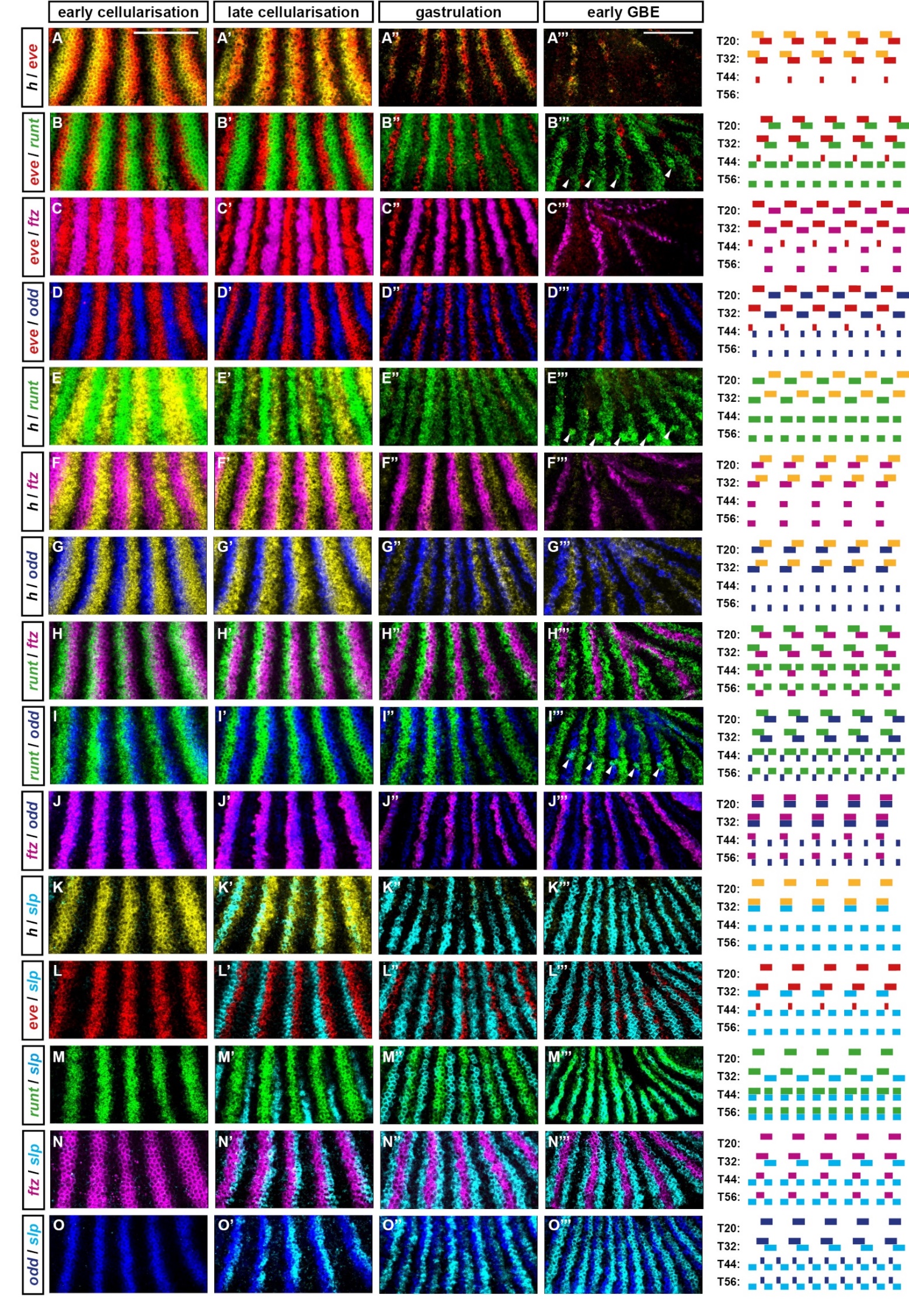
**

**Supplementary Figure 1: Extended comparison between real and simulated pair-rule gene expression patterns.**

Expanded version of Fig 3 from the main text, showing all pairwise combinations between *hairy*, *eve*, *runt*, *ftz*, *odd*, and *slp*.

(**A**) *hairy* and *eve* pair-rule stripes partially overlap during cellularisation. At gastrulation, *hairy* expression fades away, while the *eve* stripes narrow from the posterior and then also fade.

(**B**) *eve* and *runt* pair-rule stripes partially overlap during cellularisation. The *eve* stripes become increasingly narrow at gastrulation, and the *runt* secondary stripes emerge anterior to the *eve* stripes. By early GBE, *eve* expression has faded away and *runt* expression has resolved into a regular, segmental pattern. The refinement of the *eve* stripes occurs more gradually in real embryos than in the simulation. Arrowheads in B’’’ indicate new *runt* expression related to the developing nervous system.

(**C**) *eve* and *ftz* pair-rule stripes are at first expressed in complementary patterns. Starting from late cellularisation, they both narrow from the posterior (*eve* more than *ftz*). *eve* expression later fades away, while *ftz* persists.

(**D**) *eve* and *odd* pair-rule stripes are at first expressed in complementary patterns, before both narrowing. *odd* secondary stripes emerge at the posterior of the narrowing *eve* domains, which then fade away, leaving segmental stripes of *odd*.

(**E**) *hairy* and *runt* pair-rule stripes are expressed in complementary patterns during cellularisation. *hairy* expression then fades away, while *runt* transitions to segmental stripes.

(**F**) *hairy* and *ftz* pair-rule stripes slightly overlap during cellularisation. *hairy* expression then fades away, while the *ftz* stripes narrow.

(**G**) *hairy* and *odd* pair-rule stripes slightly overlap during cellularisation. *hairy* expression then fades away, while *odd* transitions to narrow segmental stripes.

(**H**) *runt* and *ftz* pair-rule stripes partially overlap throughout cellularisation. At gastrulation, *runt* secondary stripes emerge to the posterior of the narrowing *ftz* stripes. Later, the *runt* primary stripes refine from the posterior, and the overlaps with *ftz* are lost.

(**I**) *runt* and *odd* pair-rule stripes slightly overlap during cellularisation. These overlaps resolve at late cellularisation (slightly earlier than in the simulation). At gastrulation, new *odd* expression emerges just anterior to the *runt* primary stripes, while new *runt* expression emerges just posterior to the refining *odd* primary stripes. By early GBE there is a regular segmental pattern of abutting *odd* and *runt* stripes, separated by gaps. Arrowheads in I’’’ indicate new *runt* expression related to the developing nervous system.

(**J**) The *ftz* and *odd* stripes are fairly congruent during cellularisation. At gastrulation, both narrow from the posterior, and the *odd* secondary stripes intercalate between them. Over the course of patterning, their anterior boundaries also become offset from one another.

(**K**) The *slp* primary stripes emerge later than the *hairy* stripes, but share an anterior border with them. (The *slp* stripes in the simulation are too broad at this stage – they should be narrower than the *hairy* stripes.) *hairy* expression then fades away, while *slp* transitions to a segmental pattern.

(**L**) The *slp* primary stripes emerge later than the *eve* stripes, and abut their anterior borders. (In the simulation, these *slp* stripes extend too far posteriorly, and overlap with *eve*. The reason is that the *eve* anterior borders would have stabilised by this point in real embryos, but they are still shifting in the simulation.) At gastrulation, the *eve* stripes narrow from the posterior and then fade, while slp transitions to segmental stripes.

(**M**) The *slp* primary stripes emerge later than the *runt* primary stripes, and are offset slightly from their posterior boundaries. (The simulated *slp* domains are wider than the real *slp* domains.) At gastrulation, secondary stripes of both genes emerge between the primary stripes (the widths of the simulated *slp* stripes are now appropriate). The expression patterns become largely congruent, except at the posteriors of the *runt* primary stripes. These differences resolve later, when the *runt* primary stripes narrow.

(**N**) The *slp* primary stripes emerge later than the *ftz* primary stripes, and partially overlap with them. At gastrulation, the secondary *slp* stripes emerge just anterior to the *ftz* domains, which narrow from the posterior, losing the overlaps with the *slp* primary stripes.

(**O**) As for (N), the *slp* primary stripes partially overlap the *odd* primary stripes, and these overlaps are later lost by the *odd* stripes narrowing from the posterior. The secondary stripes of *odd* and *slp* intercalate between the primary stripes, and abut one other.
