# Supplementary material for: Dynamic patterning by the Drosophila pair-rule network reconciles long-germ and short-germ segmentation
Source: PLoS Biol. 2017 Sep 27;15(9):e2002439. doi: 10.1371/journal.pbio.2002439 (PMC5633203; doi:10.1371/journal.pbio.2002439)
Supplement: S2 Fig — Each panel shows an uncropped version of the corresponding double fluorescent in situ image in S1 Fig. All panels show a whole embryo lateral view, anterior left, dorsal top. Embryos within each column are of approximately equal age. Scale = 100 μm. (DOCX) [file pbio.2002439.s002.docx]

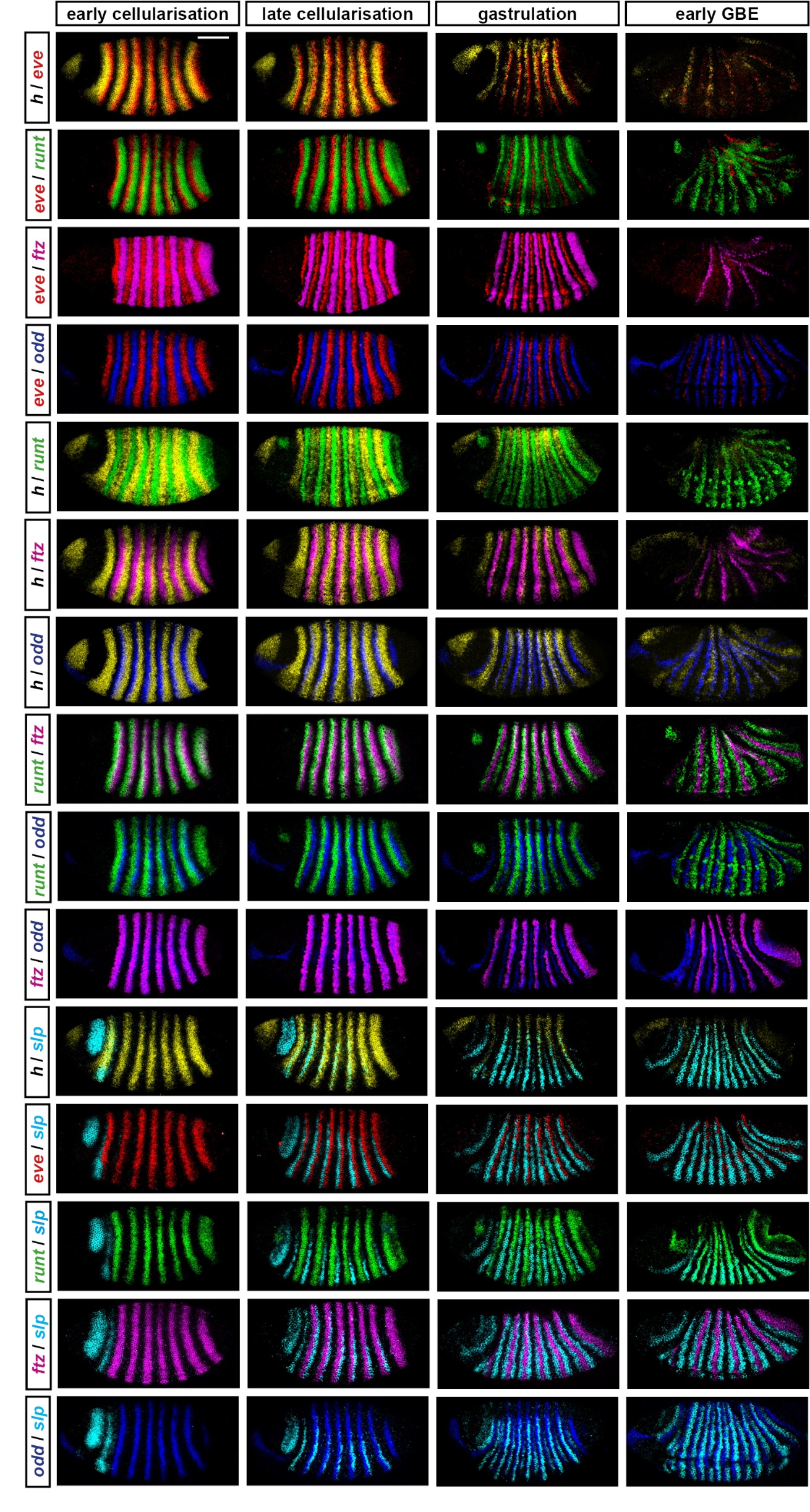


**Supplementary Figure 2: Uncropped views of the embryos shown in S1 Fig.**

Each panel shows an uncropped version of the corresponding double fluorescent in situ image in S1 Fig. All panels show a whole embryo lateral view, anterior left, dorsal top. Embryos within each column are of approximately equal age. Scale = 100 µm.
