# Supplementary material for: Dynamic patterning by the Drosophila pair-rule network reconciles long-germ and short-germ segmentation
Source: PLoS Biol. 2017 Sep 27;15(9):e2002439. doi: 10.1371/journal.pbio.2002439 (PMC5633203; doi:10.1371/journal.pbio.2002439)
Supplement: S3 Fig — This figure compares double FISH data of pair-rule gene expression with simulated transcript expression, as in Fig 3 and S2 Fig. (A) prd transcripts (magenta) are shown relative to eve (green), odd (blue) or slp (cyan) transcripts, at four different embryo ages (left) or simulation timepoints (right). Note that the figure presents a different set of developmental ages / simulated timepoints from those in Fig 3 and Supplementary Figure 3, in order to show cellularisation in greater temporal detail. At “early cellularisation”, the primary pair-rule genes are expressed but prd and slp are not; at “mid cellularisation”, prd is expressed but slp is not; at “late cellularisation”, slp is additionally expressed. While the simulated expression of prd is broadly appropriate early on (e.g. compare prd and odd expression at mid-cellularisation / T26), several aspects of prd expression are not recapitulated by the model. First, the changing phasing of prd posterior borders and eve anterior borders between mid-cellularisation and late cellularisation (see also Fig 5). Second, the splitting of the prd stripes at late cellularisation, slightly prior to the appearance of the secondary stripes of odd and slp. Third, the patterning of the prd “A” stripes (i.e. the narrow stripes formed from anterior portions of the early broad stripes, asterisks in the in situ images) and the consequent emergence of single-segment periodicity. (B) Uncropped views of the embryos shown in (A). Scale bars 100 μm. (DOCX) [file pbio.2002439.s003.docx]

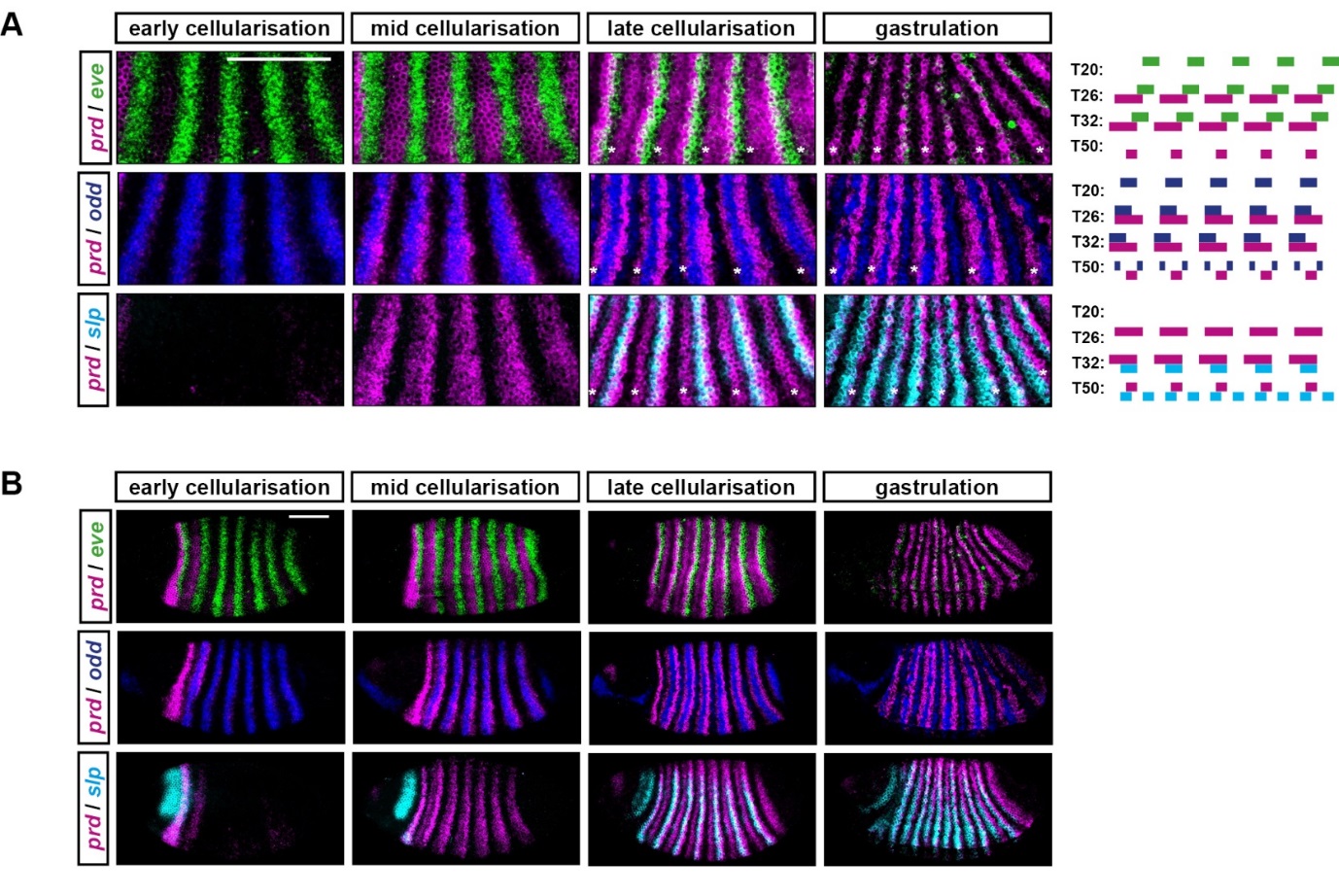


**Supplementary Figure 3: Some aspects of *prd* expression are not recovered by the simulation.**

This figure compares double FISH data of pair-rule gene expression with simulated transcript expression, as in Fig 3 and Supplementary Figure 2. (A) *prd* transcripts (magenta) are shown relative to *eve* (green), *odd* (blue) or *slp* (cyan) transcripts, at four different embryo ages (left) or simulation timepoints (right). Note that the figure presents a different set of developmental ages / simulated timepoints from those in Fig 3 and Supplementary Figure 3, in order to show cellularisation in greater temporal detail. At “early cellularisation”, the primary pair-rule genes are expressed but *prd* and *slp* are not; at “mid cellularisation”, *prd* is expressed but *slp* is not; at “late cellularisation”, *slp* is additionally expressed. While the simulated expression of *prd* is broadly appropriate early on (e.g. compare *prd* and *odd* expression at mid-cellularisation / T26), several aspects of *prd* expression are not recapitulated by the model. First, the changing phasing of *prd* posterior borders and *eve* anterior borders between mid-cellularisation and late cellularisation (see also Fig 5). Second, the splitting of the *prd* stripes at late cellularisation, slightly prior to the appearance of the secondary stripes of *odd* and *slp*. Third, the patterning of the *prd* “A” stripes (i.e. the narrow stripes formed from anterior portions of the early broad stripes, asterisks in the in situ images) and the consequent emergence of single-segment periodicity. (B) Uncropped views of the embryos shown in (A). Scale bars 100 µm.
