# Supplementary material for: Dynamic patterning by the Drosophila pair-rule network reconciles long-germ and short-germ segmentation
Source: PLoS Biol. 2017 Sep 27;15(9):e2002439. doi: 10.1371/journal.pbio.2002439 (PMC5633203; doi:10.1371/journal.pbio.2002439)
Supplement: S4 Fig — (A) Double FISH images of segmentation gene expression in gastrulation stage embryos (lateral view, anterior left, dorsal top). Each row shows a different pairwise comparison of the transcripts of the genes eve (magenta), prd (blue), slp (green), en (red), odd (yellow), and wg (cyan). From left to right, the panels in each row show: 1) a whole embryo view; 2) an enlarged view of pair-rule repeats 2–6; 3) the individual channel for the first gene listed in the row label; 4) the individual channel for the second gene listed in the row label. Arrowheads mark the locations of prospective odd-numbered parasegment boundaries. Scale bars 100 μm. (B) Summary schematic showing the relative expression of the Eve, Prd, and Slp protein domains, and the en, wg, and odd transcript domains, at the odd-numbered parasegment boundaries. (C) The same schematic as in B, with the addition of the regulatory interactions between Eve, Prd, and Slp and their target genes. Aside from the colours, which are changed to match the in situ images, this schematic is identical to Fig 5A. (DOCX) [file pbio.2002439.s004.docx]

**
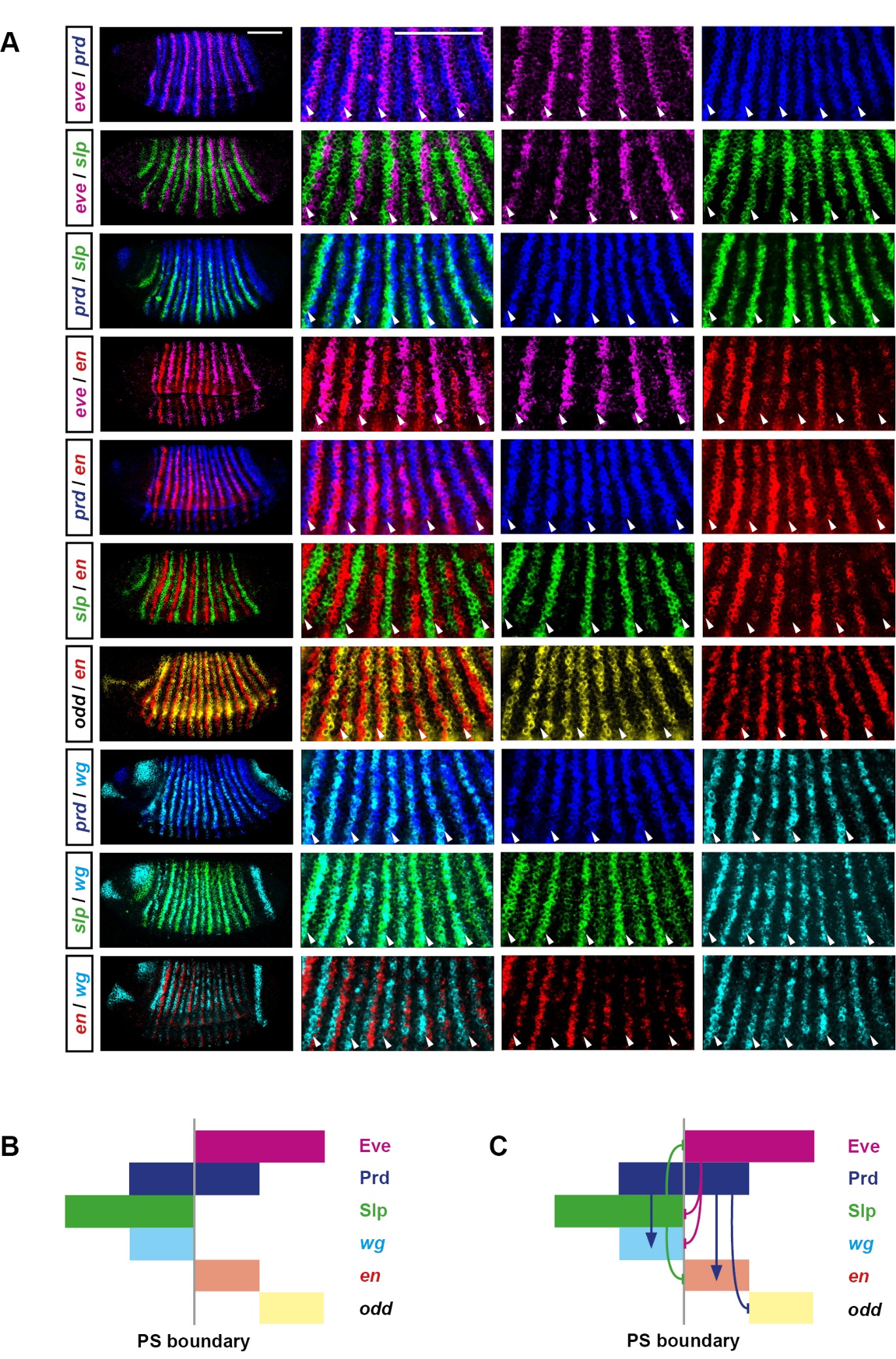
**

**Supplementary Figure 4: Gene expression at the odd-numbered parasegment boundaries.**

(A) Double FISH images of segmentation gene expression in gastrulation stage embryos (lateral view, anterior left, dorsal top). Each row shows a different pairwise comparison of the transcripts of the genes *eve* (magenta), *prd* (blue), *slp* (green), *en* (red), *odd* (yellow), and *wg* (cyan). From left to right, the panels in each row show: 1) a whole embryo view; 2) an enlarged view of pair-rule repeats 2-6; 3) the individual channel for the first gene listed in the row label; 4) the individual channel for the second gene listed in the row label. Arrowheads mark the locations of prospective odd-numbered parasegment boundaries. Scale bars 100 µm. (B) Summary schematic showing the relative expression of the Eve, Prd, and Slp protein domains, and the *en*, *wg*, and *odd* transcript domains, at the odd-numbered parasegment boundaries. (C) The same schematic as in B, with the addition of the regulatory interactions between Eve, Prd, and Slp and their target genes. Aside from the colours, which are changed to match the in situ images, this schematic is identical to Fig 5A.
