# Supplementary material for: Dynamic patterning by the Drosophila pair-rule network reconciles long-germ and short-germ segmentation
Source: PLoS Biol. 2017 Sep 27;15(9):e2002439. doi: 10.1371/journal.pbio.2002439 (PMC5633203; doi:10.1371/journal.pbio.2002439)
Supplement: S5 Fig — This figure is an expansion of Fig 7 in the main text, showing additional whole embryo and single channel views. Enlarged views show stripes 2–6. Asterisks in (E) indicate the stripe 3 region. Scale = 50 μm. Note the quantitative traces below the images in (C). These plots show the AP intensity profiles of runt (green) and slp (blue) along a narrow ventral strip of the trunk of the two embryos pictured. In the wild-type embryo, the runt stripes are all (except stripe 7) roughly symmetrical and strongly expressed. In the eve mutant embryo, runt stripes 1–6 (which overlap with odd expression, see B), have much lower intensity than runt stripe 7 (which doesn’t overlap with odd expression, see B) and exhibit a sawtooth pattern, in which expression intensity decreases from anterior to posterior. The slp expression in the eve mutant embryo, while broad, does display a pair-rule modulation, which is in opposite phase to the downregulated runt stripes. Therefore, the same two regulatory interactions (repression of runt by Odd, and of slp by Runt) are evident in both the in situ data and the simulated data (Fig 7A’), but lead to slightly different expression patterns in each case, one quantitative and one qualitative. (DOCX) [file pbio.2002439.s005.docx]

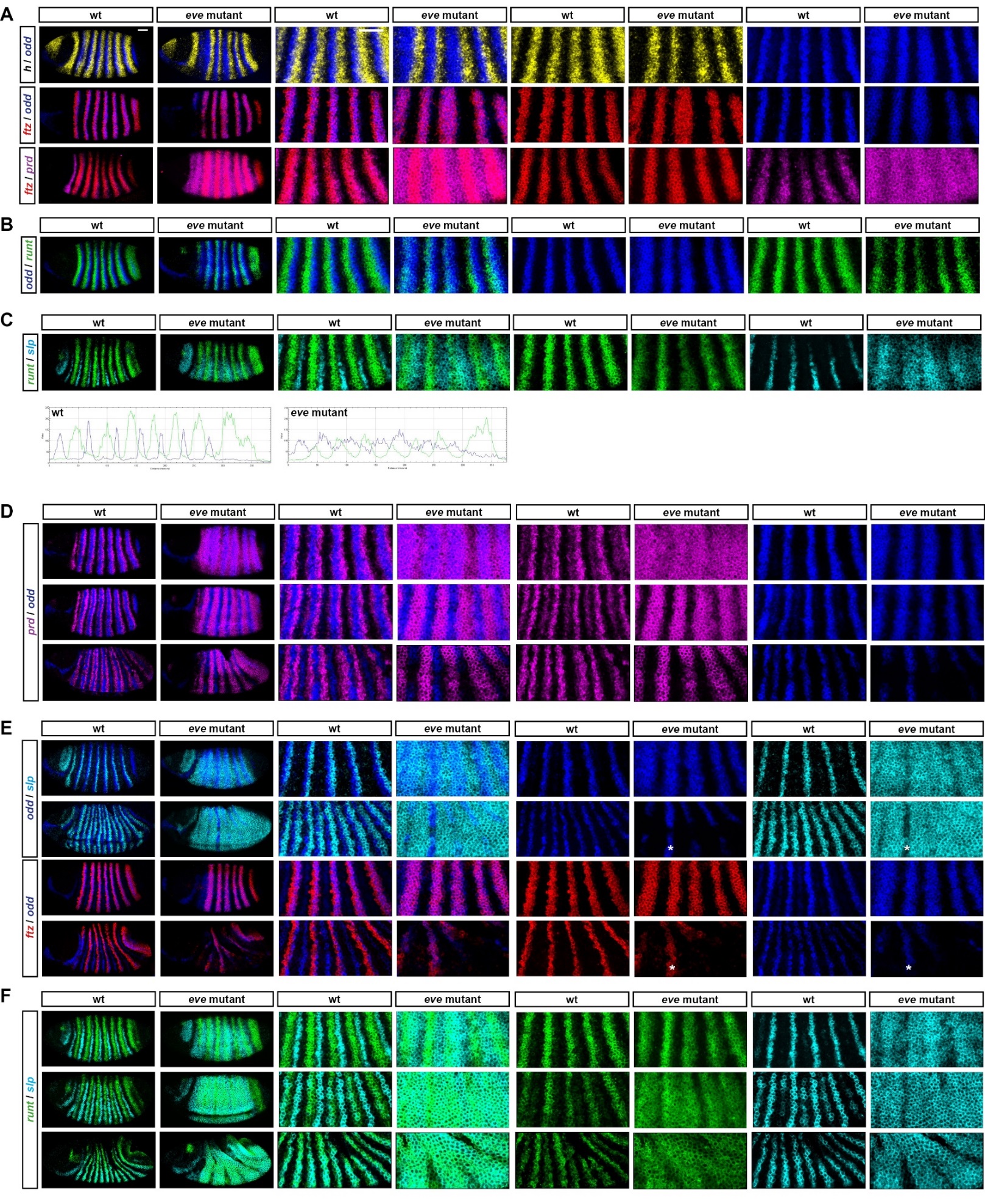


**Supplementary Figure 5: Expanded view of the *eve* mutant phenotype.**

This figure is an expansion of Fig 7 in the main text, showing additional whole embryo and single channel views. Enlarged views show stripes 2-6. Asterisks in (E) indicate the stripe 3 region. Scale = 50 µm.

Note the quantitative traces below the images in (C). These plots show the AP intensity profiles of *runt* (green) and *slp* (blue) along a narrow ventral strip of the trunk of the two embryos pictured. In the wild-type embryo, the *runt* stripes are all (except stripe 7) roughly symmetrical and strongly expressed. In the *eve* mutant embryo, *runt* stripes 1-6 (which overlap with *odd* expression, see B), have much lower intensity than *runt* stripe 7 (which doesn’t overlap with *odd* expression, see B) and exhibit a sawtooth pattern, in which expression intensity decreases from anterior to posterior. The *slp* expression in the *eve* mutant embryo, while broad, does display a pair-rule modulation, which is in opposite phase to the downregulated *runt* stripes. Therefore, the same two regulatory interactions (repression of *runt* by Odd, and of *slp* by Runt) are evident in both the in situ data and the simulated data (Fig 7A’), but lead to slightly different expression patterns in each case, one quantitative and one qualitative.
