# Supplementary material for: Dynamic patterning by the Drosophila pair-rule network reconciles long-germ and short-germ segmentation
Source: PLoS Biol. 2017 Sep 27;15(9):e2002439. doi: 10.1371/journal.pbio.2002439 (PMC5633203; doi:10.1371/journal.pbio.2002439)
Supplement: S6 Fig — Simulation output showing the expression patterns generated by the early pair-rule network, assuming various anterior-to-posterior shifts speeds of the gap inputs (shown in black). See supplementary movies for full simulation output (S1 Movie = 0x; S2 Movie = 1x; S3 Movie = 0.5x; S4 Movie = 2x; S5 Movie = 3x). Gap domain shift speeds are relative to the time delay for pair-rule protein synthesis / decay: a speed of 1x leads to a one nucleus offset between the anterior borders of transcript and protein domains, a speed of 2x leads to a two nucleus offset, and so on. See S2 Text for further details about the simulations. (DOCX) [file pbio.2002439.s006.docx]

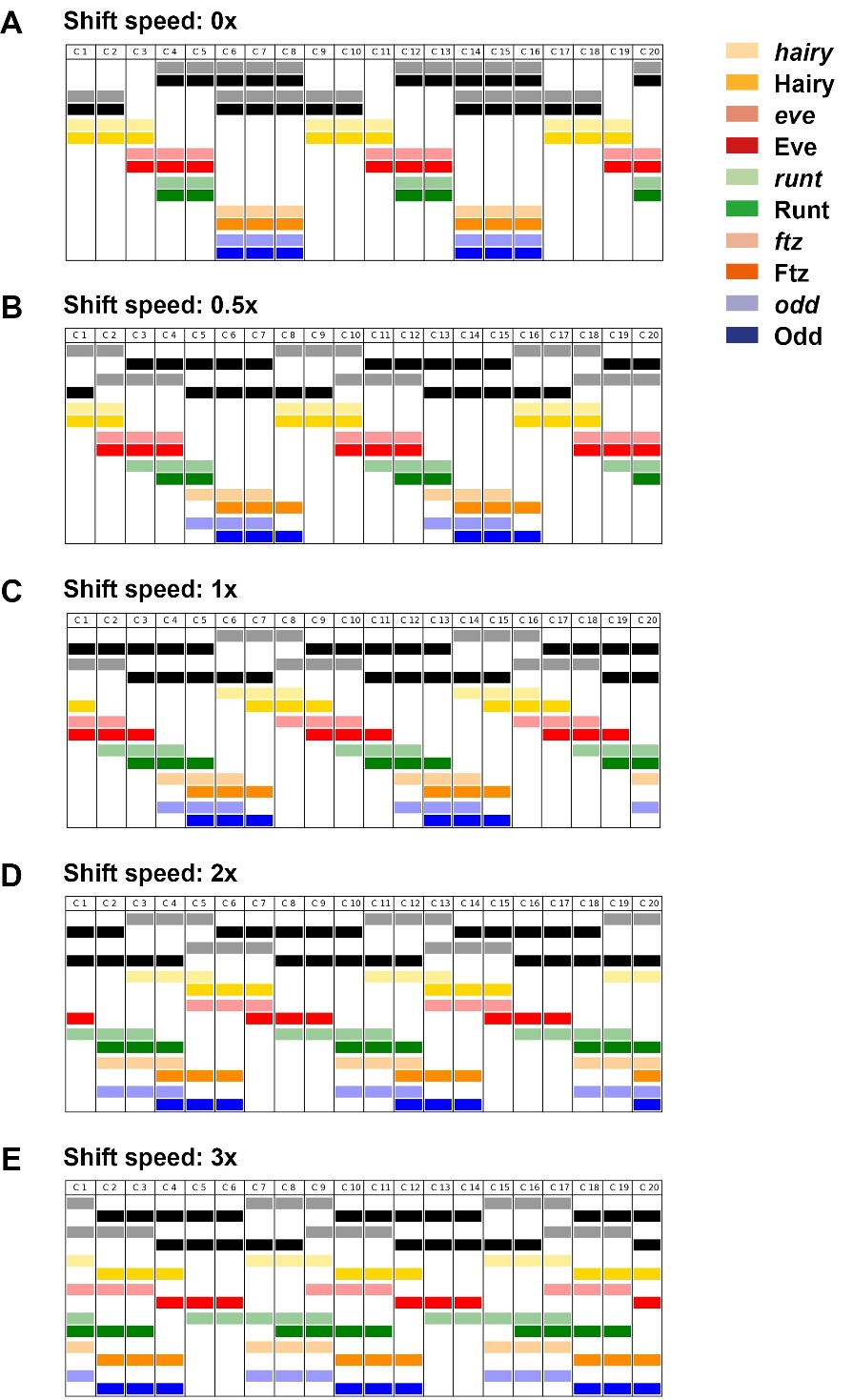


**Supplementary Figure 6: Stripe phasing depends on the speed of the gap shifts.**

Simulation output showing the expression patterns generated by the early pair-rule network, assuming various anterior-to-posterior shifts speeds of the gap inputs (shown in black). See supplementary movies for full simulation output (S1 Movie = 0x; S2 Movie = 1x; S3 Movie = 0.5x; S4 Movie = 2x; S5 Movie = 3x). Gap domain shift speeds are relative to the time delay for pair-rule protein synthesis / decay: a speed of 1x leads to a one nucleus offset between the anterior borders of transcript and protein domains, a speed of 2x leads to a two nucleus offset, and so on. See S2 Text for further details about the simulations.
