# Supplementary material for: Dynamic patterning by the Drosophila pair-rule network reconciles long-germ and short-germ segmentation
Source: PLoS Biol. 2017 Sep 27;15(9):e2002439. doi: 10.1371/journal.pbio.2002439 (PMC5633203; doi:10.1371/journal.pbio.2002439)
Supplement: S7 Fig — (A,B) Minimum duration required for generating a correct segment pattern (En, Odd, Slp repeats), under various simulation scenarios. Minimum simulation duration (Y-axis) is given as a multiple of the synthesis / decay delay parameter value of the pair-rule gene products (corresponding to 6 timesteps for all simulations shown in this manuscript). See S2 Text for further details on the various simulations. (A) Minimum time required to pattern a given number of segments by either simultaneous or sequential patterning, using the same network model as S12 and S13 Movies. Sequential patterning requires 12x the delay for the initial two segments, plus 8x the delay for every two additional segments. Simultaneous patterning requires a constant minimum time of 10x the delay, no matter the number of segments. (B) Minimum time (in multiples of the synthesis / decay delay parameter value) required for simultaneous patterning, for systems with increasingly elaborate gap patterning. If only hairy receives gap inputs (1), a minimum of time 10x the delay is required (network model as in simulation 12). If both hairy and eve receive gap inputs (2), the minimum is 7x the delay (network model as in simulation 10). If runt or ftz/odd additionally receive gap inputs (3), the minimum is 6x the delay (early network model as in simulations 6 or 7, respectively, plus late network model as for simulation 10). Finally, if all the primary pair-rule genes receive gap inputs (4), the minimum is 5x the delay (early network model as in simulation 8, plus late network model as for simulation 10). Note that the stripes of ftz and odd are considered a single pattern, because of their identical regulation in the simulations. (DOCX) [file pbio.2002439.s007.docx]

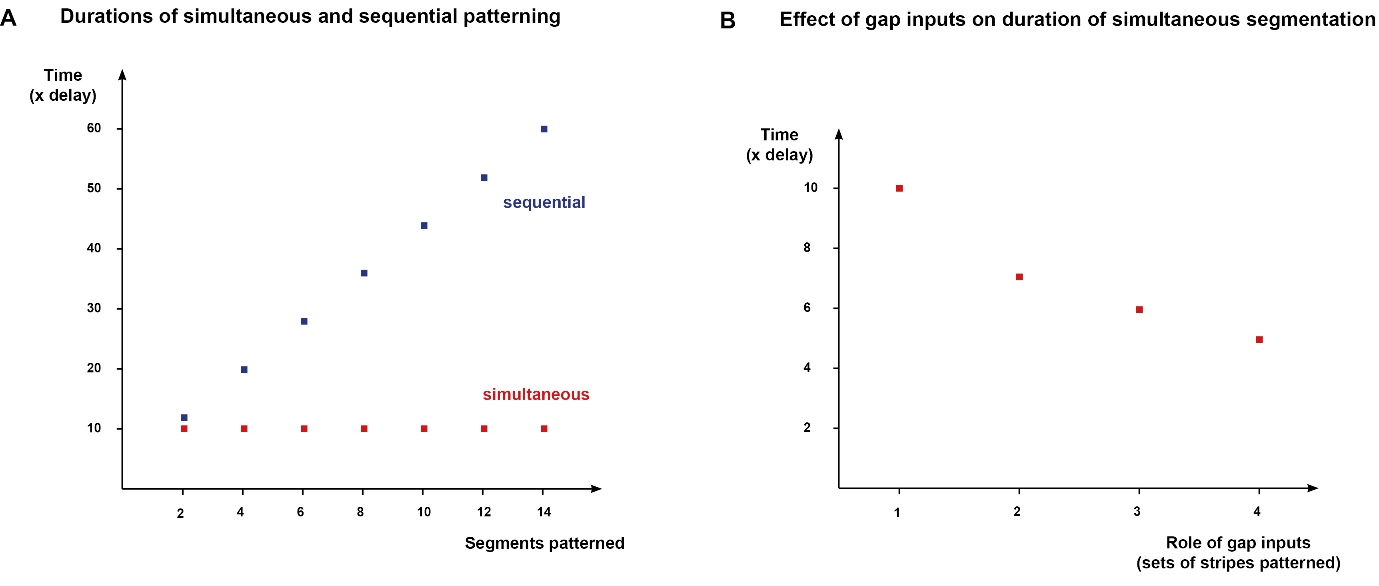


**Supplementary Figure 7: Selection for patterning speed could lead to increasingly elaborate regulation of pair-rule genes by the gap system.**

(A,B) Minimum duration required for generating a correct segment pattern (En, Odd, Slp repeats), under various simulation scenarios. Minimum simulation duration (Y-axis) is given as a multiple of the synthesis / decay delay parameter value of the pair-rule gene products (corresponding to 6 timesteps for all simulations shown in this manuscript). See Text S2 for further details on the various simulations.

(A) Minimum time required to pattern a given number of segments by either simultaneous or sequential patterning, using the same network model as S12 Movie and S13 Movie. Sequential patterning requires 12x the delay for the initial two segments, plus 8x the delay for every two additional segments. Simultaneous patterning requires a constant minimum time of 10x the delay, no matter the number of segments.

(B) Minimum time (in multiples of the synthesis / decay delay parameter value) required for simultaneous patterning, for systems with increasingly elaborate gap patterning. If only *hairy* receives gap inputs (1), a minimum of time 10x the delay is required (network model as in simulation 12). If both *hairy* and *eve* receive gap inputs (2), the minimum is 7x the delay (network model as in simulation 10). If *runt* or *ftz*/*odd* additionally receive gap inputs (3), the minimum is 6x the delay (early network model as in simulations 6 or 7, respectively, plus late network model as for simulation 10). Finally, if all the primary pair-rule genes receive gap inputs (4), the minimum is 5x the delay (early network model as in simulation 8, plus late network model as for simulation 10). Note that the stripes of *ftz* and *odd* are considered a single pattern, because of their identical regulation in the simulations.
