# Supplementary material for: Dynamic patterning by the Drosophila pair-rule network reconciles long-germ and short-germ segmentation
Source: PLoS Biol. 2017 Sep 27;15(9):e2002439. doi: 10.1371/journal.pbio.2002439 (PMC5633203; doi:10.1371/journal.pbio.2002439)
Supplement: S1 Text — (PDF) [file pbio.2002439.s008.pdf]

Supplementary Text 1:

Regulatory interactions between the primary pair-rule genes during cellularisation

This supplementary document concerns the cross-regulatory interactions between the five primary pair-rule genes (*hairy*, *eve*, *runt*, *ftz*, and *odd*) during cellularisation. Looking at each gene in turn, I examine the evidence for its expression being directly regulated by the other primary pair-rule factors. The conclusions form the basis for the topology of the "early" pair-rule network presented in Fig 1A of the main text.

Gene regulatory network models have been characterised as "intellectual syntheses" of the combined evidence (typically expression data) from a large number of diverse experiments [1]. In order to analyse the control logic of the *Drosophila* primary pair-rule genes, the main sources of evidence I consider are wild-type stripe phasings, expression patterns in mutant and transgenic embryos, and regulatory element reporter studies. I have collated relevant observations from the literature, and complement these with new double fluorescent in situ hybridisation (FISH) data from *hairy*, *eve* and *runt* mutant embryos.

An important aspect of this analysis is determining the timing of particular expression changes, in order to disentangle regulatory interactions that form part of the early network from those that only relate to the late network. Based on this analysis, I conclude that Hairy and Eve act largely as "input-only" factors in the early pair-rule network, and organise the expression of the remaining pair-rule genes around themselves.

Note that figures in this Supplement may refer to embryos as being at "phase 1", "phase 2", or "phase 3". As defined in Clark and Akam (2016) [2], "phase 1" refers to early cellularisation, when most pair-rule gene expression is controlled in an ad hoc manner by stripe-specific elements, "phase 2" refers to mid-cellularisation, characterised by regular periodic patterns usually driven by zebra elements, and "phase 3" refers to late cellularisation and gastrulation, when the transition to single segment periodicity occurs. The early network operates during phase 2, while the late network operates during phase 3.

Regulation of *hairy*

Regulatory elements

hairy possesses a full set of stripe-specific elements (1+5, 2+6, 3+4, 7, reviewed in [3]). However, *hairy* is not known to possess any kind of periodically expressed element. This suggests that the majority of its regulation comes directly from the gap system.

No evidence for regulation by Eve, Ftz, or Odd

In wild-type embryos, expression of *hairy* overlaps with Eve, Ftz and Odd (inferred from [4] and [5]), indicating that it is not repressed by any of them. In agreement with this, the *hairy*

expression pattern is not significantly altered by ectopic expression of Eve, Ftz or Odd, aside from repression of *hairy* stripe 1 in HS-Odd embryos [6–8]. *hairy* expression is also largely normal in *ftz* and *odd* mutant embryos [3,8,9].

In contrast, *hairy* expression is rather abnormal in *eve* mutant embryos: *hairy* stripe 2 becomes repressed, while the remaining stripes exhibit abnormal widths and spacings [10–12] (S1 Text-Fig 1). However, these changes are unlikely to reflect direct regulation of *hairy* by Eve. *hairy* stripe 2, which is sensitive to Slp, is presumably repressed by the ectopic Slp expression that occurs in *eve* mutant embryos [3,13]. The subtler effects on the remaining stripes are as yet unexplained, but it has been suggested that they reflect a patterning role of the early broad expression of Eve during cycles 12 and 13 [12,14]. If so, the effects on the *hairy* stripes are likely indirect, and mediated via subtle changes to gap gene expression.

Little evidence for regulation by Runt

It has been proposed previously that *hairy* is directly repressed by Runt. In wild-type embryos the *hairy* stripes are out of phase with the *runt* stripes (Fig 4C in main text), and *runt* mutant embryos exhibit ectopic expression of *hairy* [10–12,15]. Direct repression by Runt is thought to cause the splitting of the *hairy* stripe 3+4 element into distinct stripes [16,17], and is also thought to be involved in proper separation of *hairy* stripes 6 and 7.

However, this evidence is not clear cut. Runt cannot be absolutely required for the splitting of *hairy* 3+4, as this splitting still occurs, at least temporarily, in *runt* mutant embryos [12] (S1 Text-Fig 2). In addition, *hairy* expression is not significantly affected by heatshock-mediated misexpression of Runt in blastoderm stage embryos [18,19]. At 30 minutes after heatshock (when direct effects would be expected to be evident), only *hairy* stripe 1 is repressed by ectopic Runt. Later weakening of stripes 2, 5, and 6 in these experiments could be indirect effects, perhaps via documented effects of Runt on gap gene expression [19,20]. Notably, *hairy* stripes 3 and 4 do not appear at all repressed in the HS-Runt embryos, indicating either that the splitting of stripes 3 and 4 in wild-type embryos is not mediated via direct repression of the 3+4 element by Runt, or that this element is sensitive to Runt only temporarily.

The *hairy* stripes seem to establish fairly normally in *runt* mutant embryos, with the fusions of stripes 3/4 and stripes 6/7 not occurring until late cellularisation (S1 Text–fig 2). This casts further doubt on a direct role for Runt in specifying the *hairy* pair-rule pattern. First, spatial inputs from Runt are not required for the initial emergence of seven *hairy* stripes (cf. [16]). Second, the late appearance of ectopic *hairy* expression indicates that any direct regulation of *hairy* by Runt may be specific to the late network. Suggestively, similar fusions of *hairy* stripes 3/4 and 6/7 occur at gastrulation in *opa* mutant embryos [2], indicating that Runt and Opa might cooperate to repress Hairy, in the same way that they cooperate to repress Odd.

Conclusion

In summary, there is little convincing evidence for direct regulation of *hairy* by other primary pair-rule factors during cellularisation. Eve and Runt activity certainly influence *hairy*

expression, but their effects seem to be either indirect, or restricted to later phases of patterning. Clear evidence of direct repression during cellularisation is limited to specific anterior stripes (e.g. stripe 1 is sensitive to Runt and Odd, and stripe 2 is sensitive to Slp). It therefore appears that *hairy* stripes 3-7 are a direct output of the gap system.

Regulation of *even-skipped*

Regulatory elements

Like *hairy*, *eve* also possesses a full set of stripe-specific elements (1, 2, 3+7, 4+6, 5; reviewed in [3]). It also possesses a "late" element generating strong expression in seven narrow stripes [21,22]. However the expression of the late element does not kick in until the end of cellularisation, after the primary stripes of the secondary pair-rule genes *prd* and *slp* have already emerged [3]. The switchover from the stripe-specific elements to the late element appears to be regulated by Opa [2]. The *eve* pattern is therefore likely to be specified by gap inputs during cellularisation, with pair-rule inputs taking control at gastrulation.

No evidence for regulation by Hairy or Ftz

In wild-type embryos, *eve* and *hairy* expression overlaps throughout segmentation [11,23] (Fig 3A in main text), so *eve* is evidently not repressed by Hairy. Consistent with this interpretation, *eve* expression is not directly affected by expression of Hairy fused to an activator domain [17], and *eve* expression is normal until late cellularisation in *hairy* mutant embryos (S1 Text–fig 3).

eve is also not repressed by Ftz: *eve* expression is not repressed by ectopic Ftz at any stage of segmentation [7], nor activated by ectopic Ftz fused to an activation domain [24], and *eve* expression does not change in *ftz* mutants [10]. Therefore, there is clear cut evidence that neither Hairy nor Ftz directly regulate *eve*.

Little evidence in favour of regulation by Runt or Odd

In contrast, mutant and misexpression studies indicate that both Runt and Odd can repress *eve* expression [8,12,18]. However, in order to determine whether these regulatory interactions are relevant to the early pair-rule network, it is important to analyse the timing of any changes to *eve* expression.

All *eve* stripes are effectively repressed by ectopic Odd or Runt in late cellularisation stage embryos [8,18]. However, during mid-cellularisation only *eve* stripe 1 is repressed by HS-Odd [8,25], and only *eve* stripe 2 is significantly repressed by HS-Runt [19]. Minor changes to some of the other *eve* stripes also occur in cellularisation stage HS-Runt embryos [19], but the time at which these changes were observed (30-40 minutes after the end of a 20 minute heatshock) is consistent with them being indirect responses to Runt. Runt activity is known to affect the gap system [19,20], therefore it is possible that the observed changes to *eve* expression are mediated by misexpressed gap factors.

The evidence from misexpression experiments therefore suggests that *eve* expression is not directly regulated by Runt or Odd until late cellularisation, aside from stripe-specific effects on stripes 1 and 2. This conclusion is also supported by analysis of the evidence from mutant embryos. *eve* expression is normal in *odd* mutant embryos [14] and also in embryos deficient for the entire *odd*, *sob*, *drm* cluster of *odd-skipped* paralogs (my data, not shown). In *runt* mutant embryos, the *eve* stripes show abnormal spacing [10,12,14,26,27], but this is likely to be an indirect effect, resulting from regulatory effects of the early broad Runt domain on the gap system (see above). Fairly regular *eve* stripes are maintained until late cellularisation, when *eve* expression expands markedly (S1 Text–fig 4). This delay is further evidence that Runt is not important for patterning *eve* until late cellularisation.

This conclusion is also supported by observations from *hairy* mutant embryos, which exhibit significant coexpression of *eve* and *runt* (S1 Text–fig 5). Anterior expansion of the *runt* stripes in these embryos means that *runt* is expressed throughout the *eve* stripes for most of cellularisation, however, aside from in stripe 2, *eve* expression is not significantly repressed until late cellularisation. *eve* transcript expression is also likely to overlap with Runt protein expression during cellularisation in wild-type embryos, although not so extensively. (Note that while overlaps are obvious between Eve protein and Runt protein [28] and between *eve* transcript and *runt* transcript (S1 Text–fig 5), an *eve* RNA/Runt protein double would be required for explicit confirmation that *eve* is expressed in Runt-positive cells in wild-type embryos.)

Conclusion

In summary, perturbing the expression of other pair-rule genes does not cause widespread gain or loss of *eve* expression until late cellularisation, suggesting that they do not directly regulate *eve* prior to this. Absence of Runt activity perturbs the spacing of the *eve* stripes, but, as discussed above, this effect is likely to be indirect (although I would not rule out a subtle role for Runt in quantitatively regulating/refining the *eve* stripes in wild-type). The precise, regularly spaced *eve* stripes in cellularising embryos therefore appear to be largely a direct output of the gap system. Note however that, as seen for *hairy* stripes 1 and 2, pair-rule cross-regulation does seem to be important for *eve* stripes 1 and 2 (which are sensitive to Odd and Runt, respectively). These effects might be mediated via the *eve* stripe 1 and *eve* stripe 2 elements, which would therefore take both gap and pair-rule inputs. Obvious pair-rule control of *eve* expression in stripes 3-7 does not become evident until late cellularisation, and is presumably mediated by the *eve* late element.

Regulation of *runt*

Regulatory elements

runt has both a full set of stripe-specific elements and a zebra element [3]. There are individual elements for all seven stripes, although the elements for stripes 1 and 2 also drive some expression in stripe 7 [3]. The zebra element is expressed during both cellularisation and gastrulation [29]. The boundaries of the wild-type *runt* stripes could therefore plausibly come

from either the gap system or the pair-rule system, depending on how these various elements interact.

No evidence for regulation by Ftz

In cellularising wild-type embryos, *runt* expression overlaps both *eve* and *ftz* expression (Fig 4C in main text), suggesting it is not repressed by either Eve or Ftz. This conclusion is largely supported by the evidence from mutant and misexpression studies. Consistent with Ftz not regulating *runt*, HS-Ftz has no direct effect on *runt* expression at any stage of segmentation [7], and *runt* expression is normal during cellularisation in *ftz* mutant embryos [30].

No clear-cut evidence for regulation by Eve

The evidence relating to Eve is more complicated. In *eve* mutant embryos a fairly normal pair-rule pattern of *runt* forms initially, although several of the stripes are subsequently downregulated (S1 Text–fig 6; Fig 7 in main text). As argued below and in the main text, this repression is likely indirect, mediated by ectopic Odd. *runt* expression is also affected by ectopic Eve, although the effect is variable depending on the stage at which Eve is misexpressed [6]. HS-Eve represses *runt* stripes 1-6 in gastrulating embryos, as expected from the sharp boundaries between *eve* and *runt* expression that form at late cellularisation in wild-type (see S1 Text–fig 5). However, heatshocks in younger embryos can cause a dramatic broadening of the *runt* stripes, implicating Eve as an activator of *runt*. It is not clear whether this latter effect is direct or indirect, nor at which point exactly the switch from activation to repression occurs. Note though that the Eve protein is not known to act as a transcriptional activator [6,31–34].

Good evidence for regulation by Hairy and Odd

In cellularising wild-type embryos, the anterior and posterior borders of the *runt* stripes correspond closely to borders of *hairy* and *odd* expression, respectively (Fig 4C in main text). Consistent with this stripe phasing, I find good evidence that both Hairy and Odd pattern *runt* expression during cellularisation, although the conclusions I draw are somewhat different than previous analyses.

In *hairy* mutant embryos, *runt* is expressed in a fairly normal seven stripe pattern, with weak expression in between the stripes [10,30] (S1 Text–fig 5). Because this pattern still contains seven well-defined stripes, it has been previously interpreted as representing the normal expression from the stripe-specific elements overlain on a background of low-level ectopic expression from a derepressed zebra element [3,30]. Under this view, the spatial pattern of *runt* expression in wild-type embryos would be determined mainly by the gap system, while the zebra element would play only a minor, redundant role.

However, I interpret this pattern of *runt* expression in *hairy* mutant embryos differently. Direct comparison with the *eve* stripes indicates that the strong stripes of *runt* shift anteriorly

relative to their normal positions by around 1-2 nuclei, and are therefore not equivalent to the stripes observed in wild-type (S1 Text–fig 5). This indicates that repression from Hairy normally specifies the anterior boundaries of the *runt* stripes in wild-type embryos, presumably through the *runt* zebra element. Protein fusion misexpression experiments indicate that this regulatory interaction is direct [17].

The evidence in favour of repression by Odd is fairly straightforward. *runt* expression is partially repressed by HS-Odd during cellularisation, while in *odd* mutant embryos the *runt* stripes broaden slightly [8]. This broadening presumably occurs at the posteriors of the *runt* stripes and reflects activation of *runt* expression in nuclei which are Odd positive but Hairy negative in wild-type, and therefore free of both Odd and Hairy in the mutant embryos. As discussed in the main text, derepression of *odd* expression in *eve* mutant embryos leads to a subsequent downregulation of the *runt* stripes, although this repression of *runt* is not total (S1 Text–fig 6; Fig 7 in main text). Repression by Odd is also likely to be responsible for much of the residual periodicity of *runt* expression seen in *hairy* mutant embryos (S1 Text–fig 5).

Conclusion

In summary, although *runt* possesses a full set of stripe-specific elements, the precise spatial regulation of its expression at mid cellularisation seems to be determined mainly by pair-rule inputs, specifically repression from Hairy and Odd. Positional information from these pair-rule factors seems to largely override spatial cues from the gap system in determining stripe boundaries, as demonstrated by expanded *runt* expression in *hairy* and *odd* mutants. The *runt* zebra element is therefore probably more important for patterning than are the stripe-specific elements, and indeed it is sufficient for fairly normal segmentation in their absence [35]. However, it is clear that the stripe-specific elements do exert some influence on *runt* expression throughout cellularisation, as the control of Hairy and Odd over the *runt* expression pattern is not total. For example, the *runt* stripes are only downregulated in HS-Odd and *eve* mutant embryos rather than completely lost, while in wild-type embryos *runt* stripe 3 emerges from within a domain of Hairy expression. Therefore, while accurate to a first approximation, the model of early *runt* regulation depicted in Fig 1A is clearly an oversimplification of the more elaborate control logic seen in the embryo.

<u>Regulation of *ftz* and *odd*</u>

I analyse the regulation of *ftz* and *odd* simultaneously, because they exhibit very similar expression during cellularisation in a variety of genetic and experimental backgrounds. Any patterning differences between the two genes are noted and discussed.

Regulatory elements

In contrast to *hairy*, *eve* and *runt*, the genes *ftz* and *odd* have traditionally been considered secondary pair-rule genes, regulated by other pair-rule factors rather than by the gap system [9,36,37]. However, more recent analyses have revealed that their early expression is regulated

by stripe-specific elements, and they are now classified as primary pair-rule genes [3,38]. Despite this status upgrade, they are evidently not as extensively regulated by the gap system as are the other three primary pair-rule genes. Neither *ftz* nor *odd* possesses a full set of stripe-specific regulatory elements: *ftz* has 1+5, 2+7 and 3+6, and so lacks an element for stripe 4, while *odd* has 1+5 and 3+6, and so lacks elements for stripes 2, 4 and 7 [3]. Both genes also possess a zebra element expressed throughout cellularisation [3,39]. The zebra elements are solely responsible for patterning the stripes that do not have their own stripe-specific elements. However, the boundaries of the remaining stripes could be plausibly specified by either gap factors or pair-rule factors, depending on how the elements interact.

Strong evidence for regulation by Eve and Hairy

Cross-regulatory interactions with other pair-rule genes have long been recognised to play an important role in determining the expression of *ftz* and *odd* during cellularisation, as their expression tends to be strongly perturbed in pair-rule mutant embryos (for example, for *ftz*, see [9,40]).

In wild-type embryos, the anterior borders of the *ftz* and *odd* stripes are closely associated with the posterior borders of the *eve* stripes, while the posterior borders of the *ftz* and *odd* stripes are closely associated with the anterior borders of the *hairy* stripes (Fig 4C in main text). These patterns suggest that *ftz* and *odd* are repressed by both Eve and Hairy. This interpretation is supported by the expression of *ftz* and *odd* in mutant embryos.

In *eve* mutant embryos, *ftz* and *odd* are expressed in periodic patterns that are fairly complementary with the *hairy* stripes (S1 Text–fig 1). Notably, fusions of *odd* stripes 1+2 correlate with the loss of *hairy* stripe 2 discussed above, indicating that the periodicity of *odd* expression in these embryos relies on repression by Hairy. In addition, the clear gaps between the posteriors of the *hairy* stripes and the anteriors of the *odd* stripes that are seen in wild-type embryos (asterisks in S1 Text–fig 1) are lost, indicating that these are usually established in response to repression by Eve.

Expression changes between wild-type and *eve* mutant embryos are not so obvious for *ftz*, consisting of slight broadening of certain stripes (particularly 2 and 4), plus almost complete loss of stripe 1 (*odd* stripe 1 is also lost ventrally). However, the fact that stripes 2-6 of *ftz* and *odd* are expressed in extremely similar patterns to each other in *eve* mutant embryos (S1 Text–fig 7) indicates that both genes are subject to the same patterning by Hairy. (The stripes of *odd* are consistently slightly broader than those of *ftz*, suggesting that *odd* is repressed slightly less effectively by Hairy.) I have not investigated the differential expression of *ftz* stripe 1 and *odd* stripe 1 in *eve* mutant embryos, but this phenomenon indicates that their stripe 1 elements are each subject to their own bespoke regulation.

The broadening of *ftz* stripe 4 (which lacks a stripe-specific element) in *eve* mutants is consistent with the anterior boundary of this stripe normally being patterned by repression from Eve. However, it is likely that the anterior boundaries of the remaining *ftz* stripes are initially positioned by gap factors so as to slightly overlap with Eve expression in wild-type embryos (see discussion of this topic in [2]). This would explain why they are located slightly

anterior to the *odd* anterior boundaries in wild-type embryos, and why they do not significantly expand in *eve* mutant embryos

The evidence from *hairy* mutants is more dramatic. In these embryos, *ftz* and *odd* expression initially expands throughout almost the entire trunk (S1 Text–fig 8), indicating that general repression by Hairy is crucial for their patterning during early cellularisation. As previously noted [23], this derepression is more extensive than would be predicted based on the spatial pattern of Hairy expression in fixed embryos, and therefore likely contributes to the severe and variable cuticle phenotypes of *hairy* null mutants [41,42].

Direct repression of *ftz* and *odd* by Hairy and Eve is also supported by evidence from heatshock-mediated misexpression: both genes are repressed by HS-Eve [6], and by HS-Hairy [43,44]. In addition, both genes are ectopically expressed in response to expression of Hairy fused to an activator domain [17]. Notably, *odd* is more effectively repressed by HS-Eve than is *ftz*, a difference that has been suggested to stem from different inherent sensitivities of *ftz* and *odd* to Eve activity [6]. However, this phenomenon could equally stem from Ftz autoactivation [45,46], and a resulting difficulty in turning *ftz* expression off once it has already been established.

No evidence for regulation by Runt

In wild-type embryos, the stripes of *ftz* and *odd* overlap the posteriors of the *runt* stripes during cellularisation, suggesting that *ftz* and *odd* are not repressed by Runt. Consistent with this conclusion, *ftz* is not repressed after Runt misexpression, nor activated by Runt fused to an activator domain [17,18] (effects on *odd* were not reported).

However, *ftz* and *odd* do exhibit altered expression in *runt* mutants during cellularisation, notably a weakening of stripes 3 and 6 [10,12,27,40,47] (S1 Text–fig 4). Again, as discussed for the other pair-rule genes, this effect of Runt on stripe width and spacing appears to be indirect. In the mutant embryos, the patterns of *ftz* and *odd* still correspond negatively with those of *eve* and *hairy* (S1 Text–fig 2; S1 Text–fig 4), with the effects on stripes 3 and 6 apparently reflecting the partial fusion of *hairy* stripes 3-4 and 6-7, as well as more subtle changes to the relative phasings of the Hairy and Eve stripes (see [26]). It thus seems clear that *ftz* and *odd* are directly repressed by Hairy and Eve, but not by Runt.

Regulation by each other

Interestingly, Ftz and Odd appear to directly activate each other during early cellularisation: stripes of *ftz* broaden shortly after Odd misexpression, and vice versa [7,8]. However, all seven stripes of *ftz* or *odd* still appear (albeit weakened slightly) in embryos mutant for the other gene, indicating that this activation is not necessary for their expression [3,7,8,48].

Conclusion

In conclusion, the stripes of *ftz* and *odd* are largely defined by pair-rule cross-repression, presumably via their zebra elements. The posterior boundaries of the stripes of both genes are defined by repression from Hairy, while the anterior boundaries of the *odd* stripes are defined by repression by Eve. The anterior boundaries of the *ftz* stripes seem to be defined by Eve in certain cases, but by gap inputs in others.

The significant role of the zebra elements explains why *ftz* and *odd* need not possess a full set of stripe-specific elements: the necessary spatial information for patterning their stripes can be provided instead via Eve and Hairy. However, it is clear that certain stripe-specific elements do play non-redundant roles in patterning: for example, establishing *ftz* and *odd* stripe 3 expression despite the late-resolving Hairy pattern in this region, or helping to differentially position the anterior boundaries of the *ftz* and *odd* stripes.

It is also clear that there are still questions to be answered about the regulation of *ftz* and *odd* (particularly of *ftz*) during cellularisation. How do the stripe-specific and zebra elements interact, what is the role of Ftz autoactivation in patterning the *ftz* stripes, and what is the explanation for the surprisingly crucial role for Hairy in generating a periodic output pattern?

REFERENCES

1. Peter IS, Faure E, Davidson EH. Predictive computation of genomic logic processing functions in embryonic development. Proc Natl Acad Sci U S A. 2012;109:16434–42.
2. Clark E, Akam M. Odd-paired controls frequency doubling in Drosophila segmentation by altering the pair-rule gene regulatory network. Elife. 2016;5:e18215.
3. Schroeder MD, Greer C, Gaul U. How to make stripes: deciphering the transition from non-periodic to periodic patterns in Drosophila segmentation. Development. 2011;138:3067–78.
4. Pisarev A, Poustelnikova E, Samsonova M, Reinitz J. FlyEx, the quantitative atlas on segmentation gene expression at cellular resolution. Nucleic Acids Res. 2009;37:560–6.
5. Clark E, Akam M. Data from: Odd-paired controls frequency doubling in Drosophila segmentation by altering the pair-rule gene regulatory network. Dryad Digit Repos. 2016;doi: 10.5061/dryad.cg35k.
6. Manoukian AS, Krause HM. Concentration-dependent activities of the even-skipped protein in Drosophila embryos. Genes Dev. 1992;6:1740–51.
7. Nasiadka A, Krause HM. Kinetic analysis of segmentation gene interactions in Drosophila embryos. Development. 1999;126:1515–26.
8. Saulier-Le Dréan B, Nasiadka A, Dong J, Krause HM. Dynamic changes in the functions of Odd-skipped during early Drosophila embryogenesis. Development. 1998;125:4851–61.
9. Howard K, Ingham P. Regulatory interactions between the segmentation genes fushi tarazu, hairy, and engrailed in the Drosophila blastoderm. Cell. 1986;44:949–57.

10. Ingham P, Gergen P. Interactions between the pair-rule genes runt, hairy, even-skipped and fushi tarazu and the establishment of periodic pattern in the Drosophila embryo. Development. 1988;104:51–60.

11. Hooper KL, Parkhurst SM, Ish-Horowicz D. Spatial control of hairy protein expression during embryogenesis. Development. 1989;107:489–504.

12. Vavra SH, Carroll SB. The zygotic control of Drosophila pair-rule gene expression: II. Spatial repression by gap and pair-rule gene products. Development. 1989;107:663–72.

13. Andrioli LP, Oberstein AL, Corado MSG, Yu D, Small S. Groucho-dependent repression by Sloppy-paired 1 differentially positions anterior pair-rule stripes in the Drosophila embryo. Dev Biol. 2004;276:541–51.

14. Frasch M, Levine M. Complementary patterns of even-skipped and fushi tarazu expression involve their differential regulation by a common set of segmentation genes in Drosophila. 1987;1:981–95.

15. Riddihough G, Ish-Horowicz D. Individual stripe regulatory elements in the Drosophila hairy promoter respond to maternal, gap, and pair-rule genes. Genes Dev. 1991;5:840–54.

16. Hartmann C, Taubert H, Jäckle H. A two-step mode of stripe formation in the Drosophila blastoderm requires interactions among primary pair rule genes. Mech 0. 1994;45:3–13.

17. Jiménez G, Pinchin SM, Ish-Horowicz D. In vivo interactions of the Drosophila Hairy and Runt transcriptional repressors with target promoters. EMBO J. 1996;15:7088–98.

18. Manoukian AS, Krause HM. Control of segmental asymetry in Drosophila embryos. Development. 1993;118:785–96.

19. Tsai C, Gergen JP. Gap gene properties of the pair-rule gene runt during Drosophila segmentation. Development. 1994;120:1671–83.

20. Chen H, Xu Z, Mei C, Yu D, Small S. A System of Repressor Gradients Spatially Organizes the Boundaries of Bicoid-Dependent Target Genes. Cell. 2012;149:618–29.

21. Goto T, Macdonald P, Maniatis T. Early and late periodic patterns of even skipped expression are controlled by distinct regulatory elements that respond to different spatial cues. Cell. 1989;57:413–22.

22. Harding K, Hoey T, Warrior R, Levine M. Autoregulatory and gap gene response elements of the even-skipped promoter of Drosophila. EMBO J. 1989;8:1205–12.

23. Carroll SB, Laughon A, Thalley BS. Expression, function, and regulation of the hairy segmentation protein in the Drosophila embryo. Genes Dev. 1988;2:883–90.

24. Nasiadka A, Grill A, Krause HM. Mechanisms regulating target gene selection by the homeodomain-containing protein Fushi tarazu. Development. 2000;127:2965–76.

25. Goldstein RE, Cook O, Dinur T, Pisante A, Karandikar UC, Virginia W. An eh1-Like Motif in Odd-skipped Mediates Recruitment of Groucho and Repression In Vivo. Mol Cell Biol. 2005;25:10711–20.

26. Warrior R, Levine M. Dose-dependent regulation of pair-rule stripes by gap proteins and the initiation of segment polarity. Development. 1990;110:759–67.

27. Jaynes JB, Fujioka M. Drawing lines in the sand: even skipped et al. and parasegment boundaries. Dev Biol. 2004;269:609–22.

28. Kania MA, Bonner AS, Duffy JB, Kania MA, Bonner AS, Duffy JB, et al. The Drosophila segmentation gene runt encodes a novel nuclear regulatory protein that is also expressed in the developing nervous system. Genes Dev. 1990;1701–13.

29. Klingler M, Soong J, Butler B, Gergen JP. Disperse versus compact elements for the regulation of runt stripes in Drosophila. Dev Biol. 1996;177:73–84.

30. Klingler M, Gergen JP. Regulation of runt transcription by Drosophila segmentation genes. Mech Dev. 1993;43:3–19.

31. Han K, Manley JL. Transcriptional repression by the Drosophila even-skipped protein: Definition of a minimal repression domain. Genes Dev. 1993;7:491–503.

32. Austin RJ, Biggin MD. A domain of the even-skipped protein represses transcription by preventing TFIID binding to a promoter: repression by cooperative blocking. Mol Cell Biol. 1995;15:4683–93.

33. Um M, Li C, Manley JL. The transcriptional repressor even-skipped interacts directly with TATA- binding protein. Mol Cell Biol. 1995;15:5007–16.

34. Fujioka M, Yusibova GL, Patel NH, Brown SJ, Jaynes JB. The repressor activity of Even-skipped is highly conserved, and is sufficient to activate engrailed and to regulate both the spacing and stability of parasegment boundaries. Development. 2002;129:4411–21.

35. Butler BA, Soong J, Gergen JP. The Drosophila segmentation gene runt has an extended cis-regulatory region that is required for vital expression at other stages of development. Mech Dev. 1992;39:17–28.

36. Gergen JP, Butler BA. Isolation of the Drosophila segmentation gene runt and analysis of its expression during embryogenesis. Genes Dev. 1988;2:1179–93.

37. Baumgartner S, Noll M. Network of interactions among pair-rule genes regulating paired expression during primordial segmentation of Drosophila. Mech Dev. 1990;33:1–18.

38. Yu Y, Pick L. Non-periodic cues generate seven ftz stripes in the Drosophila embryo. Mech Dev. 1995;50:163–75.

39. Hiromi Y, Kuroiwa A, Gehring WJ. Control elements of the Drosophila segmentation gene fushi tarazu. Cell. 1985;43:603–13.

40. Carroll SB, Scott MP. Zygotically active genes that affect the spatial expression of the fushi tarazu segmentation gene during early Drosophila embryogenesis. Cell. 1986;45:113–26.

41. Ingham P, Pinchin S. Genetic analysis of the hairy locus in Drosophila melanogaster. Genetics. 1985;463–86.

42. Lardelli M, Ish-Horowicz D. Drosophila hairy pair-rule gene regulates embryonic patterning outside its apparent stripe domains. Development. 1993;118:255–66.

43. Ish-Horowicz D, Pinchin SM. Pattern abnormalities induced by ectopic expression of the Drosophila gene hairy are associated with repression of ftz transcription. Cell. 1987;51:405–15.

44. Kok K, Ay A, Li LM, Arnosti DN. Genome-wide errant targeting by Hairy. Elife. 2015;4:1–21.

45. Hiromi Y, Gehring WJ. Regulation and Function of the Drosophila Segmentation Gene fushi tarazu. Cell. 1987;50:963–74.

46. Schier A, Gehring W. Direct homeodomain–DNA interaction in the autoregulation of the fushi tarazu gene. Nature. 1992;356:804–7.

47. Lawrence PA, Johnston P. Pattern formation in the Drosophila embryo: allocation of cells to parasegments by even-skipped and fushi tarazu. Development. 1989;105:761–7.

48. Mullen JR, DiNardo S. Establishing parasegments in Drosophila embryos: roles of the odd-skipped and naked genes. Dev Biol. 1995;169:295–308.

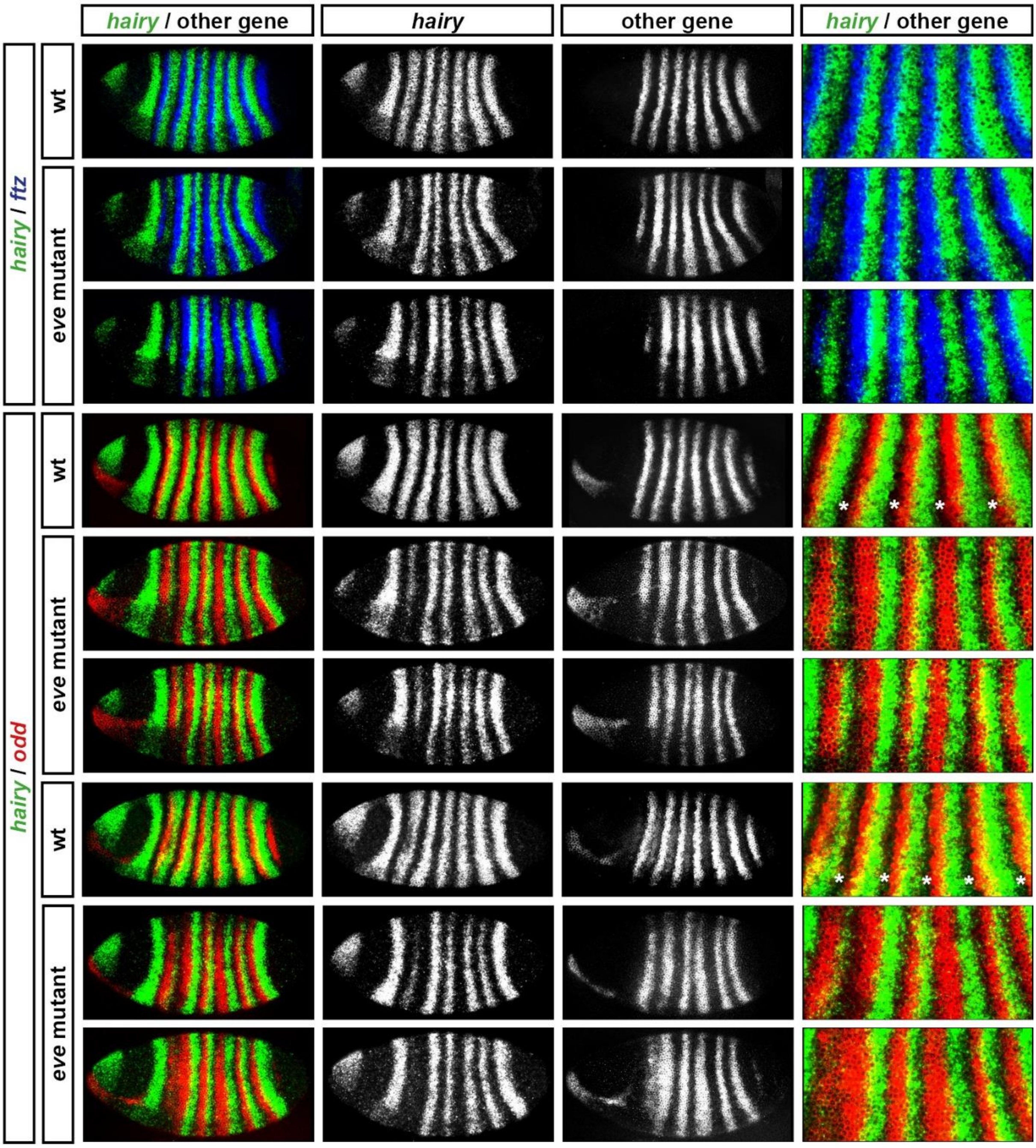

S1 Text–fig 1: *ftz* and *odd* are patterned by Hairy in *eve* mutant embryos

Expression of *ftz* and *odd* relative to *hairy* in wild-type and *eve* mutant embryos. For the *hairy*/*odd in situs*, the upper three panels show embryos at mid cellularisation while the lower three panels show embryos at late cellularisation. Two different mutant embryos are shown for each time point. Note loss of *hairy* stripe 2 in *eve* mutant embryos, and corresponding anterior expansion of *odd* stripe 2. Note also the broadened stripes 2 and 4 of both *ftz* and *odd*, and the reduction of the clear gaps between the posteriors of the *hairy* stripes and the anteriors of the *odd* stripes (asterisks in wild-type embryos). In addition to the repression of *hairy* stripe 2, *hairy* stripes 3-6 exhibit abnormal widths and spacing.

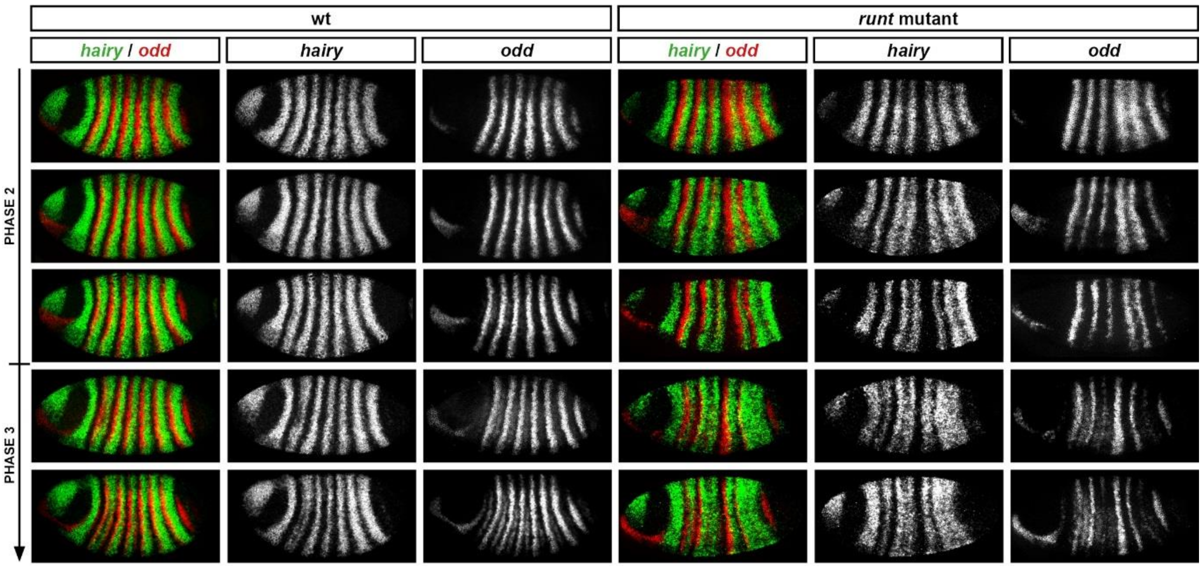

S1 Text–fig 2: *hairy* stripes do not fuse until late cellularisation in *runt* mutant embryos

Relative expression of *hairy* and *odd* in wild-type and *runt* mutant embryos. The *hairy* stripes establish fairly normally (row 1), but the gap between stripes 4 and 5 widens during mid-cellularisation (rows 2-3), then fusions between stripes 3-4 and 6-7 occur at late cellularisation (rows 4 and 5). *odd* expression correlates negatively with *hairy* expression at all stages. Arrow indicates increasing developmental age. Single channel images are shown in greyscale to the right of the double channel images.

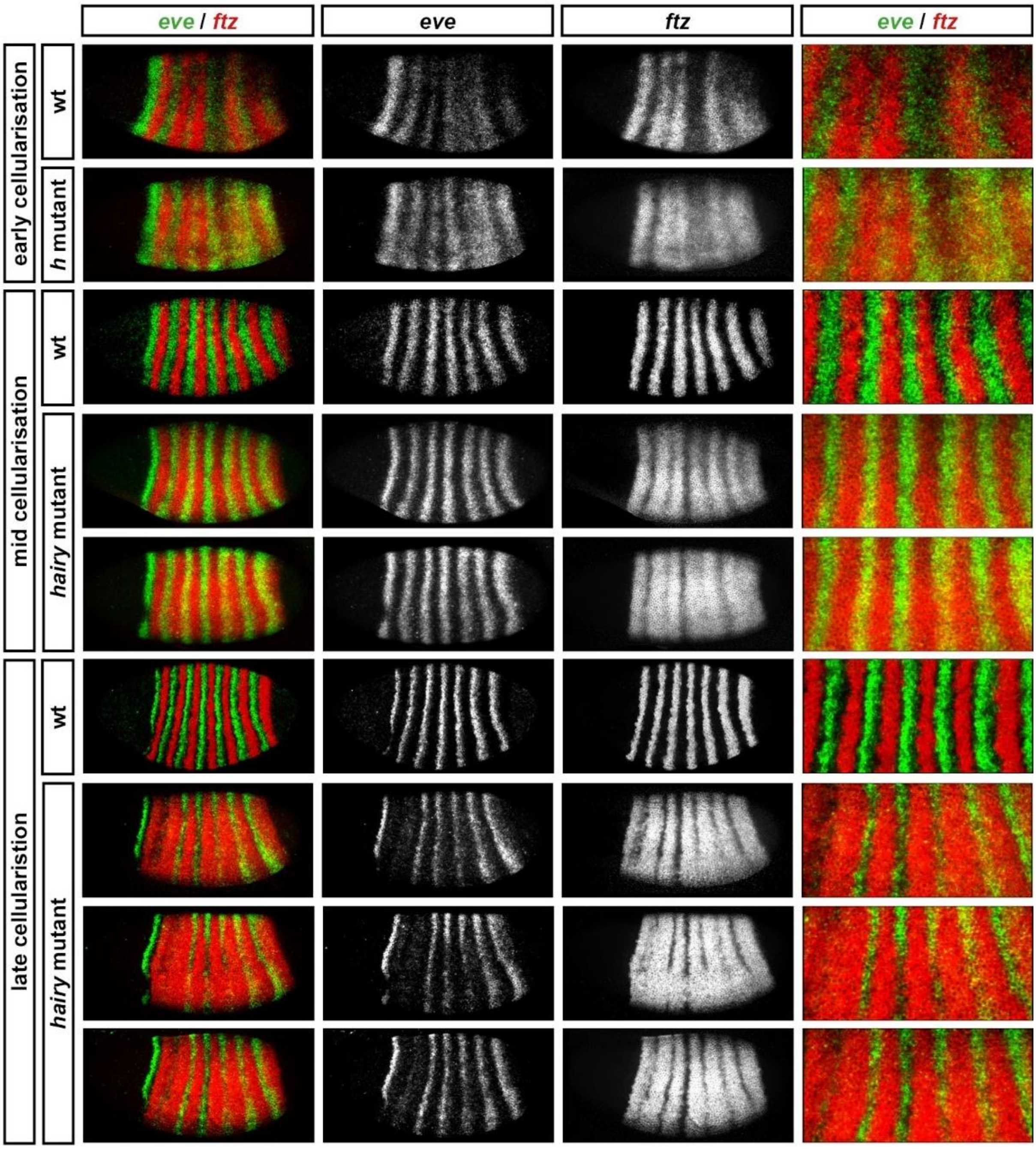

S1 Text–fig 3: *ftz* is regulated by Hairy but *eve* is not

Relative expression of *ftz* and *eve* in wild-type and *hairy* mutant embryos at early, mid, and late cellularisation. *ftz* expression expands dramatically in the mutant embryos, while *eve* expression is normal until late cellularisation. Two different mutant embryos are shown at mid-cellularisation, and three at late cellularisation. Single channel images are shown in greyscale in the central panels, and enlarged images of stripes 2-6 are shown at the right.

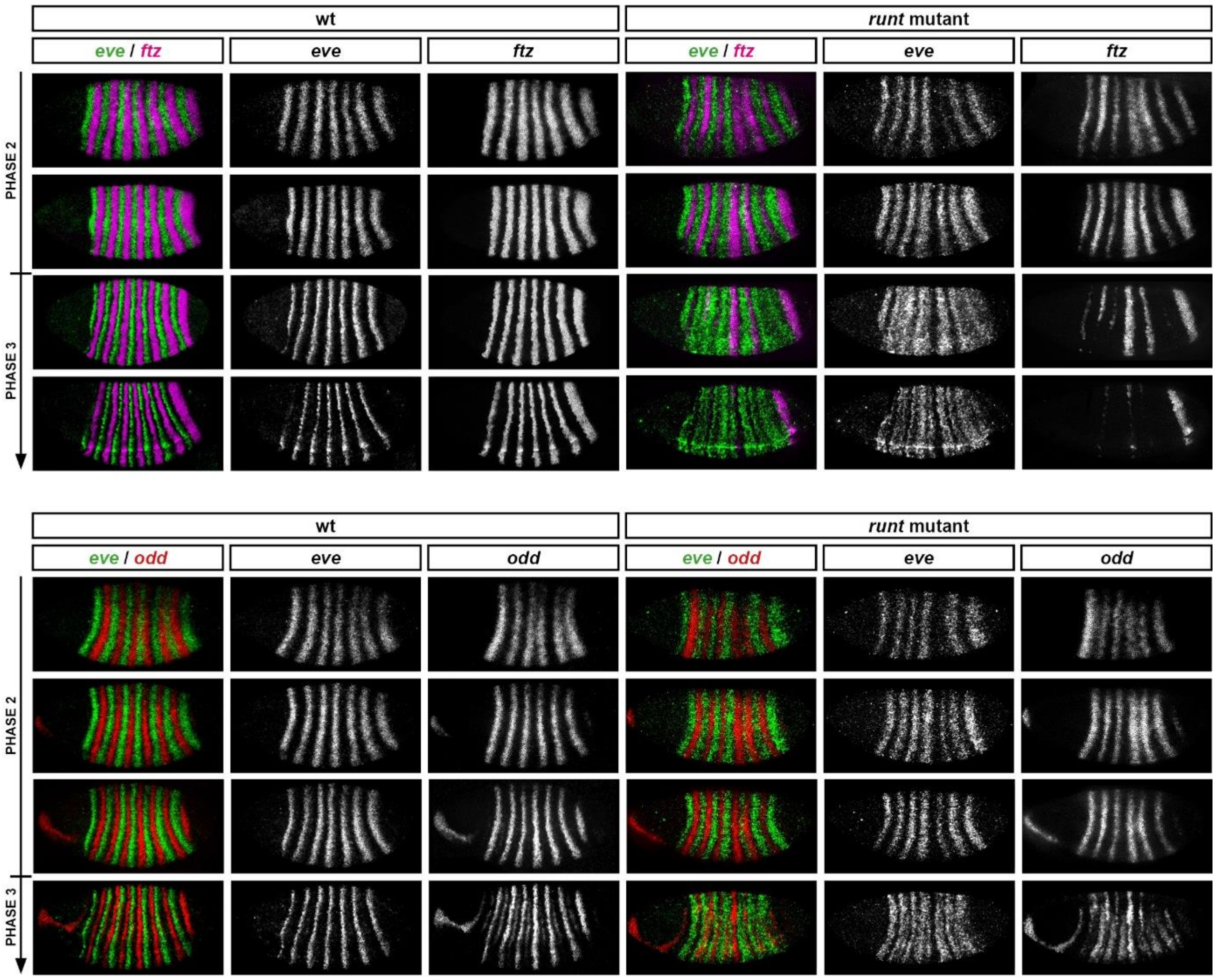

S1 Text–fig 4: *ftz* and *odd* expression remains out of phase with the *eve* stripes in *runt* mutant embryos, despite irregularities in stripe spacing

Expression of *ftz* and *odd* relative to *eve* in wild-type and *runt* mutant embryos. Note that strong expression of *ftz* and *odd* stripes 4 and 5 in *runt* mutant embryos corresponds to an absence of *eve* and *hairy* expression in these regions (compare S1 Text–fig 2). (These stripes fade only at gastrulation, presumably due to repression from newly synthesised Slp protein.) Single channel images are shown in greyscale to the right of the double channel images. Arrows indicate increasing developmental age.

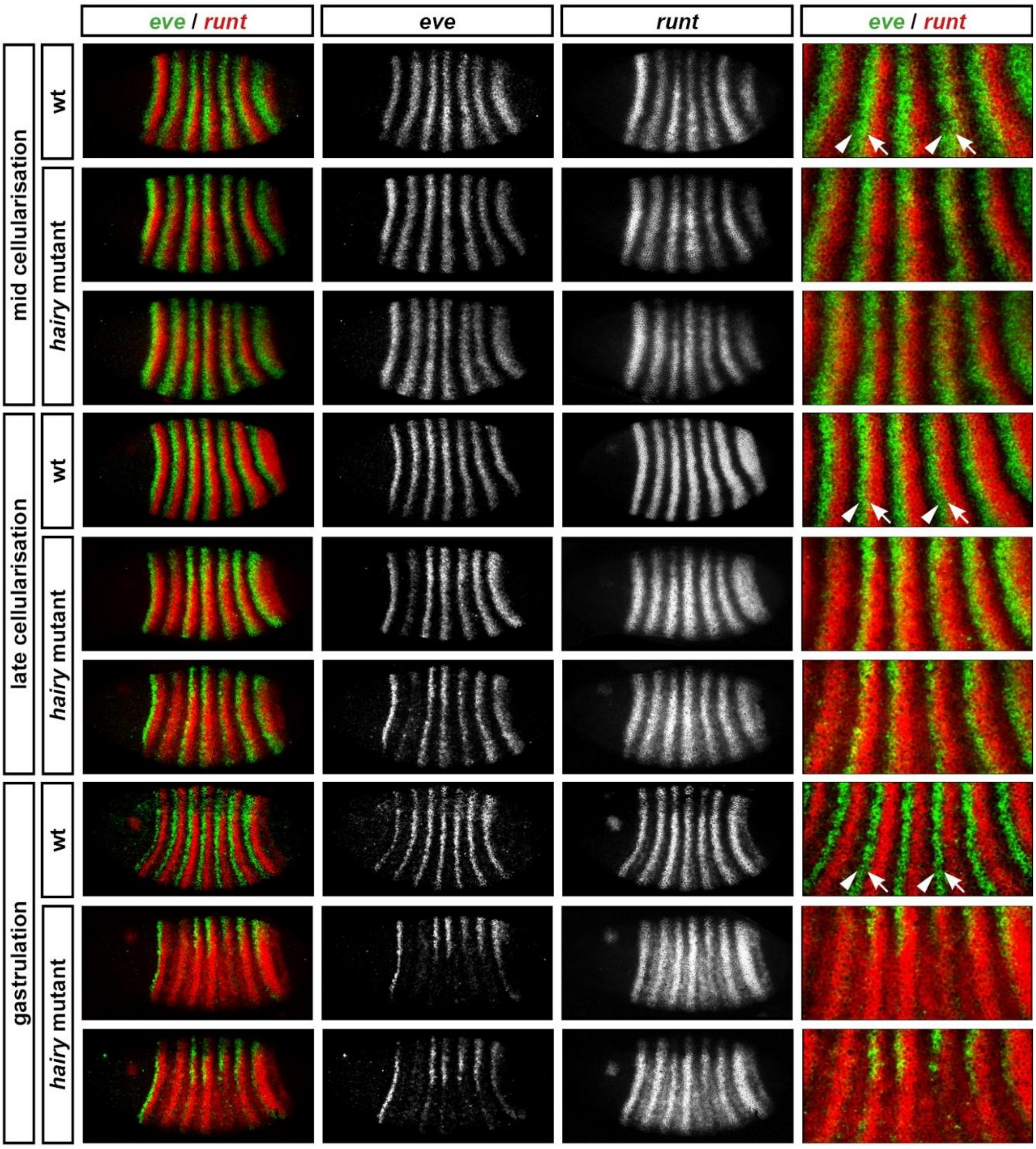

S1 Text–fig 5: *runt* stripes are shifted anteriorly in *hairy* mutant embryos compared to wild-type

Relative expression of *eve* and *runt* in wild-type and *hairy* mutants, at mid-cellularisation, late cellularisation, and gastrulation. Two different mutant embryos are shown for each time point. Arrowheads in wild-type embryos indicate the anterior border of an *eve* stripe; arrows indicate the anterior border of a *runt* stripe. In the mutant embryos the *runt* stripes shift anteriorly and eventually entirely overlap the whole width of the *eve* stripes. In the mutant embryos, *eve* stripe 2 is repressed at late-cellularisation, however *eve* stripes 3-7 are not lost until gastrulation. Note the low level *runt* expression in between the stripes, which appears from late cellularisation in the mutant embryos. Single channel images are shown in greyscale in the central panels, and enlarged views of stripes 2-6 are shown at the right.

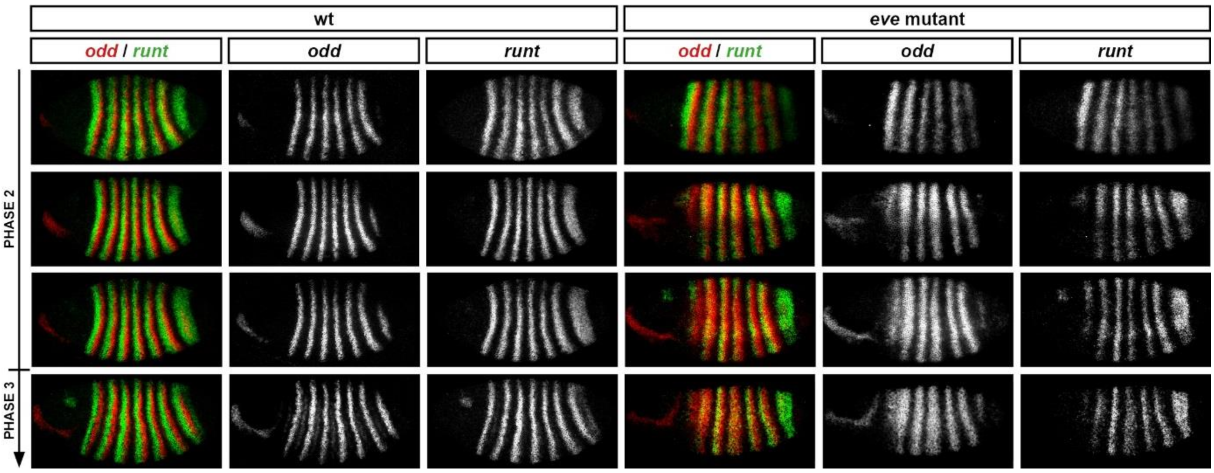

S1 Text–fig 6: *odd* derepression mediates repression of *runt* in *eve* mutant embryos during cellularisation.

Relative expression of *runt* and *odd* in wild-type and *eve* mutant embryos during phase 2. *odd* stripes are expanded anteriorly in *eve* mutant embryos, overlapping the whole width of the runt stripes, rather than just their posteriors as in wild-type. *runt* expression in stripes 1-6 is downregulated in *eve* mutant embryos compared to wild-type embryos, due to repression from the ectopic Odd. Single channel images are shown in greyscale to the right of the double channel images. Arrow indicates increasing developmental age.

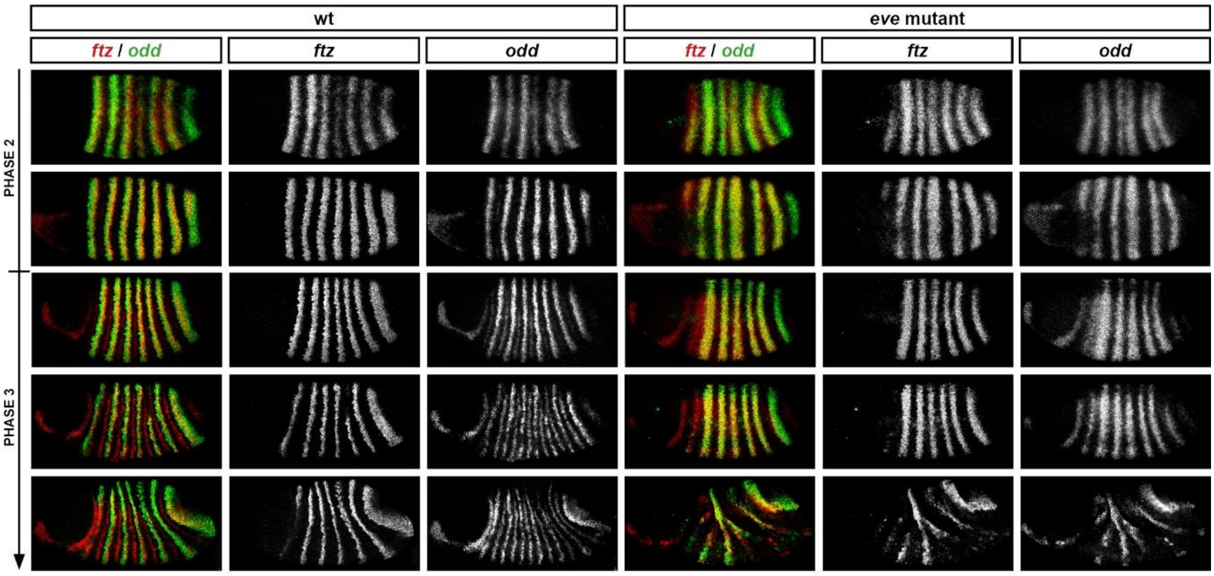

S1 Text–fig 7: *ftz* and *odd* are expressed very similarly to each other in *eve* mutant embryos.

Relative expression of *ftz* and *odd* in wild-type and *eve* mutant embryos. Stripes 2-6 of the two genes coincide exactly from phase 2 onwards. Single channel images are shown in greyscale to the right of the double channel images. Arrow indicates increasing developmental age.

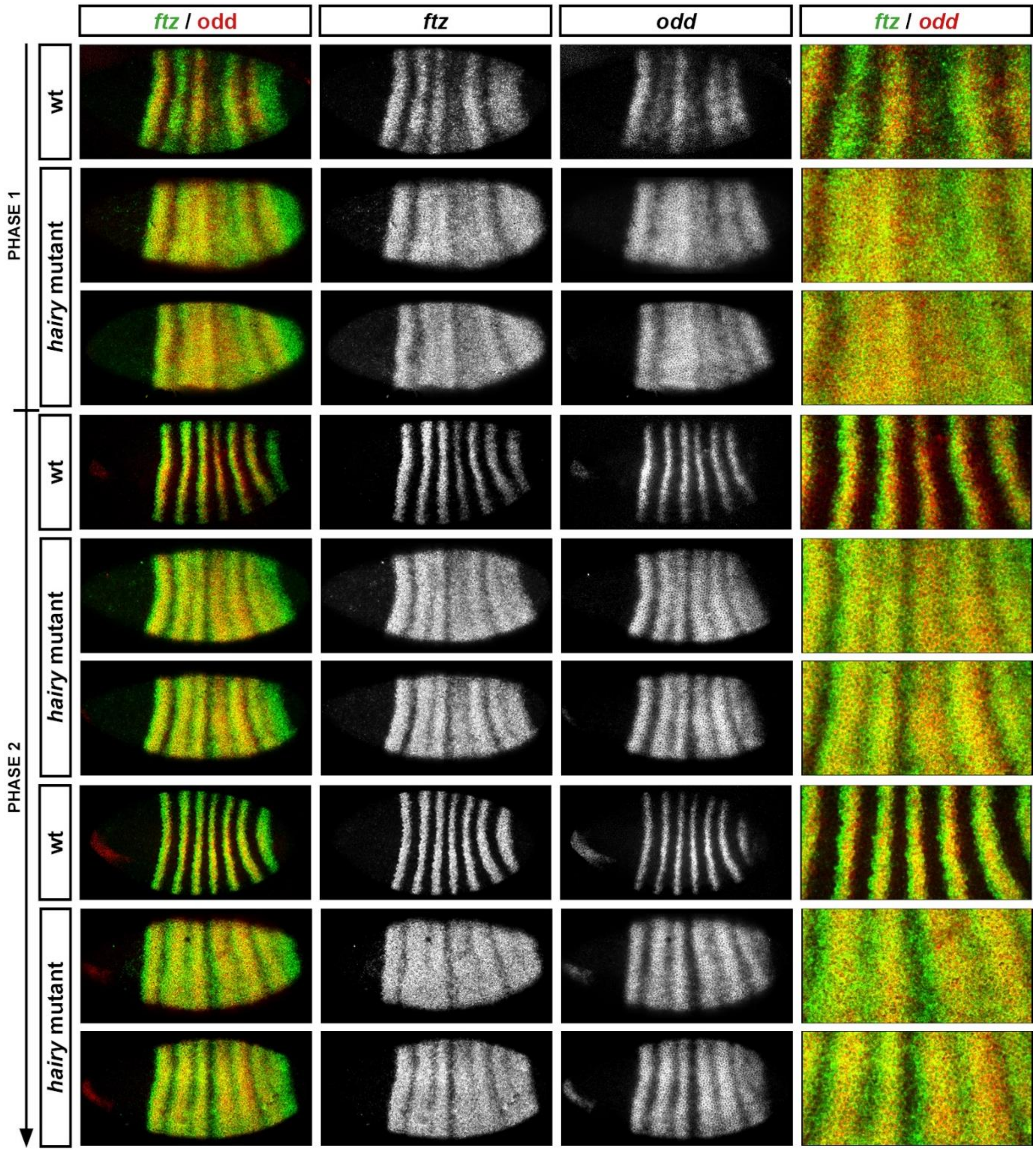

S1 Text–fig 8: *ftz* and odd are ectopically expressed throughout the trunk in *hairy* mutant embryos

Relative expression of *ftz* and *odd* in wild-type and *hairy* mutant embryos during the first half of cellularisation. Broad ectopic expression of both genes appears early. Single channel images are shown in greyscale to the right of the double channel images. Arrow represents increasing developmental age (phase 1 until mid-phase 2).
