# Supplementary material for: Dynamic patterning by the Drosophila pair-rule network reconciles long-germ and short-germ segmentation
Source: PLoS Biol. 2017 Sep 27;15(9):e2002439. doi: 10.1371/journal.pbio.2002439 (PMC5633203; doi:10.1371/journal.pbio.2002439)
Supplement: S2 Text — (PDF) [file pbio.2002439.s009.pdf]

Supplementary Text 2:

Details of models and simulations

This supplementary document provides details of the gene regulatory network models and simulation algorithm used to generate the simulations shown in Movies 1-13. Output data from these simulations are also presented in several of the main text figures.

I first provide overviews of the general modelling framework (theory), and its specific implementation (software). I then list the details (control logic, time delay values, and initial conditions) of each individual simulation. Where necessary, I discuss the way in which I have translated inferred genetic interactions into a formalised model, pointing out any ad hoc or uncertain assumptions. I also highlight situations where the regulation in the embryo is clearly more nuanced than can be captured by the simple models I use.

General modelling framework

I use a Boolean network approach [1–5] to model the interactions between a number of transcription factors in a given cell. In order to capture some of the essential "biology" of gene expression, cell state is separated into two distinct layers, roughly equivalent to transcript and protein respectively [6]. The current state of the "protein" layer determines the transcriptional output from the cell at the next timepoint, based on the control logic of the network. The state of the "transcript" layer is in turn converted into protein state at some later timepoint. Each component of the network is associated with specific time delays that govern the conversion process between transcript state and protein state, allowing different gene products to exhibit different "synthesis speeds" and/or "protein stability". Given some set of initial conditions, cell state will evolve deterministically over time, eventually arriving at a stable state or a limit cycle.

Each component in the network (i.e. each transcription factor) is represented by a Boolean variable. If the variable equals 0, the component is "off", while if the variable equals 1, the protein is "on". Given n components in the network, there are therefore 2^n possible network states. (If helpful) these possible states can be thought of as the vertices of an n-dimensional hypercube. A particular network state (protein state) can be specified by a one-dimensional array of length n, where the value (0 or 1) of index i gives the expression state of the ith component in the network. For example, for a network of three gene products A,B, and C, the array [1,0,1] would represent the state where A and C are on, and B is off.

The transcriptional output of each particular component in the network can be calculated from the current protein state array by a specific logical function (the "control logic" of the gene). Each network component will have its own logical function, which may take into account the protein states of all, some, or none of the components in the network, including itself. There is no constraint on the form of the logical functions, so long as they map each of the 2^n possible network states to a specified level of transcription (0 or 1). Combinatorial regulatory interactions are therefore easily represented, although quantitative regulatory effects are ruled out by definition.

Stable states of the network can be easily calculated from the overall set of protein state → transcriptional output mappings; they are those where the output state for each component is unchanged from its input state. These stable attractor states can be thought of as representing particular cell "fates".

The network is updated synchronously, by discrete timesteps. The transcriptional output of the network at timepoint $t+1$ is simply calculated from the network protein state at time t. However, calculating the new protein state is more complicated, because of the time delays that need to be accounted for.

Each component in the network is associated with two positive integer parameter values, a "synthesis delay"(s) and a "decay delay" (d). The synthesis delay determines how long a component must be transcribed before the component turns on at the protein level, while the decay delay determines how long a component has to be transcriptionally silenced before the component turns off. Each component is also associated with two non-negative integer variables, "transcript age" and "protein age" respectively, that provide some "memory" to the system.

The particular rules governing the conversion process for a given network component are as follows:

if (*transcript*=0 and *old_protein*=0) then *new_protein* → 0, *transcript_age* →

if (*transcript*=1 and *old_protein*=0) then increment *transcript_age*:

> if (*transcript_age* < *synthesis_delay*) then *new_protein* → 0; else *new_protein* → 0

if (*transcript*=1 and *old_protein*=1) then *new_protein* → 1, *protein_age* → 0

if (*transcript*=0 and *old_protein*=1) then *transcript_age* → 0, increment *protein_age*:

> if (*protein_age* < *decay_delay*) then *new_protein* → 1; else *new_protein* → 0, protein_age → 0

Note: "*transcript*" = transcriptional output at timepoint $t+1$; "*old_protein*" = protein state at timepoint t; "*new_protein*" = protein state at timepoint $t+1$.

The simulation algorithm is thus set up so that a protein will only turn on if there is continuous transcription for the entire duration of the synthesis delay, and will only turn off is transcription is continuously absent for the entire duration of the decay delay, effectively ignoring very brief changes to transcriptional output.

Implementation

The simulation software is written in object-oriented Python (www.python.org), using the additional libraries NumPy and Matplotlib [7,8]. All the functions necessary for producing and visualising a simulation are contained in one .py file (S1 File). The user simply specifies a network model and simulation parameter values in a separate script (see S2 File for a template), and can thereby easily carry out bespoke simulations. Simulations can be run

simultaneously for an arbitrary number of "cells", each of which behaves independently. Each cell has the same network model and parameter values, but can be assigned different initial conditions.

The things the user must specify are:

1) The network components (i.e. the various transcription factors in the system)
2) The control logic (i.e. regulation) of each component. This is done simply by defining a function that takes the current state of the network as its argument and returns either 0 or 1 as appropriate.
3) The synthesis and decay delays associated with each component. There are also functions to set default values for all components, if desired.
4) The number of "cells" to be simulated.
5) The initial conditions of each cell. Protein state, protein age, transcript state, and transcript age may all be specified. Values are set to zero by default.
6) The number of timepoints to simulate.
7) If visualising the output with an animation, the colour associated with each gene product, and other aesthetic preferences.

The user then simply has to call the simulation function to calculate the system state at each timepoint, given the chosen initial conditions, control logic, and parameter values. The user may then export or visualise the output data.

Visualisations represent individual cells as different columns (named C1, C2, C3…) and different gene products as different named rows. Both "transcript" and "protein" outputs are shown for each row. Depending on user preference, transcript and protein ages can also be represented in the visualisation, with "decaying" proteins shown as increasingly transparent before they turn off, and "nascent" transcripts shown as increasingly opaque until protein appears.

Visualisations can be shown as an animation, or the individual frames may be saved as image files. The frames can then be stitched together to produce video files, for example using ffmpeg (www.ffmpeg.org).

Pair-rule model details

Each simulation referenced in the main text models pair-rule gene expression along an idealised anteroposterior axis. The simulations show an array of 20 "cells", each of which evolves independently. This cellular autonomy is justified for the model by the apical localisation of real pair-rule transcripts, which precludes diffusion between nuclei during cellularisation of the blastoderm [9,10]. (Note also that all the pair-rule genes code for transcription factors, rather than e.g. components of signalling pathways.) Initial conditions for each simulation consist of a pattern that repeats every 8 cells, with each group of 8 cells representing an idealised double segment repeat.

"Early network" simulations (Movies 1-8) are the simplest, restricted to modelling the establishment and maintenance of the original pair-rule pattern observed at mid-cellularisation. They model the expression of the five primary pair-rule genes (*hairy*, *eve*, *runt*, *ftz*, and *odd*), as patterned ultimately by abstracted gap inputs (e.g. "G1", "G2").

"Whole system" simulations (Movies 9-11) are more complicated, additionally including the patterning of the secondary pair-rule genes, and the transition to single-segment periodicity. As well as the primary pair-rule genes and the gap inputs, they model the secondary pair-rule genes *prd*, *slp*, and the segment-polarity gene *en*. They also utilise two temporal signals, Cad and Opa. Cad controls the onset of secondary pair-rule gene expression, while Opa controls the switch between the early and the late pair-rule networks.

"Modified network" simulations (Movies 12 and 13) are based on the "whole system" simulations, but tweak the inferred *Drosophila* network so as to produce a more autonomous patterning system that can operate in the absence explicit gap inputs.

In all simulations, the seven pair-rule factors and En are all assigned identical synthesis and decay delays, both equal to 6 timesteps. Studies of pair-rule gene expression kinetics indicate that protein synthesis and turnover rates in the embryo are equally rapid, and are both processes occurring on the order of 10 minutes [11,12]. The time delays associated with the remaining system components (gap inputs, Cad, and Opa) are chosen in an ad hoc manner so as to provide appropriate spatiotemporal signals to the pair-rule genes.

The control logic, time delays, and initial conditions for each simulation are described below. Note that any unspecified initial conditions can be assumed to be set to zero.

"Early network"simulations (Movies 1-8)

The core control logic of these simulations is the "early network" shown in Fig 1A (left) of the main text. It is formulated as below:

hairy: if (G1=ON) then *hairy* → OFF; else *hairy* → ON

eve: if (G2=ON) then *eve* → OFF; else *eve* → ON

runt: if (Hairy=ON or Odd=ON) then *runt* → OFF, else *runt* → ON

ftz: if (Hairy=ON or Eve=ON) then *ftz* → OFF, else *ftz* → ON

odd: if (Hairy=ON or Eve=ON) then *odd* → OFF, else *odd* → ON

In each simulation, no initial pair-rule gene expression is specified (i.e. in each cell, for each of the five variables, the initial conditions are transcript=0, protein=0, transcript age=0, protein age=0).

Effects of shifting gap inputs (simulations 1-5)

Simulations 1 to 5 explore the effects of dynamic gap inputs on pair-rule gene expression. The only differences between them relate to the "gap inputs" G1 and G2.

Control logic:

G1 and G2 autoactivate in simulation 1 so as to produce stable domains, and autorepress in simulations 2-5 so as to produce dynamic domains.

Simulation 1 (static gap inputs):

g1: if (G1=ON) then *g1* → ON; else *g1* → OFF

g2: if (G2=ON) then *g2* → ON; else *g1* → OFF

Simulations 2-5 (dynamic gap inputs):

g1: if (G1=ON) then *g1* → OFF; else *g1* → ON

g2: if (G2=ON) then *g2* → OFF; else *g1* → ON

Time delays:

In each of the simulations 2-5, the time delays and initial conditions of G1 and G2 are specifically chosen so as to produce a particular gap domain shift rate while preserving an 8 cell pattern repeat. Simulation 2 produces a shift rate of one cell every 6 timesteps (equal to the synthesis/decay delay of the pair-rule factors), which requires G1/G2 expression to go through a full oscillation every 48 timesteps. Simulations 3-5 have time delays and initial conditions equivalent to those of simulation 2, except multiplied by a scaling factor to give slower or faster oscillations of G1/G2 in each cell and hence slower or faster shifts. Simulation 3 is scaled by 2, Simulation 4 is scaled by 1/2, and Simulation 5 is scaled by 1/3.

	**Sim 1**	**Sim 2**	**Sim 3**	**Sim 4**	**Sim 5**
**G1/G2 synthesis delay**	6	18	36	9	6
**G1/G2 decay delay**	6	30	60	15	10

Intitial conditions:

Common to all simulations:

	**C1**	**C2**	**C3**	**C4**	**C5**	**C6**	**C7**	**C8**
**G1 value**	0	0	0	1	1	1	1	1
**G2 value**	1	1	0	0	0	1	1	1

Common to simulations 2-4:

	**C1**	**C2**	**C3**	**C4**	**C5**	**C6**	**C7**	**C8**
***g1* value**	1	1	1	0	0	0	0	0
***g2* value**	0	0	1	1	1	0	0	0

Specific to simulation 2:

	C1	C2	C3	C4	C5	C6	C7	C8
***g1* age**	0	6	12	0	0	0	0	0
**G1 age**	0	0	0	0	6	12	18	24
***g2* age**	0	0	0	6	12	0	0	0
**G2 age**	18	24	0	0	0	0	6	12

Specific to simulation 3:

	C1	C2	C3	C4	C5	C6	C7	C8
***g1* age**	0	12	24	0	0	0	0	0
**G1 age**	0	0	0	0	12	24	36	48
***g2* age**	0	0	0	12	24	0	0	0
**G2 age**	36	48	0	0	0	0	12	24

Specific to simulation 4:

	C1	C2	C3	C4	C5	C6	C7	C8
***g1* age**	0	3	6	0	0	0	0	0
**G1 age**	0	0	0	0	3	6	9	12
***g2* age**	0	0	0	3	6	0	0	0
**G2 age**	9	12	0	0	0	0	3	6

Specific to simulation 5:

	C1	C2	C3	C4	C5	C6	C7	C8
***g1* age**	0	2	4	0	0	0	0	0
**G1 age**	0	0	0	0	2	4	6	8
***g2* age**	0	0	0	2	4	0	0	0
**G2 age**	6	8	0	0	0	0	2	4

Effects of additional gap inputs (simulations 6-8)

These three simulations model situations where *runt* and/or *ftz*/*odd* are initially controlled by gap inputs, before being taken over the pair-rule network later on. These simulations involve some extra components. Simulation 6 has an additional gap input, G3, which regulates *runt*, while simulation 7 has an analogous gap input, G4, which regulates *ftz* and *odd*. Both G3 and G4 are present in simulation 8. All three simulations additionally have a temporal signal, "G" which controls whether *runt* and/or *ftz*/*odd* are controlled by the gap inputs G3/G4, or alternatively by pair-rule inputs as normal. Details of any new control logic and initial conditions are listed below. Anything not explicitly detailed is the same as simulation 2.

Control logic:

Simulations 6-8:

g: *g* → OFF

Simulations 6 and 8:

g3: if (G3=ON) then *g3* → OFF; else *g3* → ON

runt: if (G=ON):

if (G3=ON) then *runt* → OFF; else *runt* → ON

if (G=OFF):

if (Hairy=ON or Odd=ON) then *runt* → OFF, else *runt* → ON

Simulations 7 and 8:

g4: if (G4=ON) then *g4* → OFF; else *g4* → ON

ftz: if (G=ON):

if (G4=ON) then ftz → OFF; else *ftz* → ON

if (G=OFF):

if (Hairy=ON or Eve=ON) then *ftz* → OFF, else *ftz* → ON

odd: if (G=ON):

if (G4=ON) then *odd* → OFF; else *odd* → ON

if (G=OFF):

if (Hairy=ON or Eve=ON) then *odd* → OFF, else *odd* → ON

Time delays:

	Sim 6	Sim 7	Sim 8
**G decay delay**	12	6	6
**G3 synthesis delay**	18	-	18

**G4 synthesis delay**	-	18	18
**G3 decay delay**	30	-	30
**G3 decay delay**	-	30	30

Note: the G decay delay effectively specifies how long *runt* and/or *ftz*/*odd* are controlled by gap inputs rather than the pair-rule network.

Initial conditions:

Common to simulations 6-8:

	**C1**	**C2**	**C3**	**C4**	**C5**	**C6**	**C7**	**C8**
**G value**	1	1	1	1	1	1	1	1

Specific to simulations 6 and 8:

	**C1**	**C2**	**C3**	**C4**	**C5**	**C6**	**C7**	**C8**
***g3* value**	0	0	0	0	1	1	1	0
***g3* age**	0	0	0	0	0	6	12	0
**G3 value**	1	1	1	1	0	0	0	1
**G3 age**	6	12	18	24	0	0	0	0

Specific to simulations 7 and 8:

	**C1**	**C2**	**C3**	**C4**	**C5**	**C6**	**C7**	**C8**
***g4* value**	1	0	0	0	0	0	1	1
***g4* age**	12	0	0	0	0	0	0	6
**G4 value**	0	1	1	1	1	1	0	0
**G4 age**	0	0	6	12	18	24	0	0

<u>Whole system simulations (Movies 9-11)</u>

Control logic:

The structure of the pair-rule network as a whole is based largely on evidence from [13], and summarised in Fig 1A of the main text. The topology of the network is context dependent, with many genetic interactions affected by the transcriptional cofactor, Opa. Since Opa

protein appears in the *Drosophila* embryo only at the end of cellularisation [14], segment patterning is sequentially directed by “early” and “late” incarnations of the overall network.

I therefore make the control logic of each segmentation gene in the network conditional on the presence or absence of Opa. If Opa is off, the control logic is a formulation of the early network, while if Opa is on, the control logic is a formulation of the late network. The specific regulatory rules for each pair-rule gene (plus *en*) are described below, supplemented with explanatory notes where appropriate.

hairy: if (Opa=OFF):

if (G1=ON) then *hairy* → OFF; else *hairy* → ON

if (Opa=ON):

hairy → OFF

Notes: While Opa is off, the regulation of *hairy* is the same as in Simulations 1-8. As soon as Opa turns on, *hairy* expression turns off. Hairy protein plays no role in the late pair-rule network and is independent of pair-rule gene expression throughout the whole patterning process.

eve: if (Opa=OFF):

if (G2=ON) then *eve* → OFF; else *eve* → ON

if Opa=ON:

if (Runt=ON or Odd=ON or En=ON or (Slp=ON and Eve=OFF)) then *eve* → OFF; else *eve* → ON

Notes: While Opa is off, the regulation of *eve* is the same as in Simulations 1-8. As soon as Opa turns on, *eve* expression is instead patterned by various repressors (Runt, Odd, Slp, En). The qualification in the model that Slp represses *eve* only if Eve is not already turned on is required in order to resolve overlaps between *eve* and *slp* expression that form in the simulations just before the switch to the late network occurs. Specifying that Eve expression “wins” this contest ensures that the anterior boundaries of the Eve stripes define the parasegment boundaries, as occurs in the embryo [15]. However, the overlap situation is not a scenario that occurs in real embryos: the *eve* boundaries stabilise at roughly the same time *slp* turns on (rather than afterwards), and therefore the *eve* and *slp* domains are mutually exclusive throughout the segmentation process. A more realistic model that avoided this issue would need to include more sophisticated temporal control of *eve* and *slp* regulation, and/or time-dependent gap domain dynamics. Note that for simulation 11, which models an *eve* mutant embryo, the control logic of *eve* is replaced by unconditional repression (*eve* → OFF).

runt: if (Opa=OFF):

if (Hairy=ON or Odd=ON) then *runt* → OFF; else *runt* → ON

if (Opa=ON):

if ((Eve=ON and Runt=OFF) or (Odd=ON and Runt=OFF) or En=ON) then *runt* → OFF; else *runt* → ON

Notes: While Opa is off, the regulation of *runt* is the same as in Simulations 1-8. As soon as Opa turns on, *runt* expression is instead patterned by various repressors (Eve, Odd, En). However, I have specified that if Runt is coexpressed with either Eve or Odd, the Runt expression will "win" and *runt* transcription will not turn off. This is because Eve/Runt and Runt/Odd overlaps are both present during cellularisation (at the anteriors and the posteriors of the Runt stripes, respectively), and both resolve in favour of *runt* expression during gastrulation [13,16]. In contrast, En expression has the potential to supplant Runt expression, as occurs at the posterior borders of the *runt* primary stripes. Note that the inferred control logic is a phenomenological description of regulation within the embryo, and does not necessarily imply Runt autoregulation at a mechanistic level. Note also that the Opa-dependent control logic specified above reflects the late regulation of the *runt* "seven stripe element", and I have not explicitly modelled the expression arising from the "six stripe element" [13,17].

ftz: if (Opa=OFF):

if (Hairy=ON or Eve=ON or Slp=ON) then *ftz* → OFF; else *ftz* → ON

if (Opa=ON):

if (Eve=ON or Slp=ON or Ftz=OFF) then *ftz* → OFF; else *ftz* → ON

Notes: While Opa is off, the regulation of *ftz* is the same as for Simulations 1-8, except with the addition of repression from Slp. (Note that this last interaction is not diagrammed in Fig 1A, since Slp protein is synthesised too late to have any practical effect on *ftz* expression while the early network is in operation.) When Opa turns on, *ftz* expression remains repressed by Eve and Slp, but is no longer regulated by Hairy. Instead, it requires autoactivation. Having this autoregulation in the model permits the anterior border of the Ftz domain to remain stable and define the even-numbered parasegment boundaries, as occurs in the embryo [15]. However, note that while direct Ftz autoactivation is well-documented [18,19], it remains to be tested whether Ftz activity is absolutely required for late Ftz expression.

odd: if (Opa=OFF):

if (Hairy=ON or Eve=ON or Slp=ON) then *odd* → OFF; else *odd* → ON

if (Opa=ON):

if (Runt=ON or Slp =ON or En=ON or (Prd=ON and Odd=OFF)) then *odd* → OFF; else *odd* → ON

************* Odd repressed by Slp in Opa positive

************* Add Cad to conditions

Notes: While Opa is off, the regulation of *odd* is the same as for Simulations 1-8, except with the addition of repression from Slp. (Note that this last interaction is not diagrammed in Fig 1A, since Slp protein is synthesised too late to have any practical effect on *odd* expression while the early network is in operation.) When Opa turns on, *odd* expression is no longer regulated by Hairy or Eve, and instead becomes repressed by Runt, En, and Prd. It remains repressed by Slp. I have specified that the repression by Prd only occurs if Odd expression is not already turned on – this reflects the observation that Prd activity patterns the nascent *odd* secondary stripes [20], but has no apparent effect on the *odd* primary stripes. This phenomenon – which does not necessarily imply Odd autoregulation at the mechanistic level – should be further investigated to test the inferred control logic.

prd: if (Opa=OFF):

if (Cad=ON or Eve=ON) then *prd* → OFF; else *prd* → ON

if (Opa=ON):

if (Prd=ON and Odd=OFF) then *prd* → ON; else *prd* → OFF

Notes: While Opa is off, *prd* is repressed by Eve (which patterns where it is expressed) and by Cad (which patterns *when* it is expressed). When Opa turns on, *prd* is no longer repressed by Eve, and instead repressed by Odd. It also requires autoactivation. The autoregulation is required in the model in order to maintain a stable posterior boundary within the odd-numbered parasegments. Note that while early repression by Eve and late repression from Odd are both strongly supported by experimental evidence, not much more is known about *prd* regulation. The control logic above is therefore largely speculative, and indeed does not perform particularly well in the simulations (see S3 Fig). The regulation of *prd* therefore warrants further investigation.

slp: if (Opa=OFF):

if (Cad=ON or Eve=ON or Runt=ON or Prd=OFF) then *slp* → OFF; else *slp* → ON

if (Opa=ON):

if (Eve=ON or En=ON or ((Ftz=ON or Odd=ON) and Slp=OFF)) then *slp* → OFF; else *slp* → ON

Notes: While Opa is off, *slp* is repressed by Eve, Runt, and Cad, and requires activation by Prd. Slp expression is thus spatially patterned by Eve and Runt, and temporally patterned by Cad and Prd. The requirement for activation by Prd means that *slp* expression follows *prd* expression after a short time lag, in agreement with real expression in the embryo. This stipulation in the model is not entirely ad hoc: *slp* expression is reduced in *prd* mutant embryos (data not shown), indicating that *prd* does contribute to *slp* activation. However, *slp* expression is not abolished in these embryos, indicating that other, as yet unidentified, temporal signals must also be involved in the process. When Opa turns on, *slp* is patterned by repression from Eve, En, Ftz, and Odd. The qualification that Ftz and Odd only repress *slp* if Slp expression is absent reflects the observation that expression overlaps between Slp and

Ftz/Odd at the posteriors of the Ftz/Odd primary stripes are always resolved in favour of Slp (see Fig 7E in the main text). Again, note that this phenomenological description does not necessarily imply Slp autoregulation at the mechanistic level.

en: if (Opa=OFF):

en → OFF

if (Opa=ON):

if (Ftz=ON and Odd=OFF and Slp=OFF) or (Prd=ON and Odd=OFF and Slp=OFF and Runt=OFF)) then *en* → ON; else *en* → OFF

Notes: While Opa is off, *en* expression is repressed. When Opa turns on, *en* is spatially patterned by both activators and repressors. *en* requires activation from either Prd or Ftz, and is always repressed by Odd and Slp. The odd-numbered *en* stripes (those activated by Prd) are additionally repressed by Runt. In contrast, the even-numbered *en* stripes (those activated by Ftz) are insensitive to Runt activity.

The remaining components of the model (Cad, Opa, G1/G2) provide broad or abstracted inputs into the periodically expressed components described above. Cad and Opa are extrinsic inputs that are "off" by default or "on" by default, respectively. G1 and G2 are abstracted, autoregulatory gap inputs, whose expression (and function) is restricted to early stages of patterning (i.e. when Opa is off). Note that G1 and G2 have different control logic in Simulation 9 (modelling static gap inputs) versus Simulations 10 and 11 (modelling dynamic gap inputs).

cad *cad* → OFF

opa: *opa* → ON

g1 (Sim 9): if (Opa=OFF):

if (G1=ON) then *g1* → ON; else *g1* → OFF

if (Opa=ON):

g1 → OFF

g1 (Sims 10,11): if (Opa=OFF):

if (G1=ON) then *g1* → OFF; else *g1* → ON

if (Opa=ON):

g1 → OFF

g2 (Sim 9): if (Opa=OFF):

if (G2=ON) then *g2* → ON; else *g2* → OFF

if (Opa=ON):

g2 → OFF

g2 (Sims 10,11): if (Opa=OFF):

if (G2=ON) then *g2* → OFF; else *g2* → ON

if (Opa=ON):

g2 → OFF

Time delays:

	**Sims 9-11**
**Cad decay delay**	24
**Opa synthesis delay**	36
**G1/G2 synthesis delay**	18
**G1/G2 decay delay**	30
**All other delays**	6

Initial conditions:

Simulation 9:

	**C1**	**C2**	**C3**	**C4**	**C5**	**C6**	**C7**	**C8**
**Cad value**	1	1	1	1	1	1	1	1
**G1 value**	0	0	0	1	1	1	1	1
**G2 value**	1	1	0	0	0	1	1	1

Simulations 10 and 11:

	**C1**	**C2**	**C3**	**C4**	**C5**	**C6**	**C7**	**C8**
**Cad value**	1	1	1	1	1	1	1	1
***g1* value**	1	1	1	0	0	0	0	0
***g1* age**	0	6	12	0	0	0	0	0

**G1 value**	0	0	0	1	1	1	1	1
**G1 age**	0	0	0	0	6	12	18	24
***g2* value**	0	0	1	1	1	0	0	0
***g2* age**	0	0	0	6	12	0	0	0
**G2 value**	1	1	0	0	0	1	1	1
**G2 age**	18	24	0	0	0	0	6	12

Modified network simulations (Movies 12-13)

These two simulations alter the control logic of *hairy* and *eve* and remove the gap inputs from the network. In addition, the control logic of *opa* is altered so as to be regulated by Cad. The time delays associated with Hairy, Cad, and Opa are also adjusted.

Under these modified conditions, the only initial conditions that need be specified are those of Hairy and Cad. They are assigned very different starting patterns in the two simulations, however the two simulations eventually generate the same final output pattern.

Control logic:

hairy: if (Opa=OFF):

if (Hairy=ON) then *hairy* → OFF; else *hairy* → ON

if (Opa=ON):

hairy → OFF

eve: if (Opa=OFF):

if (Runt=ON or Odd=ON) then *eve* → OFF; else *eve* → ON

if (Opa=ON):

if (Runt=ON or Odd=ON or En=ON or (Slp=ON and Eve=OFF)) then *eve* → OFF; *else* eve → ON

opa: if (Signal X=ON) then *opa* → OFF; else *opa* → ON

Time delays:

	synthesis	decay
**Hairy**	30	18
**Cad**	6	42 (sim. 12) or 144 (sim. 13)

**Opa**	18	6

Note that the decay delay for Cad is greatly increased for Simulation 13.

Initial conditions:

Simulation 12:

	C1	C2	C3	C4	C5	C6	C7	C8
**Cad value**	1	1	1	1	1	1	1	1
***hairy* value**	1	1	1	1	1	0	0	0
***hairy* age**	0	6	12	18	24	0	0	0
**Hairy value**	0	0	0	0	0	1	1	1
**Hairy age**	0	0	0	0	0	0	6	12

Simulation 13:

	C1	C2	C3	C4	C5	C6	C7	C8	C9	C10
**Hairy value**	1	1	1	1	1	1	1	1	1	1
**Cad value**	1	1	1	1	1	1	1	1	1	1
**Cad age**	114	108	102	96	90	84	78	72	66	60
	**C11**	**C12**	**C13**	**C14**	**C15**	**C16**	**C17**	**C18**	**C19**	**C20**
**Hairy value**	1	1	1	1	1	1	1	1	1	1
**Cad value**	1	1	1	1	1	1	1	1	1	1
**Cad age**	54	48	42	36	30	24	18	12	6	0

REFERENCES

1. Kauffman SA. Homeostasis and Differentiation in Random Genetic Control Networks. Nature. 1969;224:177–8.
2. Thomas R. Boolean formalization of genetic control circuits. J Theor Biol. 1973;42:563–85.
3. De Jong H. Modeling and Simulation of Genetic Regulatory Systems : A Literature Review. 2006;
4. Peter IS, Faure E, Davidson EH. Predictive computation of genomic logic processing functions in embryonic development. Proc Natl Acad Sci U S A. 2012;109:16434–42.

5. Mbodj A, Junion G, Brun C, Furlong EEM, Thieffry D. Logical modelling of Drosophila signalling pathways. Mol Biosyst. 2013;9:2248–58.

6. Thomas R. Regulatory Networks Seen as Asynchronous Automata : A Logical Description. J Theor Biol. 1991;1–23.

7. van der Walt S, Colbert SC, Varoquaux G. The NumPy Array: A Structure for Efficient Numerical Computation. Comput Sci Eng. 2011;13:22–30.

8. Hunter JD. Matplotlib: A 2D Graphics Environment. Comput Sci Eng. 2007;9:90–5.

9. Davis I, Ish-Horowicz D. Apical localization of pair-rule transcripts requires 3' sequences and limits protein diffusion in the Drosophila blastoderm embryo. Cell. 1991;67:927–40.

10. Edgar BA, Odell GM, Schubiger G. Cytoarchitecture and the patterning of fushi tarazu expression in the Drosophila blastoderm. Genes Dev. 1987;1:1226–37.

11. Edgar BA, Weir MP, Schubiger G, Kornberg T. Repression and turnover pattern fushi tarazu RNA in the early Drosophila embryo. Cell. 1986;47:747–54.

12. Nasiadka A, Krause HM. Kinetic analysis of segmentation gene interactions in Drosophila embryos. Development. 1999;126:1515–26.

13. Clark E, Akam M. Odd-paired controls frequency doubling in Drosophila segmentation by altering the pair-rule gene regulatory network. Elife. 2016;5:e18215.

14. Benedyk MJ, Mullen JR, DiNardo S. Odd-paired: A zinc finger pair-rule protein required for the timely activation of engrailed and wingless in Drosophila embryos. Genes Dev. 1994;8:105–17.

15. Lawrence PA, Johnston P. Pattern formation in the Drosophila embryo: allocation of cells to parasegments by even-skipped and fushi tarazu. Development. 1989;105:761–7.

16. Pisarev A, Poustelnikova E, Samsonova M, Reinitz J. FlyEx, the quantitative atlas on segmentation gene expression at cellular resolution. Nucleic Acids Res. 2009;37:560–6.

17. Klingler M, Soong J, Butler B, Gergen JP. Disperse versus compact elements for the regulation of runt stripes in Drosophila. Dev Biol. 1996;177:73–84.

18. Hiromi Y, Gehring WJ. Regulation and Function of the Drosophila Segmentation Gene fushi tarazu. Cell. 1987;50:963–74.

19. Schier A, Gehring W. Direct homeodomain–DNA interaction in the autoregulation of the fushi tarazu gene. Nature. 1992;356:804–7.

20. Mullen JR, DiNardo S. Establishing parasegments in Drosophila embryos: roles of the odd-skipped and naked genes. Dev Biol. 1995;169:295–308.
